# Identification and Characterization of a β-*N*-Acetylhexosaminidase with a Biosynthetic Activity from the Marine Bacterium *Paraglaciecola hydrolytica* S66^T^

**DOI:** 10.3390/ijms21020417

**Published:** 2020-01-09

**Authors:** Triinu Visnapuu, David Teze, Christian Kjeldsen, Aleksander Lie, Jens Øllgaard Duus, Corinne André-Miral, Lars Haastrup Pedersen, Peter Stougaard, Birte Svensson

**Affiliations:** 1 Department of Biotechnology and Biomedicine, Technical University of Denmark, Søltofts Plads, Building 224, DK-2800 Kgs. Lyngby, Denmark; david.teze@gmail.com; 2 Institute of Molecular and Cell Biology, University of Tartu, Riia 23, 51010 Tartu, Estonia; 3 Department of Chemistry, Technical University of Denmark, Kemitorvet, Building 207, DK-2800 Kgs. Lyngby, Denmark; chkje@kemi.dtu.dk (C.K.); jduus@kemi.dtu.dk (J.Ø.D.); 4 Department of Chemistry and Bioscience, Aalborg University, Fredrik Bajers Vej 7H, DK-9220 Aalborg, Denmark; al@kebony.com (A.L.); lhp@bio.aau.dk (L.H.P.); 5 Unité de Fonctionnalité et Ingénierie des Protéines (UFIP), UMR CNRS 6286, Université de Nantes, F-44000 Nantes, France; corinne.miral@univ-nantes.fr; 6 Department of Plant and Environmental Sciences, University of Copenhagen, Thorvaldsensvej 40, DK-1871 Frederiksberg C, Denmark; pst@envs.au.dk

**Keywords:** *N*-acetylhexosamine specificity, glycoside hydrolase, GH20, phylogenetic analysis, transglycosylation, NAG-oxazoline, acceptor diversity, lacto-*N*-triose II, human milk oligosaccharides, NMR

## Abstract

β-*N*-Acetylhexosaminidases are glycoside hydrolases (GHs) acting on *N*-acetylated carbohydrates and glycoproteins with the release of *N*-acetylhexosamines. Members of the family GH20 have been reported to catalyze the transfer of *N*-acetylglucosamine (GlcNAc) to an acceptor, i.e., the reverse of hydrolysis, thus representing an alternative to chemical oligosaccharide synthesis. Two putative GH20 β-*N*-acetylhexosaminidases, *Ph*Nah20A and *Ph*Nah20B, encoded by the marine bacterium *Paraglaciecola hydrolytica* S66^T^, are distantly related to previously characterized enzymes. Remarkably, *Ph*Nah20A was located by phylogenetic analysis outside clusters of other studied β-*N*-acetylhexosaminidases, in a unique position between bacterial and eukaryotic enzymes. We successfully produced recombinant *Ph*Nah20A showing optimum activity at pH 6.0 and 50 °C, hydrolysis of GlcNAc β-1,4 and β-1,3 linkages in chitobiose (GlcNAc)_2_ and GlcNAc-1,3-β-Gal-1,4-β-Glc (LNT2), a human milk oligosaccharide core structure. The kinetic parameters of *Ph*Nah20A for *p*-nitrophenyl-GlcNAc and *p*-nitrophenyl-GalNAc were highly similar: *k*_cat_/*K*_M_ being 341 and 344 mM^−1^·s^−1^, respectively. *Ph*Nah20A was unstable in dilute solution, but retained full activity in the presence of 0.5% bovine serum albumin (BSA). *Ph*Nah20A catalyzed the formation of LNT2, the non-reducing trisaccharide β-Gal-1,4-β-Glc-1,1-β-GlcNAc, and in low amounts the β-1,2- or β-1,3-linked trisaccharide β-Gal-1,4(β-GlcNAc)-1,*x*-Glc by a transglycosylation of lactose using 2-methyl-(1,2-dideoxy-α-d-glucopyrano)-oxazoline (NAG-oxazoline) as the donor. *Ph*Nah20A is the first characterized member of a distinct subgroup within GH20 β-*N*-acetylhexosaminidases.

## 1. Introduction

A new marine bacterial species *Paraglaciecola hydrolytica* S66^T^ of the family *Alteromonadaceae* isolated from eelgrass (*Zostera* sp.) was shown by genome-sequencing [1] to encode 270 protein modules potentially acting on carbohydrates, 188 of which belong to enzyme families involved in degradation of carbohydrates [2,3]. The algal polysaccharides agar, agarose, alginate, porphyran or laminarin, but not carrageenans, fucoidan and ulvan, sustained the growth of *P. hydrolytica* as a sole carbon source, and the bacterium also grew on the plant polysaccharides: starch, amylopectin, amylose, xylan and pectin [2]. Overall, the large number of encoded carbohydrate-active enzymes (CAZymes) [4] and the flexibility with regard to carbon source indicates a very promising potential of the genome of *P. hydrolytica* for the discovery of enzymes with rare or not yet described activities.

Enzymes hydrolyzing glycosidic bonds with the release of *N*-acetylglucosamine (GlcNAc) are in focus since these carbohydrate residues occur in vital complex glycans, such as milk oligosaccharides and glycosphingolipids, for which there is a great demand [5]. Human milk oligosaccharides (HMOs) in particular are considered beneficial and needed for research and clinical trials within nutrition and as ingredients in functional foods and infant formulas [6,7,8]. HMOs are also regarded as emerging prebiotics or novel foods with positive health effects [9,10]. However, the chemical and enzymatic production of HMOs and their precursors or purification from natural sources are problematical [6,11,12], which creates bottlenecks for assessing the functional roles and applications of HMOs [13,14,15].

Lacto-*N*-triose II (LNT2, β-GlcNAc-1,3-β-Gal-1,4-Glc) is an HMO core structure in which *N*-acetylglucosamine is β-1,3-linked to lactose [6,16,17]. A few β-*N*-acetylhexosaminidases (β-NAHAs; EC 3.2.1.52) of the glycoside hydrolase family 20 (GH20) from bacteria, fungi and plants are reported to produce HMO-type GlcNAc-containing oligosaccharides with 1,3 linkages [15,18,19], as well as chitooligosaccharides and their analogs in transglycosylation reactions with the formation of 1,6 rather than 1,4 linkages [18,20,21,22]. In Nature, β-NAHAs from GH3, 20, 84, 109 and 116 [5,23,24] categorized in the CAZy database (www.cazy.org) [4] degrade *N*-acetylhexosamine-containing compounds by releasing GlcNAc and GalNAc from the non-reducing ends of *N*-acetylglucosides, *N*-acetylgalactosides, glycosphingolipids and glycoproteins [5,25,26,27]. Interestingly, these families display a variety of mechanisms, either retaining via a substrate-assisted mechanism (GH20 and GH84) [28,29] or a glycosyl-enzyme intermediate (GH3 and GH116) [30], or inverting via an oxidized form of nicotinamide adenine dinucleotide (NAD^+^)-depending mechanism (GH109) [31]. While being represented in five distinct GH families, the large majority of β-NAHAs belong to GH20.

*N*-acetylated oligo- and polysaccharides, e.g., chitooligosaccharides and chitin, are prevalent in marine organisms, thus crustaceans represent an abundant source of GlcNAc in marine environments. The National Center for Biotechnology Information (NCBI) database (https://www.ncbi.nlm.nih.gov/) currently has more than 112,000 predicted β-NAHAs, but out of the more than 200 characterized EC 3.2.1.52 enzymes (www.brenda-enzymes.org) [32], only a small number are of marine origin [21,26,33,34,35,36]. Accordingly, only six out of the 133 characterized GH20 β-NAHAs are from a marine organism (from www.cazy.org, 21st of November, 2019) even though a large number of sequences, also of marine origin, are annotated in genomes and metagenomes. These six characterized marine GH20 enzymes comprise Hex99 and Hex86 from *Pseudoalteromonas piscicida* (previously *Alteromonas* sp.) [21,35], Nag20A [36] and NagB [34] from the widespread *Aeromonas hydrophila*, chitobiase of *Vibrio harveyi* [37] and ExoI from *Vibrio furnissii* [33]. However, of these, only Hex99 from *P. piscicida* was examined for its ability to catalyze transglycosylation reactions [21]. 

Biochemical characteristics such as pH optimum (between pH 6.0‒7.0) and temperature optimum (37‒50 °C) of the six enzymes are rather similar. Moreover, based on *K*_M_ and *V*_max_ values, most of the enzymes have higher specific activity towards *p*-nitrophenyl-GlcNAc (*p*NPGlcNAc) compared to *p*-nitrophenyl-GalNAc (*p*NPGalNAc) [25,33,34,36].

It has not been possible to clearly distinguish between GH20 β-NAHAs from water-living and terrestrial organisms or from bacterial and eukaryotic organisms based solely on different functional features of the enzymes. The biochemical characteristics of GH20 β-NAHAs vary considerably, as it has been reviewed recently by Zhang et al. [25]. For example, pH optima of GH20 enzymes range from pH 3.0 for Hex of *Streptomyces plicatus* to pH 8.0 for Hex1 (from a metagenomic library) [18,25,38]. Affinities as given by *K*_M_ values for *p*NPGlcNAc range from 53 µM for *Cf*Hex20 from *Cellulomonas fimi* [39] to 120 mM for BbhI of *Bifidobacterium bifidum* [40]. Murine cytosolic β-NAHA shows *K*_M_ = 0.25 mM on *p*NPGalNAc, which it preferred over *p*NPGlcNAc [41]. Similarly, human plasma and pig brain β-NAHAs have a lower *K*_M_ for *p*NPGalNAc of 0.17 mM and 0.2 mM, respectively [42,43]. Interestingly, salt-tolerant HJ5Nag from *Microbacterium* sp. has a high *V*_max_ towards *p*NPGlcNAc of 3097 µmol·mg^−1^·min^−1^ [44]. One of the highest *k*_cat_ and catalytic efficiency values reported towards *p*NPGlcNAc are for *Cf*Hex20 of *C. fimi* reaching 480 s^−1^ and 9000 mM^−1^·s^−1^, respectively [39]. Crystal structures are available for several terrestrial GH20 β-NAHAs, e.g., Hex1T from *Paenibacillus* sp. TS12 [45], StrH from *Streptococcus pneumoniae* [46], HexA from *Streptomyces coelicolor* [47], Hex from *S. plicatus* [38] and Am2301 from *Akkermansia muciniphila* [48], but not for any aquatic GH20 enzymes.

Here, the genome of *P. hydrolytica* S66^T^ encoding 113 predicted glycoside hydrolases [1,3] was mined for β-NAHAs potentially acting on GlcNAc-containing compounds, e.g., chitooligosaccharides, which are abundant in the marine environment. Two putative GH20 encoding genes were identified in the genome, and one of the corresponding enzymes, *Ph*Nah20A, was produced recombinantly, characterized biochemically and moreover shown to catalyze transglycosylation using the GH20 reaction intermediate NAG-oxazoline [2-methyl-(1,2-dideoxy-α-d-glucopyrano)-oxazoline] as donor and lactose as well as a series of monosaccharides as acceptors.

## 2. Results and Discussion

### 2.1. Identification of Putative β-NAHAs in *P. hydrolytica* and Organization of Vicinal Genomic Regions

The marine bacterium *P. hydrolytica* degrades effectively many different polysaccharides [2] and its genome exhibits potential for the degradation of chitin and chitooligosaccharides. *P. hydrolytica* was grown in marine mineral medium supplemented with a mixture of chitooligosaccharides (GlcNAc)_1–6_ as the sole carbon source, which were hydrolyzed to GlcNAc (Supplementary Information 1, Figure S1A,B). *P. hydrolytica*, however, did not hydrolyze α-chitin from crab shells used to supplement the marine mineral medium, as neither GlcNAc nor chitooligosaccharides appeared during the incubation (Figure S1C). β-NAHA activity from *P. hydrolytica* was detected by a hydrolysis of the chromogenic 5-bromo-4-chloro-3-indolyl *N*-acetyl-β-d-glucosaminide (X-GlcNAc) on a complex marine agar medium (Figure S1D). These results indicated that the bacterium produced at least one β-NAHA which was active towards chitooligosaccharides.

The draft genome sequence of *P. hydrolytica* [1], deposited on the RAST server (http://rast.nmpdr.org/), encodes two putative GH20 β-NAHAs (EC 3.2.1.52) based on automatic annotation. Both genes were found in contig 11 of the *P. hydrolytica* whole genome shotgun sequence (NCBI accession: NZ_LSNE01000003.1). The protein sequence identity between full-length *Ph*Nah20A (WP_068373836.1) and *Ph*Nah20B (WP_082768773.1) was 23%.

Top hits of protein BLAST, showing up to 54% to *Ph*Nah20A and up to 49% sequence identity to *Ph*Nah20B, were GH20 β-NAHAs or chitobiases from phylogenetically closely related marine and soil bacteria belonging mostly to the same order as *P. hydrolytica*—*Alteromonadales* (Table S1). None of these proteins, encoded by genes from *Paraglaciecola* or related bacteria (Table S1), had been recombinantly produced or characterized.

**Figure 1:**
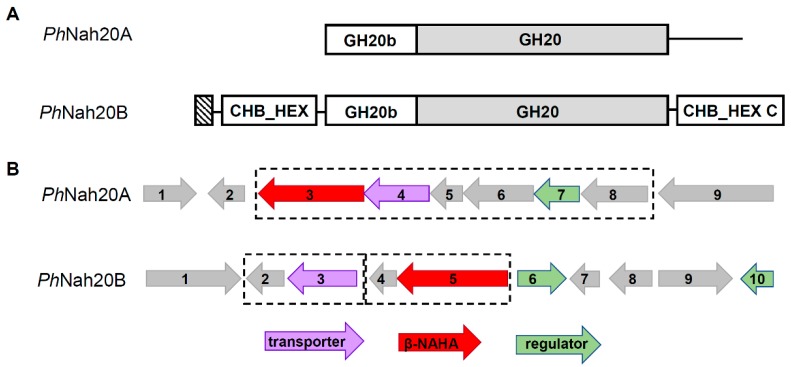
Schematic domain architecture of *P. hydrolytica Ph*Nah20A and *Ph*Nah20B (**A**) and of genomic regions flanking the two putative β-*N*-acetylhexosaminidases (β-NAHAs) (red arrows) (**B**). (**A**) GH20 catalytic domains are gray and the *N*-terminal signal peptide is striped. (**B**) Predicted protein functions are color coded. The information was retrieved from the National Center for Biotechnology Information (NCBI) database (NZ_LSNE01000003.1), Uniprot and Pfam databases. The regions flanking *Ph*Nah20A (3): 1, LemA family protein; 2, hypothetical protein; 4, sodium:solute symporter, putative SLC5sbd family protein; 5, RidA (reactive intermediate/imine deaminase A) family protein; 6, d-aminoacylase; 7, MurR/RpiR family transcriptional regulator; 8, amino acid deaminase; 9, sodium/proton-translocating pyrophosphatase. The regions flanking *Ph*Nah20B (5): 1, TonB-dependent receptor; 2, DUF1624 domain-containing protein, putative acyltransferase; 3, glucose/galactose MFS transporter; 4, hypothetical protein, putative BadF-type ATPase; 6, LacI family DNA-binding transcriptional regulator; 7, dCTP deaminase; 8, iron–sulfur cluster carrier protein ApbC; 9, methionine-tRNA ligase; 10, TetR/AcrR family transcriptional regulator. Predicted operons are in dashed frames.

The closest relatives of *Ph*Nah20A are GH20 β-NAHAs from *Bowmanella denitrificans* and *Lacimicrobium alkaliphilum* with 53%‒54% sequence identity (Table S1). *Ph*Nah20A contains two domains, the GH20 catalytic (β/α)_8_-barrel domain (Pfam: PF00728) and the N-terminal GH20b domain (also referred to as GH20 domain 2; Pfam: PF02838) of a predicted zincin-like fold similar to zinc-dependent metalloproteases [49] consisting of four antiparallel β-strands and an α-helix [27,50]. These two domains are typical for GH20 enzymes [50], and importantly they constitute an active and stable minimum functional unit of GH20 enzymes, thus requiring both a catalytic GH20 and a GH20b domain [50]. *Ph*Nah20A has no predicted signal peptide sequence and most probably is not secreted, whereas a 28 residues N-terminal signal peptide was predicted for the hypothetical *Ph*Nah20B (Figure 1A). Therefore, during the growth of *P. hydrolytica* on chitooligosaccharides, *Ph*Nah20B probably performs the initial degradation of these substrates. *Ph*Nah20B, in addition to the GH20b and GH20 domains, contains a putative carbohydrate binding domain of the CHB_HEX superfamily (Pfam: PF03173) having a predicted β-sandwich structure similar to cellulose binding domains in cellulases [51], and a C-terminal CHB_HEX_C domain (Pfam: PF03174) of unknown function resembling an immunoglobulin-like fold [50,51]. A similar four-domain architecture was seen in the crystal structure of a chitobiase from *S. marcescens* [51], and has only been reported for bacterial GH20 enzymes [50,51]. Based on its protein sequence identity and domain architecture, *Ph*Nah20B resembles a biochemically uncharacterized GH20 chitobiase from *Aliiglaciecola lipolytica* and β-NAHAs from other phylogenetically close marine bacteria (Table S1). It can be concluded that one of the reasons for low sequence identity, i.e., 23%, between two putative GH20 enzymes of *P. hydrolytica*, was the different domain architecture of *Ph*Nah20A and *Ph*Nah20B (Figure 1A), as *Ph*Nah20B has two additional domains besides the GH20 catalytic domain and an N-terminal GH20b domain. The identity between the two proteins remained low when only the predicted GH20b and GH20 domain sequences were compared, as some regions are not aligning between proteins (Supplementary Information 2).

Genomic regions flanking the two annotated *P. hydrolytica* β-NAHAs, *Ph*Nah20A and *Ph*Nah20B, were examined for the presence of operons (Figure 1B), but were found not to be organized similarly to the operon responsible for chitobiose-utilization in *Escherichia coli* [52]. Surrounding putative genes, however, encoded proteins potentially participating in the modification of acetylated compounds, the transporter function and transcription regulation (Figure 1B; Table S2). Notably, a predicted operon of six genes that harbors *Ph*Nah20A (Figure 1B) included a putative amino acid deaminase, d-aminoacylase and the RidA (reactive intermediate/imine deaminase A) family protein, possibly associated with the processing of acylated compounds or amino acids [53]. A two-gene operon was predicted to harbor *Ph*Nah20B and a putative ATPase (Figure 1B, Table S2). Thus, GH20 β-NAHAs genes of *P. hydrolytica* were not situated adjacent to genes encoding proteins directly coupled to β-NAHA activity, but flanking genes may be important for regulation or substrate transport.

### 2.2. Phylogenetic Analysis of *Ph*Nah20A and *Ph*Nah20B

**Figure 2:**
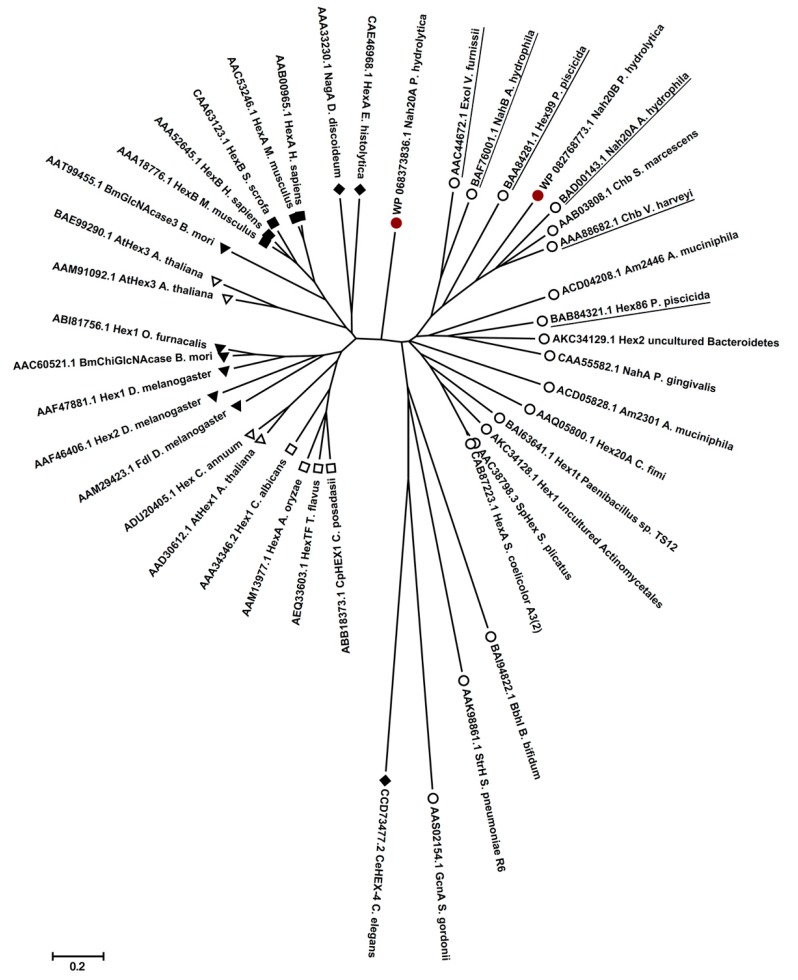
Schematic phylogenetic tree of *Ph*Nah20A, *Ph*Nah20B (both marked with red circles) and 41 biochemically characterized GH20 (EC 3.2.1.52) enzymes. Evolutionary analyzes were conducted, and the tree was composed and visualized using MEGA v 7.0.26 [55]. Protein sequences were aligned with Clustal Omega and the BLOSUM62 protein weight matrix was used. Evolutionary relationships were calculated using the Neighbor-Joining method. Evolutionary distances were computed using the Poisson correction method. All positions containing gaps and missing data were eliminated, and there was in total 292 positions in the final dataset. The tree is in scale with branch lengths in the same units as those of the evolutionary distances used to infer the phylogenetic tree. Bacterial (○), fungal (□), plant (Δ), insect (▲) and mammal (■) sequences. Amoebae and *C. elegans* sequences are marked with a filled diamond (♦). Characterized GH20 enzymes from marine organisms are underlined.

Sequences of *Ph*Nah20A, *Ph*Nah20B and 41 characterized GH20 enzymes were aligned (Supplementary Information 2). *Ph*Nah20A and *Ph*Nah20B shared a low sequence identity with the other GH20 enzymes (up to 34.1% for *Ph*Nah20A and 37.9% for *Ph*Nah20B) and only a few highly conserved regions were identified among these GH20 members (Supplementary Information 2). The closest homologs of *Ph*Nah20A were Hex2 of an uncultured *Bacteroidetes* (34.1% identity) and ExoI of the marine bacterium *V. furnissii* (33.1% identity). Remarkably, GH20 sequences from eukaryotes (human and mouse) were 31.3% and 30.9% identical and more similar to *Ph*Nah20A than most other included bacterial sequences. The *Ph*Nah20B sequence was most similar to chitobiases from *S. marcescens* (37.9% identity) and *V. harveyi* (36.4% identity). The evolutionary relationship illustrated by a radial phylogenetic tree (Figure 2; for bootstrap values see Figure S2) showed that bacterial GH20s segregate into three groups.

*Ph*Nah20B clustered with β-NAHAs from water-living bacteria from the phylogenetically close species such as *V. harveyi*, *P. piscicida* and *A. hydrophila*. However, *Ph*Nah20A did not cluster with characterized bacterial β-NAHAs but seems to represent a new distinct group of GH20 enzymes situated between predominantly water-living bacteria and the eukaryotes (Figure 2).

NagA of the slime mold *Dictyostelium discoideum* which clusters not far from *Ph*Nah20A (Figure 2), is a lysosomal enzyme that maintains the size of pseudoplasmodia [54], and shares 28.5% sequence identity with *Ph*Nah20A. According to the BLAST analysis, *Ph*Nah20A has higher sequence identity to biochemically uncharacterized β-NAHAs from phylogenetically close marine bacteria (Table S1). Additionally, protein sequences with 44–47% identity to *Ph*Nah20A were found in compost, hydrothermal vent and marine sediment metagenomes (Table S1) highlighting unexplored resources harbouring a new group of β-NAHAs.

According to the literature, substrate specificities and biochemical features (e.g., pH and temperature optima) are reported for 41 β-NAHAs of GH20 [4,32] mostly from terrestrial organisms. The few enzymes being from marine bacteria comprise ExoI and chitobiase from *Vibrio* sp. [33,37], Hex99 and Hex86 from *P. piscicida* [21,35] and Nag20A and NagB from *A. hydrophila* [34,36]. The limited knowledge on GH20 from marine organisms motivated the present characterisation of β-NAHA from *P. hydrolytica* S66^T^.

### 2.3. Cloning and Production of β-NAHA

**Figure 3:**
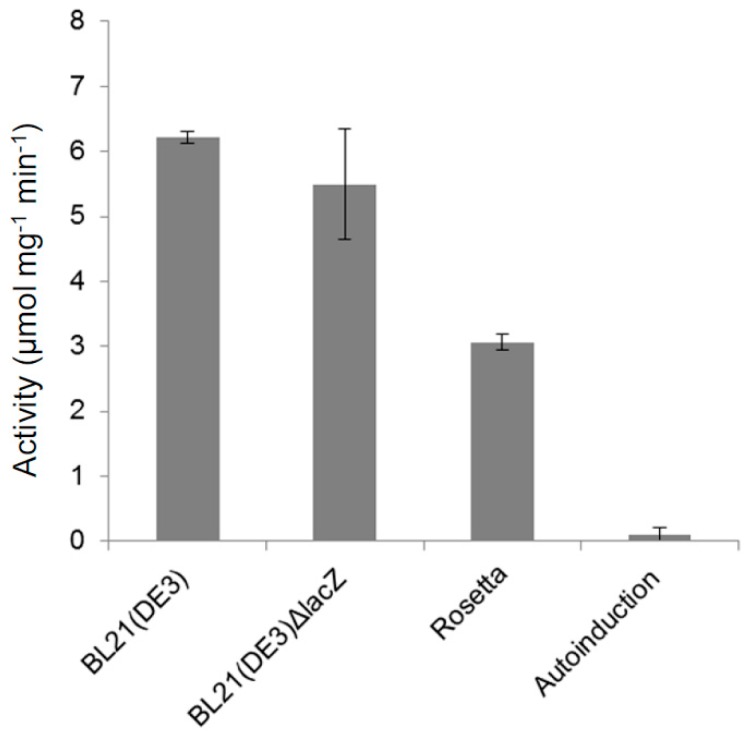
β-NAHA activity in µmol per min per mg of total protein in the lysates of three different *Escherichia coli* (*E. coli*) strains harboring *Ph*Nah20A grown in lysogeny broth (LB) induced by isopropyl thio-β-d-galactoside (IPTG) or in the auto-induction medium ZYM-5052 [56] (30 h). *E. coli* BL21(DE3) transformants carrying the full-length *Ph*Nah20A gene in an pURI3TEV expression vector were used in the auto-induction experiment. Values are given as the average of three independent experiments and the standard deviation (SD) is shown.

From the two candidate β-NAHA genes (Figure 1A), only recombinant *Ph*Nah20A was successfully produced in *E. coli* (Figure 3). *Ph*Nah20B cloned without the N-terminal signal peptide (Figure S3) was not obtained despite expression attempts in three *E. coli* strains [BL21(DE3), BL21(DE3)ΔlacZ and Rosetta], using different induction methods: isopropyl thio-β-d-galactoside (IPTG)-induction in lysogeny broth (LB) or auto-induction. *Ph*Nah20B was not found in the insoluble fraction by analyzing whole cells from IPTG-induced cultures (Figure S4). The yield of *Ph*Nah20A was modest, probably due to a low expression level. Using different strains and induction strategies resulted in the highest β-NAHA activity of 6 µmol *p*-nitrophenol released per min and per mg protein in the *E. coli* cell lysate for IPTG-induced BL21(DE3) transformants in LB medium (Figure 3).

Previously, an increased expression of GH20 β-NAHAs from a metagenome [18] was achieved in *E. coli* strains BL21(DE3), Turner, C41 or C43 grown in an auto-induction medium ZYM-5052 [56], but this medium gave a very low yield of *Ph*Nah20A (Figure 3) and failed to lead to *Ph*Nah20B production.

Up to 2 mg of *Ph*Nah20A was purified in two chromatographic steps from one liter of *E. coli* BL21(DE3) culture (see Section 3.4). Expression of truncated *Ph*Nah20A and *Ph*Nah20B, containing only the catalytic and not the GH20b domain (see Figure S3), did not result in protein production which is in agreement with previous findings that GH20b is essential for enzyme production and activity [50]. Attempts to produce *Ph*Nah20B without the CHB_HEX domains (Figure S3) also gave no detected protein or β-NAHA activity.

### 2.4. Characterization of *Ph*Nah20A

#### 2.4.1. Enzyme Stability

**Figure 4:**
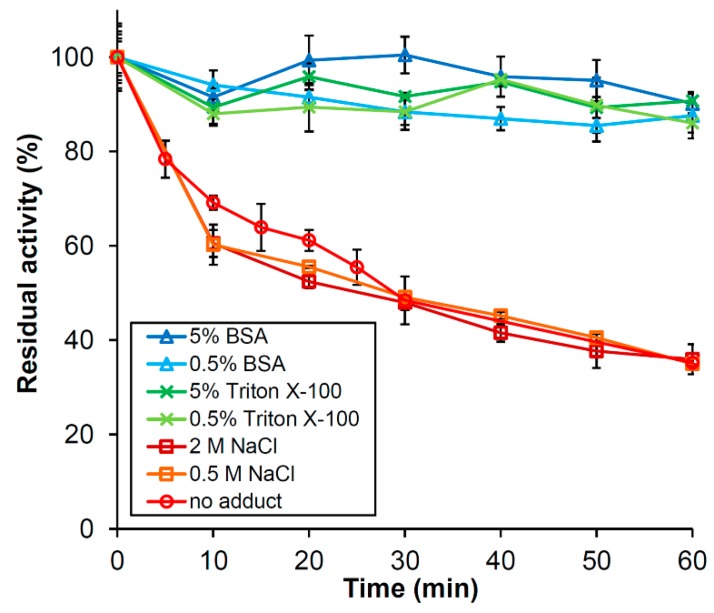
Effect of bovine serum albumin (BSA), Triton X-100, and NaCl on the stability of 5 µg·mL^−1^ *Ph*Nah20A on ice. The retained activity was measured at 37 °C in McIlvaine buffer, pH 6.0, using 2 mM *p*NPGlcNAc as the substrate. *Ph*Nah20A, BSA, NaCl and Triton X-100 were further 10 times diluted in the activity assay done at 0.5 µg·mL^−1^ *Ph*Nah20A, 0.05% or 0.5% BSA, 0.05% or 0.5% Triton X-100, 0.05 or 0.2 M NaCl.

**Figure 5:**
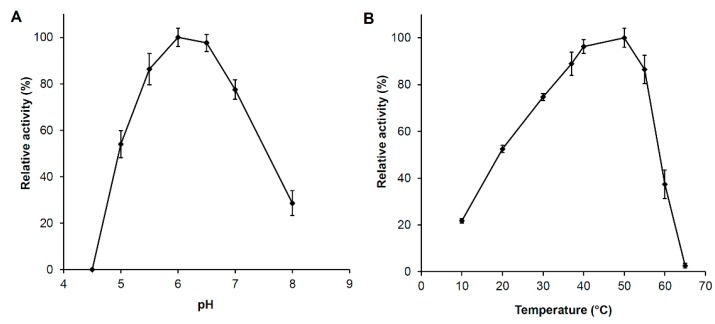
*Ph*Nah20A pH and temperature optima. The effect of pH (**A**) at 37 °C and the temperature at pH 6.0 (**B**) on initial rates of the hydrolysis of 2 mM *p*NPGlcNAc are both expressed as relative activity (%) from optimal activity values.

The activity of *Ph*Nah20A decreased immediately after dilution to the low concentration of 5 µg·mL^−1^, even when kept on ice (Figure 4). By contrast, 1 mg·mL^−1^ *Ph*Nah20A retained activity at least four months at 4 °C in 50 mM sodium phosphate pH 7.0, 0.3 M NaCl and 0.02% NaN_3_. The presence of 0.5% BSA or 0.5% Triton X-100 efficiently stabilized *Ph*Nah20A at 5 µg·mL^−1^ and pH 6.0 (see Figure 5A), whereas 0.5 and 2 M NaCl had no effect (Figure 4). This behavior and the absence of a signal peptide suggest *Ph*Nah20A is an intracellular enzyme. Without a stabilizing agent, 5 µg·mL^−1^ *Ph*Nah20A was completely inactivated within 5 min at 50 °C, while 50% and 3% activity were retained after 20 min and 4 h, respectively, in 0.5% BSA (Figure S5), and activity was fully retained after 4 d at 37 °C. β-NAHAs from *E. coli* [57], *Prunus serotina* [58], *Bos taurus* [59], *Hordeum vulgare* [60] and *Streptomyces plicatus* [61] were similarly found to lose activity by dilution. BSA has been identified as an activating compound to some β-NAHAs, e.g., from *Mus musculus* [41] and human plasma [42]. Notably, Hex, the commercial *S. plicatus* β-NAHA, is produced as a fusion with maltose-binding protein to secure stability and the Hex reaction mixture contained 0.3% of BSA to maintain activity [38].

#### 2.4.2. pH and Temperature Optima

*Ph*Nah20A was most active at pH 5.0–7.5 with a maximum around pH 6.0 (Figure 5A) and a temperature optimum at 50 °C (Figure 5B). The pH optimum of *Ph*Nah20A is highly similar to numerous characterized GH20 β-NAHAs [25], e.g., from *Microbacterium* sp. [44], *Paenibacillus* sp. [45] and *A. hydrophila* [34]. Some fungal β-NAHAs have more acidic pH optima (pH 4–5) [62,63,64]. Similarly high temperature optima as for *Ph*Nah20A were found for chitinases from *Salinivibrio costicola* [65], β-NAHAs from *Serratia marcescens* [66], *A. hydrophila* [36] as well as *Penicillium oxalicum* [27]. *Ph*Nah20A, however, when diluted in buffer lost activity completely within 5 min at 50 °C in the absence of stabilizers (Figure S5), emphasizing the importance of an environment with high protein concentration for the stability of *Ph*Nah20A. The optimal growth temperature of *P. hydrolytica* was 20–25 °C [2], but the temperature optimum for *Ph*Nah20A activity was much higher, which is a common phenomenon reported for other bacterial GH20 enzymes [27,66].

#### 2.4.3. Substrate Specificity and Kinetic Parameters of *Ph*Nah20A

*Ph*Nah20A hydrolyzed *N*,*N*′-diacetylchitobiose [chitobiose, (GlcNAc)_2_, β-GlcNAc-1,4-GlcNAc] and lacto-*N*-triose II (β-GlcNAc-1,3-β-Gal-1,4-Glc, LNT2) with the release of GlcNAc. Chitobiose was a poor substrate and 1 U·mL^−1^ (11.6 µg·mL^−1^) *Ph*Nah20A converted only 25% of 200 mM chitobiose in 20 h at pH 6.0 as analyzed by high-performance anion exchange chromatography with pulsed amperometric detector (HPAEC-PAD). Similarly, only trace amounts of GlcNAc were released from chitobiose by the GH20 BbhI from *B. bifidum* [40]. The action on LNT2 motivated assaying for transglycosylation activity (see Section 2.5), i.e., the ability to catalyze the reverse reaction of hydrolysis and in particular to produce HMOs, as described for the BbhI from *B. bifidum* [15,40].

Kinetic parameters for *Ph*Nah20A hydrolyzing *p*NPGlcNAc and *p*NPGalNAc (Table 1) were very similar, *k*_cat_ being slightly higher on *p*NPGalNAc. This identified *Ph*Nah20A as an *N*-acetylhexosaminidase rather than either an *N*-acetylglucosaminidase or an *N*-acetylgalactosaminidase. Most β-NAHAs, especially bacterial GH20 enzymes, prefer *p*NPGlcNAc (Table 1) and are referred to as *N*-acetylglucosaminidases. For instance, *S. marcescens* β-NAHA showed only 28.1% activity on *p*NPGalNAc compared to *p*NPGlcNAc [66]. Similarly, HexA from the ameba *E. histolytica* had 38% activity on *p*NPGalNAc compared to *p*NPGlcNAc [67]. Nag20A from *A. hydrophila* had very similar *K*_M_ for *p*NPGlcNAc and *p*NPGalNAc, but *V*_max_ for *p*NPGalNAc was only 13% of *V*_max_ for *p*NPGlcNAc [36]. Nag20B, also from *A. hydrophila*, showed about 20 times higher *K*_M_ for *p*NPGlcNAc and *p*NPGalNAc [34] than *Ph*Nah20A. Other differences include *V. furnissii* ExoI showing 3.6 times lower *K*_M_ towards *p*NPGlcNAc than *p*NPGalNAc [33]. Similarly, Hex1 and Hex2 from a metagenomic library showed very poor activity for *p*NPGalNAc [18]. On the other hand, β-NAHAs of human and mouse prefer *p*NPGalNAc as a substrate over *p*NPGlcNAc and have a high affinity towards it (*K*_M_ of 0.17 and 0.25 mM, respectively) [41,42]. Another eukaryotic β-NAHA from *D. discoideum* showed equal affinity (*K*_M_ of 1.5 mM) for both substrates [68], thus resembling more *Ph*Nah20A and some other bacterial enzymes (Table 1). Interestingly, BbhI had very high *K*_M_ of 120 mM for *p*NPGlcNAc (Table 1), but much lower *K*_M_ of 0.36 mM for LNT2 [40].

### 2.5. Transglycosylation by *PhNah20A*

**Figure 6:**
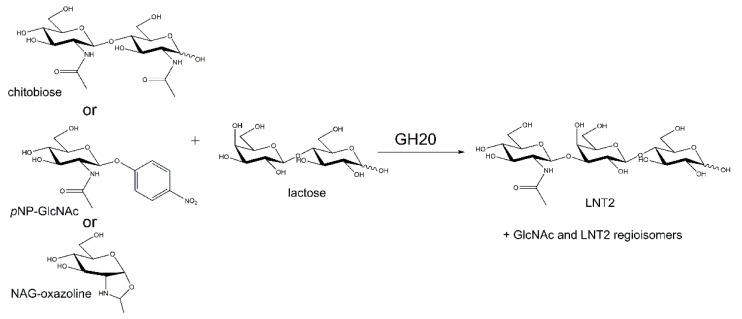
Scheme of transglycosylation reactions catalyzed by GH20 enzymes showing three different possible donors and lactose as an acceptor.

**Figure 7:**
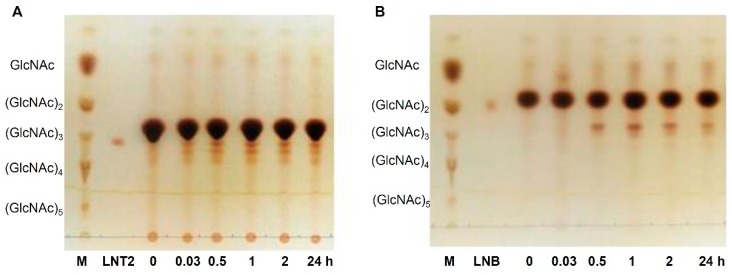
Time course of transglycosylation by *Ph*Nah20A (10 U·mL^−1^) with 100 mM NAG-oxazoline as donor and either 200 mM lactose (**A**) or d-galactose (**B**) as acceptor (see Section 3.8 for details). Chitooligosaccharides (M), lacto-*N*-triose II (LNT2) and lacto-*N*-biose (LNB) were used as references.

There are a few reports on LNT2 formation by GH20 catalyzed transglycosylation with (GlcNAc)_2_ or *p*NPGlcNAc as donors and lactose as the acceptor [15,18,64] (see Figure 6). Hydrolysis of LNT2 by *Ph*Nah20A, an HMO core structure [70], warranted the investigation of the transglycosylation with (GlcNAc)_2_ and the GH20 reaction intermediate NAG-oxazoline [2-methyl-(1,2-dideoxy-α-d-glucopyrano)-oxazoline] [71] as a donor and lactose as an acceptor (Figure 7A). We here also demonstrated transglycosylation by the commercial GH20 *N*-acetylglucosaminidase from *S. plicatus* (*Sp*Hex) [38] (see Figure S6), which has not been previously reported. Notably, the protein sequence identity between *Sp*Hex and a bacterial transglycosylating enzyme Hex1 isolated from a metagenome [18] was as high as 53.6%.

Transglycosylation by β-NAHAs has been rarely investigated, and in one case there is a report on a bacterial GH20 enzyme for which no transglycosylation was detected [72], indicating that not all GH20 enzymes have the ability to transglycosylate. A GH20 chitobiase Hex99 from the *Alteromonas* sp. strain O-7 (currently classified as *P. piscicida*) of the order *Alteromonadales* formed β-GlcNAc-1,6-GlcNAc from (GlcNAc)_2_ by transglycosylation. It is to date the only marine GH20 enzyme reported to produce GlcNAc-containing oligosaccharides [21]. Notably, *P. piscicida* belongs to the same bacterial order as *P. hydrolytica*. Hex99 has a unique substrate specificity, as it hydrolyzed only chitobiose and *p*NP(GlcNAc)_2_, but neither other chitooligosaccharides nor *p*NPGlcNAc.

**Figure 8:**
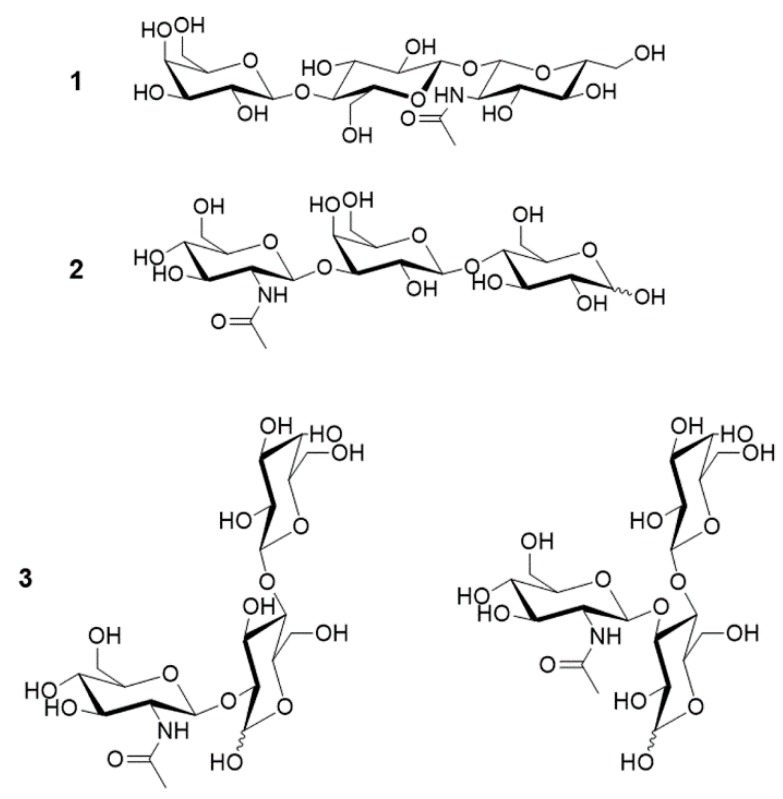
Structures of transglycosylation products determined by nuclear magnetic resonance (NMR). The three detected regioisomers are β-Gal-1,4-β-Glc-1,1-β-GlcNAc (**1**), β-GlcNAc-1,3-β-Gal-1,4-Glc, LNT2 (**2**) and β-Gal-1,4-(β-GlcNAc)-1,*x*-Glc (**3**), (*x* = 2, 3). Both possible structures of **3** are shown.

*Ph*Nah20A transglycosylated lactose with NAG-oxazoline as the donor (Figure 7A), resulting in three trisaccharides (Figure 8). **2**, purified by gel permeation chromatography (GPC) (Figure S7) migrated similarly to LNT2 in thin-layer chromatography (TLC), and nuclear magnetic resonance (NMR) spectroscopy confirmed the product structure (Figure S9). **1** was determined to be a non-reducing trisaccharide, β-Gal-1,4-β-Glc-1,1-β-GlcNAc (Figure 8 and Figure S8; Tables S3 and S4), once reported as a transglycosylation product of a β-NAHA from *Aspergillus flavofurcatis* CCF 3061 [73]. For full NMR assignment as well as all measurable ^3^*J*_H,H_ coupling constants of **1**, see Tables S3 and S4. The 1,1-linkage was supported by heteronuclear multiple-bond correlation spectroscopy (HMBC) and rotating frame nuclear Overhauser effect spectroscopy (ROESY) correlations between the two anomeric positions as well as by lack of a reducing end. Lastly, the β-configuration was determined of the anomeric positions using the ^3^*J*_H,H_ coupling constants between the anomeric proton and the neighboring proton (Table S4). A third trisaccharide (**3**) was detected, but not fully characterized due to low abundance. Based on chemical shifts of **3** (Figure S10), however, it seemed unlikely that the galactose moiety in lactose acted as an acceptor, as none of the corresponding chemical shifts were affected. Consequently, most probably the glucose moiety was the acceptor. As O6 was determined to be unsubstituted and glucose was the reducing end residue, therefore either β-Gal-(β-GlcNAc)-1,2-Glc or β-Gal-(β-GlcNAc)-1,3-Glc was produced (Figure S10).

Several examples exist in Nature of β-Gal-(β-GlcNAc)-1,2-Glc and β-GlcNAc-1,3-Glc being part of polysaccharide backbones, such as the *O*-antigens (*O*-polysaccharides) of lipopolysaccharides from Gram-negative bacteria, i.e., *Proteus* sp., *Hafnia alvei* and *Citrobacter werkmanii* [74,75,76].

The overall transglycosylation yield for trisaccharides was estimated from the high-performance anion exchange chromatography with pulsed amperometric detector (HPAEC-PAD) chromatogram to 3.8% obtained with 200 mM acceptor and 100 mM donor. Since other trisaccharides were formed, no further optimization of transglycosylation conditions were pursued, even though LNT2 was the major product. Notably, the three trisaccharides were not completely separated by gel permeation chromatography (GPC) (Figure S7), but thin-layer chromatography (TLC) and HPAEC-PAD analysis (Figures S7 and S11) showed products consistent with trisaccharides **1** and **3** (Figure 8).

The acceptor specificity of *Ph*Nah20A was explored using d-galactose, d-glucose, 2-deoxy-d-glucose or l-fucose as an acceptor and NAG-oxazoline as a donor. These monosaccharides all proved to be transglycosylated (Figure 7B and Figure S12) with the similar velocity and transglycosylation products being detected in the most cases already after 0.03 h (2 min) incubation. Therefore, 2 h incubation was sufficient to assess the transglycosylation ability of *Ph*Nah20A (Figure 7 and Figure S12). *Ph*Nah20A thus showed unusual promiscuity towards acceptor molecules, but due to the low yields and formation of several products as seen by TLC (Figure 7, Figure S12), purification and NMR analysis were not pursued. Remarkably, however, the ability to transglycosylate a wide range of acceptors has very rarely been reported for GH20 enzymes [18] and perhaps is associated with the marine origin and the unique phylogenetic relation of *Ph*Nah20A. *S. marcescens* Chb (see Section 2.2) is able to transglycosylate several alcohols, albeit sugar alcohols were not effective acceptors [66]. Some bacterial and fungal β-NAHAs can use lactose as their acceptor [15,18,64], and two Hex enzymes from uncultured bacteria were reported to transfer GlcNAc to d-glucose, d-galactose, sucrose and maltose [18].

## 3. Materials and Methods

### 3.1. Materials

LNT2 was purchased from Elicityl Oligotech (Crolles, France). Lactose, *p*NPGalNAc and 5-bromo-4-chloro-3-indolyl *N*-acetyl-β-d-glucosaminide (X-GlcNAc) were from Carbosynth (San Diego, CA, USA), *N*,*N*′-diacetylchitobiose [(GlcNAc)_2_] from Omicron Biochemicals (South Bend, IN, USA), and *N*,*N*′,*N*″-triacetylchitotriose [(GlcNAc)_3_] and *p*NPGlcNAc from Megazyme (Bray, Co. Wicklow, Ireland). A mixture of chitooligosaccharides, (GlcNAc)_1–6_, was from Koyo Chemicals (Osaka, Japan). All other chemicals were purchased from Sigma-Aldrich (Merck, Darmstadt, Germany) and used without further purification. *S. plicatus* β-NAHA in fusion with maltose-binding protein was purchased from New England Biolabs (Ipswich, MA, USA).

### 3.2. Bacterial Strains and Media

*Paraglaciecola hydrolytica* (type strain S66^T^) [1,2] was grown at 23 °C in Difco Marine Broth 2216 (BD, Franklin Lakes, NJ, USA) or on Marine Broth supplemented with 15 g·L^−1^ agar. X-GlcNAc was added to the marine agar medium to 20 mg·L^−1^. Hydrolytic activity was assessed in 5 mL marine mineral medium [77] supplemented with chitooligosaccharides (5 g·L^−1^) or α-chitin (2 g·L^−1^) at 23 °C. *E. coli* DH5α was used for molecular cloning, *E. coli* BL21(DE3), BL21(DE3)ΔlacZ [78] and Rosetta (Novagen, Merck, Darmstadt, Germany) for gene expression and *E. coli* BL21(DE3) for recombinant protein production. *E. coli* was grown in Lysogeny Broth (LB; MoBio Laboratories, Carlsbad, CA, USA) or on LB agar plates at 37 °C. Media were supplemented with 100 mg·L^−1^ ampicillin for selection. Auto-induction medium ZYM-5052 was prepared as described [56]. Liquid cultures were aerated on a shaker (160 rpm).

### 3.3. Molecular Cloning and Plasmids

*P. hydrolytica* genomic DNA was purified using the Gentra Puregene Yeast/Bact kit B (Qiagen, Venlo, The Netherlands) and plasmid DNA was isolated using the GeneJET Plasmid Miniprep kit (Thermo Fisher Scientific, Waltham, MA, USA). DNA content was determined on NanoDrop Lite (Thermo Fisher Scientific, Waltham, MA, USA). Two putative *P. hydrolytica* β-NAHA-encoding genes were amplified from genomic DNA by Phusion high-fidelity polymerase (Thermo Fisher Scientific, Waltham, MA, USA) using specific primers (Table S5). Genes were cloned as full-length or truncated variants (see Figure S3) into the pURI3TEV vector by PCR cloning [79].

DNA sequencing (Eurofins Genomics, Ebersberg, Germany) verified that cloned sequences matched the sequences in the *P. hydrolytica* genome. Plasmids were transformed into *E. coli* DH5α or BL21(DE3) by electroporation.

### 3.4. Recombinant Protein Production and Purification

For initial expression analysis *E. coli* BL21(DE3) harboring *Ph*Nah20A in pURI3TEV grown in 20 mL LB medium at 37 °C until OD_600nm_ ≈ 0.5 was induced by 0.5 mM isopropyl thio-β-d-galactoside (IPTG), and incubated at 22 °C. Aliquots (10 µL) were mixed at 0, 4, 20 h with 4 µL SDS-PAGE sample buffer, heated (10 min, 80 °C) to lyse cells and denature proteins, centrifuged (12,000× *g*, 1 min, RT) and analyzed on pre-cast SDS-polyacrylamide gels according to the manufacturers’ instructions (NuPAGE, Thermo Fisher Scientific, Waltham, MA, USA) in an XCell SureLock mini-cell electrophoresis system (Thermo Fisher Scientific, USA). Gels were stained by Coomassie Brilliant Blue G-250. Cell lysates were prepared from cell pellets after IPTG-induction by suspension in 0.4 mL 50 mM sodium phosphate pH 7.0, added 0.4 mL BugBuster protein extraction reagent (Merck, Darmstadt, Germany), approx. 100 U Benzonase nuclease (Merck, Darmstadt, Germany), and centrifuged (12,000× *g*, 20 min, 4 °C).

For enzyme preparation *E. coli* BL21(DE3) harboring *Ph*Nah20A in pURI3TEV was grown in 1 L LB medium at 37 °C to OD_600nm_ ≈ 0.5, induced by 0.5 mM IPTG, and incubated (20 h, 22 °C). Cells collected by centrifugation (10,000× *g*, 15 min, 4 °C) were resuspended in 50 mL lysis buffer (50 mM sodium phosphate, pH 7.0, 0.3 M NaCl, 20 mM imidazole containing 250 U Benzonase nuclease), disrupted (Cell Pressure Homogenizer, Stansted, UK) and centrifuged to remove debris (25,000× *g*, 20 min, 4 °C). The supernatant was filtered (0.45 µm sterile polyvinylidene fluoride (PVDF) membrane filter, Millex-HV, Merck, Darmstadt, Germany) and *Ph*Nah20A purified by Ni^2+^-affinity chromatography (HisTrapHP, GE Healthcare, Uppsala, Sweden) followed by size-exclusion chromatography (HiLoad 16/60 Superdex 200 pg; ÄKTA Avant chromatography system, GE Healthcare, Uppsala, Sweden) in 50 mM sodium phosphate, pH 7.0, 0.3 M NaCl at a flow rate of 2 mL·min^−1^. Eluate was analyzed by SDS-PAGE and fractions containing *Ph*Nah20A were pooled, concentrated (Amicon ultra-15 30K centrifugal filter device, Merck, Darmstadt, Germany), and had added to them 0.02% Na-azide, and then were stored in the above-mentioned buffer at 4 °C. Protein concentration was determined by the Pierce Coomassie (Bradford) Protein Assay Kit (Thermo Fisher Scientific, Waltham, MA, USA) for cell lysates and NanoDrop Lite (Themo Fisher Scientific, Waltham, MA, USA) for purified protein using the calculated ε_280_ = 136,835 M^−1^·cm^−1^ (ExPasy server; https://web.expasy.org/protparam/). After spectrophotometric determination of the concentration of *Ph*Nah20A, bovine serum albumin (BSA) was added to 0.5% of final concentration for storage.

### 3.5. Activity Assays

*Ph*Nah20A activity was routinely determined on 2 mM *p*NPGlcNAc at 37 °C in two-fold diluted McIlvaine buffer pH 6.0 (63 mM Na_2_HPO_4_; 18 mM citric acid), containing 0.05% BSA. The reaction (total volume 500 µL) was performed in McIlvaine buffer, pH 6.0 (250 µl), 100 µl milliQ water and 100 µL of substrate was added. The reaction was initiated by adding 50 µL of *Ph*Nah20A (prepared immediately before use in McIlvaine buffer, pH 6.0, 0.5% BSA, and kept on ice) to the reaction mixture yielding a final concentration of 0.3–5 µg·mL^−1^. The reaction was stopped typically after 2–5 min by 250 µL 1 M Na_2_CO_3_ and the product was measured spectrophotometrically at 400 nm (Ultrospec 3100 pro UV/Visible spectrophotometer, GE Healthcare, Uppsala, Sweden) using *p*NP (ε_400_ = 18,000 M^−1^·cm^−1^) as the standard. One U of activity was defined as the amount of enzyme releasing 1 µmol *p*NP per min from 2 mM *p*NPGlcNAc. pH activity optimum was determined for *Ph*Nah20A in McIlvaine buffers (pH 4.0–8.0) at 37 °C towards 2 mM *p*NPGlcNAc and the temperature optimum was determined from the initial rates of *p*NP release at temperatures in the range 10–65 °C at pH 6.0.

To determine the hydrolysis by *Ph*Nah20A 200 mM (GlcNAc)_2_ was incubated with 1 U·mL^−1^ (11.6 µg·mL^−1^) or 5 mM LNT2 with 10 U·mL^−1^ (116 µg·mL^−1^) in 50 mM sodium phosphate, pH 6.0, 0.5% BSA, at 37 °C for 20 h. The release of GlcNAc was monitored by high-performance anion exchange chromatography with pulsed amperometric detector (HPAEC-PAD) for (GlcNAc)_2_ and by thin-layer chromatography (TLC) for LNT2 (see Section 3.9).

### 3.6. Kinetics

*Ph*Nah20A (final concentration 0.3–1.2 µg·mL^−1^) was added to initiate the hydrolysis of 0.05–2 mM *p*NPGlcNAc (six concentrations) and 0.1–2 mM *p*NPGalNAc (five concentrations) in 500 μL two-fold diluted McIlvaine buffer pH 6.0, 0.05% BSA at 37 °C. The reaction was stopped at suitable time points by the addition of 250 μL 1 M Na_2_CO_3_ and quantified spectrophotometrically as above. Initial rates calculated from *p*NP formation versus time were plotted against substrate concentration and fitted to the Michaelis-Menten equation using OriginPro 2015 (OriginLab, Northampton, MA, USA) to obtain *k*_cat_ and *K*_M_. The *k*_cat_/*K*_M_ values were either calculated or determined from rates of hydrolysis at low substrate concentration.

### 3.7. Synthesis of NAG-Oxazoline

NAG-oxazoline [2-methyl-(1,2-dideoxy-α-d-glucopyrano)-oxazoline] was synthesized and purified as described previously [71]. Briefly, 2 g GlcNAc (9 mmol) was dissolved in 20 mL acetic anhydride, then we added 10 mL pyridine and stirred overnight at room temperature (RT). After extraction by dichloromethane (DCM) and successive washings (Na_2_CO_3_, H_2_O, H_2_SO_4_, H_2_O) the organic layer was dried and evaporated. Trimethylsilyl trifluoromethanesulfonate (0.8 mL) was added to 1.5 g peracetylated glucosamine dissolved in 1,2-dichloroethane and stirred at 50 °C until completion of the reaction (about 4 h). Trimethylamine was added (2 mL) followed by 50 mL DCM, washed with cold water, dried and evaporated. The product was purified by flash chromatography (cyclohexane: 1% triethylamine in ethyl acetate 100:0 to 40:60). Peracetylated oxazole (300 mg) in 10 mL anhydrous methanol at 0 °C was added 15 μL 5.3 M sodium methanolate in methanol and stirred at RT until the reaction was completed (about 3 h). The resulting NAG-oxazoline was dried and used without further purification.

### 3.8. Transglycosylation

Reaction mixtures for transglycosylation contained either 100 mM NAG-oxazoline (from 1 M stock in 50 mM sodium borate, pH 9.3) or 100 mM (GlcNAc)_2_ donor, and as acceptor 200 mM lactose, d-galactose, d-glucose, 2-deoxy-d-glucose or l-fucose; 1 or 10 U·mL^−1^ (11.6 or 116 µg·mL^−1^) *Ph*Nah20A or 10 U·mL^−1^ *S. plicatus* β-NAHA in 50 mM sodium phosphate, pH 8.0, 0.5% BSA, at 37 °C. The reaction volume was typically 20 µL for TLC analysis and 250 µL for product yield and structure determination. Slightly basic conditions were required as NAG-oxazoline is not stable at neutral or acidic pH [71]. Reactions were stopped at various time points by heating (5 min, 90 °C), cooled to RT and centrifuged (12,000× *g*, 1 min, 4 °C). Samples were diluted four- and 150-fold in milliQ water for TLC and HPAEC-PAD (see Section 3.9), respectively. For the reaction mixtures for the analysis of transglycosylation products after gel permeation chromatography (GPC), containing 10 U·mL^−1^ *Ph*Nah20A, 100 mM NAG-oxazoline and 200 mM lactose in 50 mM sodium phosphate pH 8.0, 0.5% BSA were incubated 2 h at 37 °C followed by heating (5 min, 90 °C). To the sample was added three volumes of sterile milliQ water, and the enzyme was removed (Amicon Ultra 0.5 mL centrifugal device, Mw cut-off 30 kDa; Merck, Darmstadt, Germany) followed by filtration (0.45 µm filters; Millex-HV, Merck, Darmstadt, Germany) prior to GPC.

### 3.9. Chromatographic Methods

Reaction mixtures containing 15–30 µg carbohydrate were spotted onto TLC plates (Silica Gel 60 F254 plates; Merck, Darmstadt, Germany) developed twice in chloroform:acetic acid:water (6:7:1; *v*:*v*:*v*) [80,81] or n-butanol: ethanol: water (5:3:2; *v*:*v*:*v*) [82]. Carbohydrates were visualized with orcinol (0.5% 5-methyl resorcinol and 10% H_2_SO_4_ in ethanol) or aniline dye (1.2% aniline hydrochloride and 1.2% diphenylamine in acidic methanol).

Oligosaccharides were also separated by HPAEC-PAD at 22 °C (Dionex CarboPac PA1 column, 250 × 4 mm with 50 × 4 mm Guard, Thermo Fisher Scientific, Waltham, MA, USA) using an ICS-5000 system (Thermo Fisher Scientific, Waltham, MA, USA) equipped with AS autosampler and pulsed amperometric detector (carbohydrate four-potential waveform, sampling rate 2 Hz) with a gold electrode (Au) and an Ag/AgCl reference electrode.

The elution was done with (A) water; (B) 1 M NaOH; (C) 200 mM NaOH + 800 mM NaOAc isocratically using 7.5% B in A (25 min) followed by 100% C (1 min) and column re-equilibration (9 min) at 7.5% B in A at 1.0 mL·min^−1^. Oligosaccharides in water (10 µL) containing 9 µM l-fucose as standard were injected by autosampler kept at 5 °C. LNT2, glucose, galactose, lactose, GlcNAc, (GlcNAc)_2_ and chitooligosaccharides were used as standards for calibration. Reaction mixtures (0.5 mL) containing approximately 10 mg oligosaccharides were separated by GPC (Bio-Gel P-2, Bio-Rad Laboratories, Hercules, CA, USA; 16 × 900 mm XK16/100 mounted on an ÄKTAprime plus chromatography system, GE Healthcare, Sweden), eluted by degassed milliQ water at flow rate of 0.1 mL·min^−1^ at RT and pressure limit set to 0.3 MPa. Reducing sugar in collected fractions (2 mL) were quantified by the Nelson-Somogyi method [83] using glucose and GlcNAc as standards. Fractions containing trisaccharides were dried (SpeedVac, Thermo Fisher Scientific, Waltham, MA, USA) at 50 °C, dissolved in 50 µL milliQ water and subjected to TLC for the preliminary identification of transglycosylation products. For NMR analysis, identical trisaccharide-containing fractions from two GPC runs were pooled, dried (SpeedVac) and dissolved in 0.5 mL D_2_O (Sigma-Aldrich, USA). Each fraction contained a major component and trace amounts of one or two other products.

### 3.10. Nuclear Magnetic Resonance (NMR)

All NMR spectra were recorded on an 800 MHz Bruker Avance III (799.75 MHz for ^1^H and 201.10 MHz for ^13^C) equipped with a 5 mm TCI cryoprobe. Acetone was used as internal reference (2.22 ppm and 30.89 ppm for ^1^H and ^13^C, respectively). The following experiments were used for the structure elucidation: ^1^H with presaturation, double quantum filtered correlation spectroscopy (DQF-COSY), rotating frame nuclear Overhauser effect spectroscopy (ROESY), heteronuclear single-quantum correlation spectroscopy (HSQC), heteronuclear single-quantum correlation spectroscopy-total correlation spectroscopy (HSQC-TOCSY) and heteronuclear multiple-bond correlation spectroscopy (HMBC) all performed using standard Bruker pulse sequences. LNT2 and lactose were used as reference compounds. Structural elucidation was carried out by first identifying all ^1^H and corresponding ^13^C chemical shifts using ^1^H with presaturation and HSQC. Subsequently, the different signals belonging to each position in each monosaccharide were determined, primarily using DQF-COSY and HSQC-TOCSY, and finally the connections between the monosaccharides were determined using HMBC and ROESY, as well as comparing chemical shifts to reference compounds.

### 3.11. In Silico Methods

The draft genome sequence of *P. hydrolytica* S66^T^ [1] annotated on the RAST server (http://rast.nmpdr.org/) [84] was mined on 20 March 2016, to identify putative β-NAHAs. Visualization of the RAST-annotated proteins was done on the SEED Viewer v 2.0 (www.theSEED.org).

Protein sequences of characterized β-NAHAs were retrieved from UniprotKB (https://www.uniprot.org/) on 10 February 2019. Nucleotide BLAST and protein BLAST tools (https://blast.ncbi.nlm.nih.gov/Blast.cgi) were used in 10 February 2019 and 3 January 2020 for identity analysis of nucleotide and protein sequences, respectively. Multiple sequence alignments were carried out with Clustal Omega v 2.1 (https://www.ebi.ac.uk/Tools/msa/clustalo/) and visualized by BioEdit v 7.0.5.3 (https://www.softpedia.com/get/Science-CAD/BioEdit.shtml).

The phylogenetic tree was constructed and visualized using MEGA v 7.0.26 (https://megasoftware.net/) [55].

*N*-terminal signal peptide prediction was done by SignalP v 4.1 with sensitive default cut-off values (http://www.cbs.dtu.dk/services/SignalP/) [85]. Promoter locations were predicted by SoftBerry tool BPROM (http://www.softberry.com/berry.phtml?topic=bprom&group=programs&subgroup=gfindb).

## 4. Conclusions

The genome of the marine bacterium *P. hydrolytica* S66^T^ encodes two putative GH20 β-*N*-acetylhexosaminidase (EC 3.2.1.52) having protein sequences that differed remarkably from earlier characterized β-NAHAs (≤30% identity). *Ph*Nah20A was positioned on a phylogenetic tree between β-NAHAs of water-associated bacteria, i.e., *Vibrio furnissii* and *Aeromonas hydrophila*, and unicellular eukaryotes (amobae). *Ph*Nah20A, produced in *E. coli*, was unstable if diluted, but was stabilized by BSA or Triton X-100. *Ph*Nah20A is a genuine β-NAHA with essentially the same catalytic efficiency for *p*NPGlcNAc and *p*NPGalNAc, and thus differs from most of the previously studied bacterial β-NAHAs, which prefer *p*NPGlcNAc as a substrate while some eukaryotic GH20 prefer *p*NPGalNAc. *Ph*Nah20A also hydrolyzed LNT2, a core structure of human milk oligosaccharides, and showed biosynthetic activity (transglycosylation) which is a poorly studied aspect of GH20 β-NAHAs, especially from eukaryotes and water-living prokaryotes. *Ph*Nah20A was able to form LTN2 by transglycosylation using NAG-oxazoline as a donor and lactose as an acceptor, LNT2, β-Gal-1,4-β-Glc-1,1-β-GlcNAc and β-Gal-1,4-(β-GlcNAc)-1,2/3-Glc being identified by NMR as main transglycosylation products. Several monosaccharides were also recognized as acceptors by *Ph*Nah20A. To date, based on pH and temperature optima, kinetic parameters or stability characteristics alone, no clear distinction can be made between eukaryotic versus prokaryotic or terrestrial versus aquatic GH20 β-NAHAs. However, this may be due to the very limited number of characterized β-NAHAs of salt or fresh water origin. *Ph*Nah20A is the first characterized member of a distinct group of GH20 β-NAHAs located phylogenetically between eukaryotic and prokaryotic enzymes.

## Figures and Tables

**Table 1:** Kinetic parameters of *Ph*Nah20A and β-NAHAs from the literature on *p*NPGlcNAc and *p*NPGalNAc. *Sm*—*S. marcescens*; *Ah*—*A. hydrophila*; *Bb*—*B. bifidum*; *Cf*—*C. fimi*; *Vf*—*V. furnissii*; *Eh*—*E. histolytica*; *Tr*—*Trichoderma reesei*.

Enzyme	Substrate	*K*_M_ (mM)	*V*_max_ (µmoL·mg^−1^·min^−1^)	*k*_cat_ (s^−1^)	*k*_cat_/*K*_M_ (mM^−1^·s^−1^)
*Ph*Nah20A	*p*NPGlcNAc	0.43 ± 0.07	93.7 ± 5.0	146.8	341
	*p*NPGalNAc	0.56 ± 0.11	123.0 ± 7.0	192.7	344
*Sm*Chb ^1^	*p*NPGlcNAc	56.7 ± 4.3	NI	111.0	1.95
*Ah*Nag20A ^2^	*p*NPGlcNAc	0.52	115	NI	NI
	*p*NPGalNAc	0.5	7.6	NI	NI
*Ah*NagB ^2^	*p*NPGlcNAc	8.57	25	NI	NI
	*p*NPGalNAc	11.1	11	NI	NI
*BbhI* of *Bb* ^3^	*p*NPGlcNAc	120.0 ± 0.2	NI	213	178
	*p*NPGalNAc	NA	NA	NA	NA
*Cf*Hex20 ^4^	*p*NPGlcNAc	0.053	NI	482.3	9090
	*p*NPGalNAc	0.066	NI	129.1	1950
*Vf*ExoI ^5^	*p*NPGlcNAc	0.09	270	NI	NI
	*p*NPGalNAc	0.33	130	NI	NI
Hex2 ^6^	*p*NPGlcNAc	0.48	NI	60.0 ± 1.7	NI
*Eh*HexA ^7^	*p*NPGlcNAc	0.1	3.8	NI	NI
*Tr*Nag1	*p*NPGlcNAc	69.4 ± 4.0	NI	NI	1023 ± 23

Data from ^1^ [69], ^2^ [36] and [34], ^3^ [40], ^4^ [39], ^5^ [33], ^6^ [18], ^7^ [67]. NI—not indicated; NA—no detected activity.

## Supplementary Materials

The following are available online at https://www.mdpi.com/1422-0067/21/2/417/s1. The following materials are available online: Supplementary Information 1 containing Figure S1. TLC analysis of growth media of *P. hydrolytica* (A, B, C) and growth phenotype on marine agar medium with X-GlcNAc (5-bromo-4-chloro-3-indolyl *N*-acetyl-β-d-glucosaminide) (D); Figure S2. Phylogenetic tree with bootstrap test (1000 replicates) of *Ph*Nah20A, *Ph*Nah20B (both marked with red circles) and 41 biochemically characterised GH20 (EC 3.2.1.52) enzymes; Figure S3. Schematic domain architecture of full-length and truncated variants of *Ph*Nah20A and *Ph*Nah20B; Figure S4. IPTG-induced *E. coli* transformants growing in LB analysed by SDS-PAGE; Figure S5. Inactivation of 5 µg·mL^−1^ *Ph*Nah20A at 50 °C and pH 6.0 in the presence of 0.5% BSA; Figure S6. Time course of transglycosylation by *S. plicatus* Hex (10 U·mL^−1^) with 100 mM NAG-oxazoline as donor and 200 mM lactose as acceptor; Figure S7. TLC of trisaccharide-containing fractions of the *Ph*Nah20A reaction (2 h; Figure 7A) separated by gel-permeation chromatography; Figure S8. HSCQ spectrum of the chromatographic fraction 50 (see Figure S7) containing over 80% of compound **1**; Figure S9. HSCQ spectrum of the chromatographic fraction 51 (see Figure S7) containing over 75% of 2 (LNT2); Figure S10. HSCQ spectrum of the chromatographic fraction 53 (see Figure S7) containing primarily 3; Figure S11. Extraction of HPAEC-PAD analysis of transglycosylation products by *Ph*Nah20A (10 U·mL^−1^) reacting 2 h with 100 mM NAG-oxazoline as donor and 200 mM lactose as acceptor (blue line); Figure S12. Time course of transglycosylation by *Ph*Nah20A (10 U·mL^−1^) with 100 mM NAG-oxazoline as donor and 200 mM D-glucose (A), 2-deoxy-d-glucose (B) or L-fucose (C) as acceptor; Table S1. BLAST analysis of putative β-NAHAs (EC 3.2.1.52) from *P. hydrolytica*. Table S2. Information on proteins flanking identified β-NAHAs (presented in Figure 1B); Table S3. NMR assignment of 1. The methyl of the GlcNAc acetyl group was at 2.090 ppm for ^1^H and 22.81 ppm for ^13^C and the carbonyl of the acetyl was at 176.06 ppm ^13^C; Table S4. ^3^H-H coupling constants for 1 measured through DQF-COSY; Table S5. PCR primers to isolate full-length β-NAHA encoding genes and indicated truncated variants. Underlined sequences are priming with pURI3-TEV expression vector; Supplementary Information 2 containing multiple sequence alignment.

## Abbreviations

BSA	bovine serum albumin
DCM	dichloromethane
GH	glycoside hydrolase
GlcNAc	*N*-acetylglucosamine
(GlcNAc)_2_	*N*,*N*’-diacetylchitobiose, chitobiose
GPC	gel permeation chromatography
HMOs	human milk oligosaccharides
HPAEC-PAD	high-performance anion exchange chromatography with pulsed amperometric detector
IPTG	isopropyl thio-β-d-galactoside
LNT2	lacto-*N*-triose II
NAG-oxazoline	2-methyl-(1,2-dideoxy-α-d-glucopyrano)-oxazoline
β-NAHA	β-*N*-acetylhexosaminidases
NCBI	National Center for Biotechnology Information
NMR	nuclear magnetic resonance
*p*NPGlcNAc	*p*-nitrophenyl-GlcNAc
*p*NPGalNAc	*p*-nitrophenyl-GalNAc
TLC	thin layer chromatography
X-GlcNAc	5-bromo-4-chloro-3-indolyl *N*-acetyl-β-d-glucosaminide
