# Supplementary material for: Identification and Characterization of a β-N-Acetylhexosaminidase with a Biosynthetic Activity from the Marine Bacterium Paraglaciecola hydrolytica S66T
Source: Int J Mol Sci. 2020 Jan 9;21(2):417. doi: 10.3390/ijms21020417 (PMC7014002; doi:10.3390/ijms21020417)
Supplement: Supplementary file 1 [file ijms-21-00417-s001.zip › ijms-670241-SI-to conversion/Supplementary Information 1 Visnapuu et al updated.pdf]

Supplementary Information 1

Identification and Characterization of a β-*N*-Acetylhexosaminidase with a Biosynthetic Activity from the Marine Bacterium *Paraglaciecola hydrolytica* S66^{T}

Triinu Visnapuu [1,2,*], David Teze [1], Christian Kjeldsen [3], Aleksander Lie [4,a], Jens Øllgaard Duus [3], Corinne André-Miral [5], Lars Haastrup Pedersen [4], Peter Stougaard [6,b] and Birte Svensson [1,*]

[1] Department of Biotechnology and Biomedicine, Technical University of Denmark, Søltofts Plads, building 224, DK-2800, Kgs. Lyngby, Denmark; triinu.visnapuu@ut.ee (T.V.); david.teze@gmail.com (D.T.); bis@bio.dtu.dk (B.S.)
[2] Institute of Molecular and Cell Biology, University of Tartu, Riia 23, 51010 Tartu, Estonia
[3] Department of Chemistry, Technical University of Denmark, Kemitorvet, building 207, DK-2800, Kgs. Lyngby, Denmark; chkje@kemi.dtu.dk (C.K.); jduus@kemi.dtu.dk (J.Ø.D.)
[4] Department of Chemistry and Bioscience, Aalborg University, Fredrik Bajers Vej 7, H, DK-9220, Aalborg, Denmark; lhp@bio.aau.dk (L.H.P.); al@kebony.com (A.L.)
[5] Université de Nantes, CNRS, Unité de Fonctionnalité et Ingénierie des Protéines (UFIP), UMR 6286, F-44000 Nantes, France; corinne.miral@univ-nantes.fr (C.A.-M.)
[6] Department of Plant and Environmental Sciences, University of Copenhagen, Thorvaldsensvej 40, DK-1871, Frederiksberg C, Denmark; pst@envs.au.dk (P.S.)
* Correspondence: triinu.visnapuu@ut.ee; Tel.: +372-737-5013 (T.V.); bis@bio.dtu.dk; Tel.: +45-4525-2740 (B.S.)

[a] Present affiliation: Kebony Norge AS, Havneveien 35, NO-3739, Skien, Norway

[b] Present affiliation: Department of Environmental Science - Environmental Microbiology and Circular Resource Flow, Aarhus University, Frederiksborgvej 399, building 7411, B2.12, DK-4000, Roskilde, Denmark

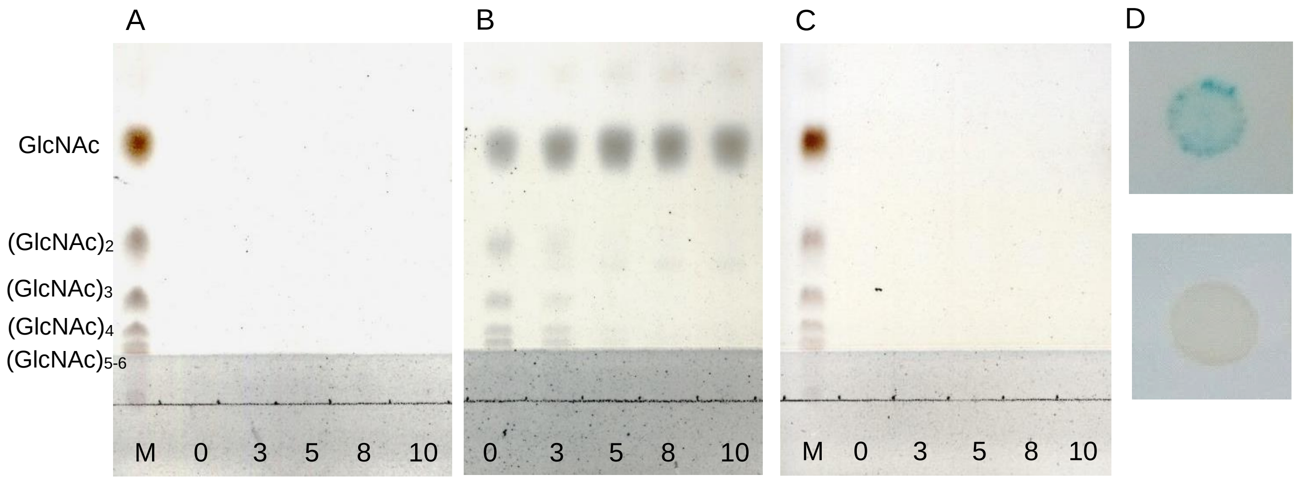

Fig S1. TLC analysis of growth media of *P. hydrolytica* (A, B, C) and growth phenotype on marine agar medium with X-GlcNAc (5-bromo-4-chloro-3-indolyl *N*-acetyl-β-D-glucosaminide) (D). The strain *P. hydrolytica* S66^T was growing up to 10 days (indicated on Figure S1) at 23 °C in marine mineral medium supplemented with 5 g L^{-1} chitooligosaccharides (B) or 2 g L^{-1} α-chitin (C) as a sole carbon source. The medium without added carbon source was inoculated with *P. hydrolytica* and used for comparison (A). M – marker, chitooligosaccharides (DP 1–6, from Koyo Chemicals). Culture supernatant (0.5 μL) was spotted on TLC plates, developed in chloroform: acetic acid: water (6:7:1, v:v:v). Sugars were visualized using aniline dye (1.2% aniline hydrochloride and 1.2% diphenylamine in acidic methanol). Panels A and B are from the same TLC plate. *P. hydrolytica* inoculated on marine agar medium supplemented with X-GlcNAc (5-bromo-4-chloro-3-indolyl *N*-acetyl-β-D-glucosaminide) (D, upper panel) was incubated 5 d at 23 °C. The phenotype of the bacterium on marine agar without supplement is shown for comparison (D, lower panel).

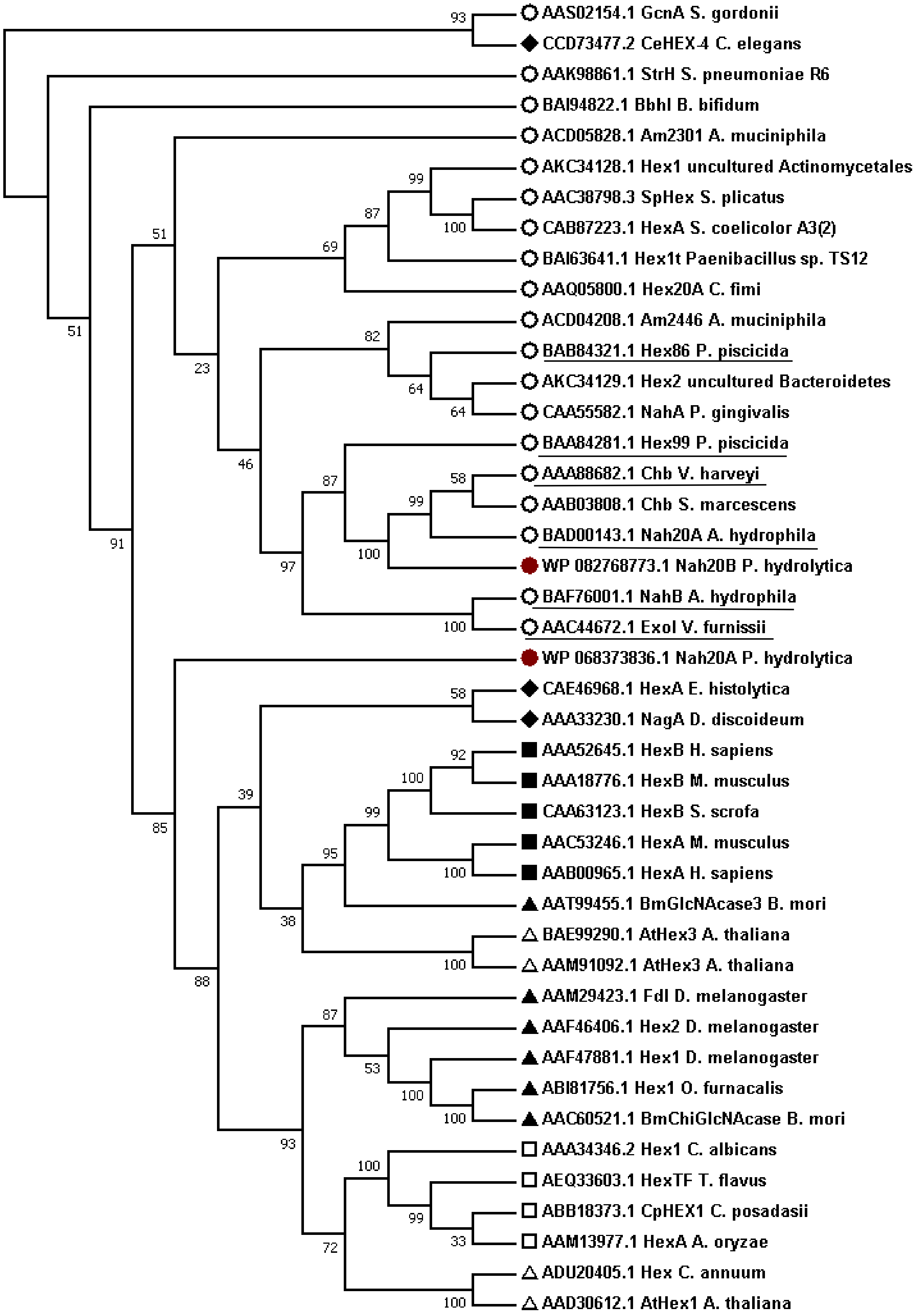
AAS02154.1 GcnA S. gordonii
CCD73477.2 CeHEX-4 C. elegans
AAK98861.1 StrH S. pneumoniae R6
BAI94822.1 BbhI B. bifidum
ACD05828.1 Am2301 A. muciniphila
AKC34128.1 Hex1 uncultured Actinomycetales
AAC38798.3 SpHex S. plicatus
CAB87223.1 HexA S. coelicolor A3(2)
BAI63641.1 Hex1t Paenibacillus sp. TS12
AAQ05800.1 Hex20A C. fimi
ACD04208.1 Am2446 A. muciniphila
BAB84321.1 Hex86 P. piscicida
AKC34129.1 Hex2 uncultured Bacteroidetes
CAA55582.1 NahA P. gingivalis
BAA84281.1 Hex99 P. piscicida
AAA88682.1 Chb V. harveyi
AAB03808.1 Chb S. marcescens
BAD00143.1 Nah20A A. hydrophila
WP 082768773.1 Nah20B P. hydrolytica
BAF76001.1 NahB A. hydrophila
AAC44672.1 ExoI V. furnissii
WP 068373836.1 Nah20A P. hydrolytica
CAE46968.1 HexA E. histolytica
AAA33230.1 NagA D. discoideum
AAA52645.1 HexB H. sapiens
AAA18776.1 HexB M. musculus
CAA63123.1 HexB S. scrofa
AAC53246.1 HexA M. musculus
AAB00965.1 HexA H. sapiens
AAT99455.1 BmGlcNAcase3 B. mori
BAE99290.1 AtHex3 A. thaliana
AAM91092.1 AtHex3 A. thaliana
AAM29423.1 Fdl D. melanogaster
AAF46406.1 Hex2 D. melanogaster
AAF47881.1 Hex1 D. melanogaster
ABI81756.1 Hex1 O. furnacalis
AAC60521.1 BmChiGlcNAcase B. mori
AAA34346.2 Hex1 C. albicans
AEQ33603.1 HexTF T. flavus
ABB18373.1 CpHEX1 C. posadasii
AAM13977.1 HexA A. oryzae
ADU20405.1 Hex C. annuum
AAD30612.1 AtHex1 A. thaliana

Fig S2. **Phylogenetic tree with bootstrap test (1000 replicates) of *Ph*Nah20A, *Ph*Nah20B (both marked with red circles) and 41 biochemically characterised GH20 (EC 3.2.1.52) enzymes.** Evolutionary analyses were conducted, and the tree was composed and visualised using MEGA7. Protein sequences were aligned with Clustal Omega and BLOSUM62 protein weight matrix was used. Evolutionary relationships were calculated using the Neighbor-Joining method and the evolutionary distances were computed using the Poisson correction method. All positions containing gaps and missing data were eliminated and overall the final dataset contains total of 296 positions in the final dataset. The percentage of replicate trees in which the associated taxa clustered together in the bootstrap test are shown next to the branches. Bacterial (○), fungal (□), plant (Δ), insect (▲), and mammal (■) sequences. Amoebae and C. *elegans* sequences are marked with filled diamond (◆).

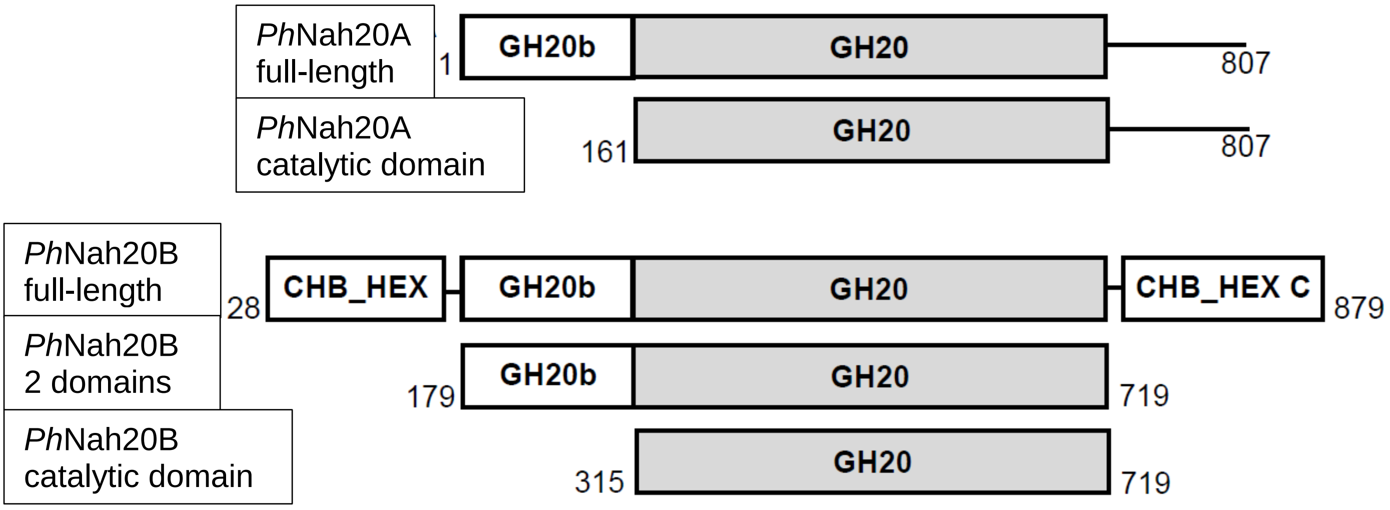

Fig S3. Schematic domain architecture of full-length and truncated variants of *Ph*Nah20A and *Ph*Nah20B. Catalytic domains of the GH20 enzymes are grey. Predicted start and end amino acid positions of the domains are shown.

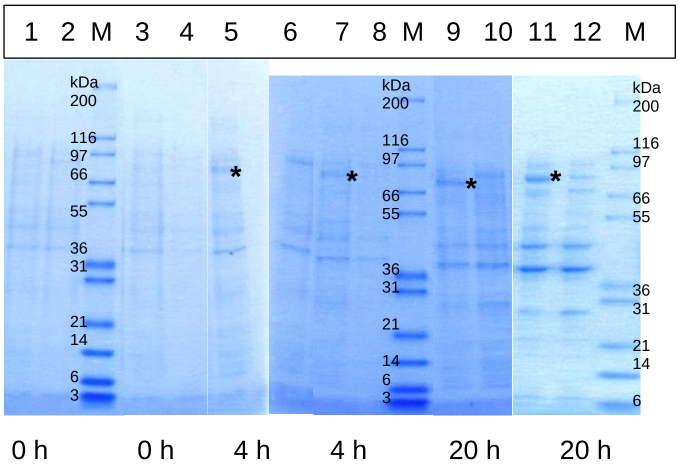

Fig S4. IPTG-induced *E. coli* transformants growing in LB analysed by SDS-PAGE. Aliquots (10 μL) of the cultures were removed at different time points, incubated 10 min at 80 °C with sample buffer and applied to the gels. Expression product (*Ph*Nah20A) is indicated by an asterisk. Strains: *E. coli* BL21(DE3) on lanes 1–2 (time of induction 0 h), 5–6 (4 h), 9–10 (20 h) and *E. coli* BL21(DE3)ΔlacZ 3–4 (0 h), 7–8 (4 h), 11–12 (20 h). M – Mark12 protein standard (Thermo Fisher Scientific, USA); lanes 1, 3, 5, 7, 9, 11 – *Ph*Nah20A; lanes 2, 4, 6, 8, 12 – *Ph*Nah20B.

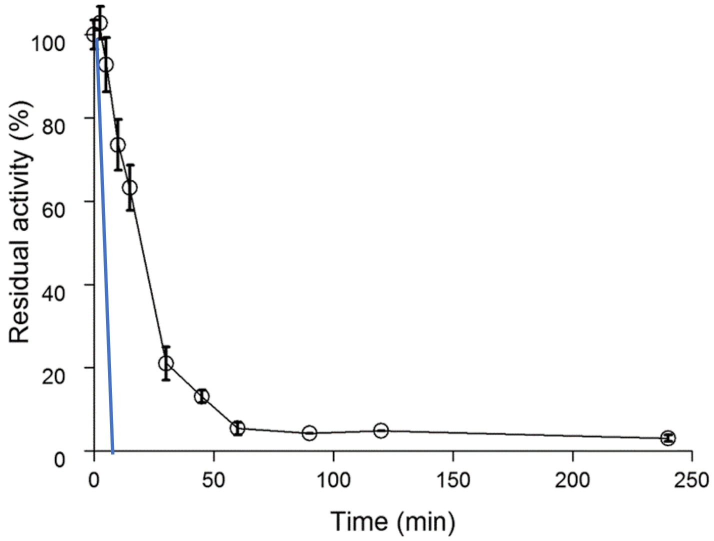

Fig S5. Inactivation of 5 μg mL^{-1} *Ph*Nah20A at 50 °C and pH 6.0 in the presence of 0.5% BSA. The trendline showing the inactivation of *Ph*Nah20A in the absence of BSA is indicated by the blue line. The initial velocity of *p*NPGlcNAc release remained close to 100% even without BSA if the measurement was started immediately. If the diluted enzyme was incubated 5 min at 50 °C prior the measurement in the absence of BSA, the activity was under the detection limit.

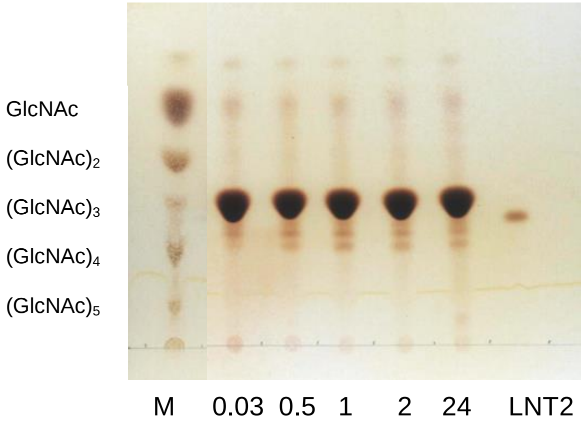

Fig S6. Time course of transglycosylation by *S. plicatus* Hex (10 U mL^{-1}) with 100 mM NAG-oxazoline as donor and 200 mM lactose as acceptor (see Materials and methods for details). Chitooligosaccharides (M) and lacto-*N*-triose II (LNT2) are used as references. The M lane was from the same TLC plate.

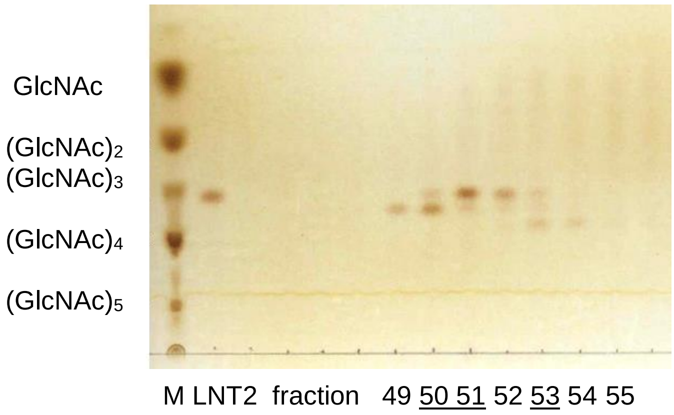

Fig S7. TLC of trisaccharide-containing fractions of the *Ph*Nah20A reaction (2 h; Figure 7A) separated by gel-permeation chromatography. Transglycosylation reactions of *Ph*Nah20A with NAG-oxazoline and lactose, separation and purification of products are described in Materials and methods. Chitooligosaccharides (M) and LNT2 as used reference; TLC of fractions 49–55 from gel-permeation are shown. Fractions 49 and 50 contain predominantly **1**, 51 and 52 **2. 3** was in fractions 53 and 54. Samples underlined were analysed by NMR (see Figures S8–10).

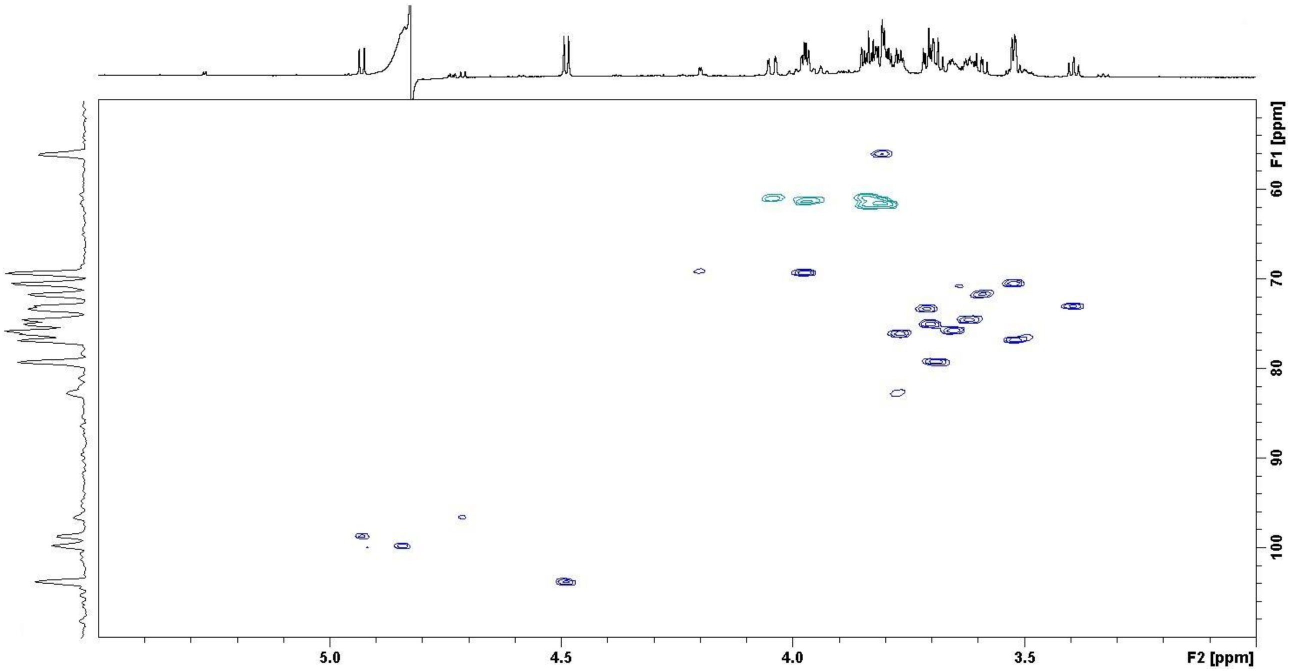

Fig S8. HSCQ spectrum of the chromatographic fraction 50 (see Figure S7) containing over 80% of compound 1. The main contaminant is **2** (LNT2). The ^{1}H projection is from a separate experiment.

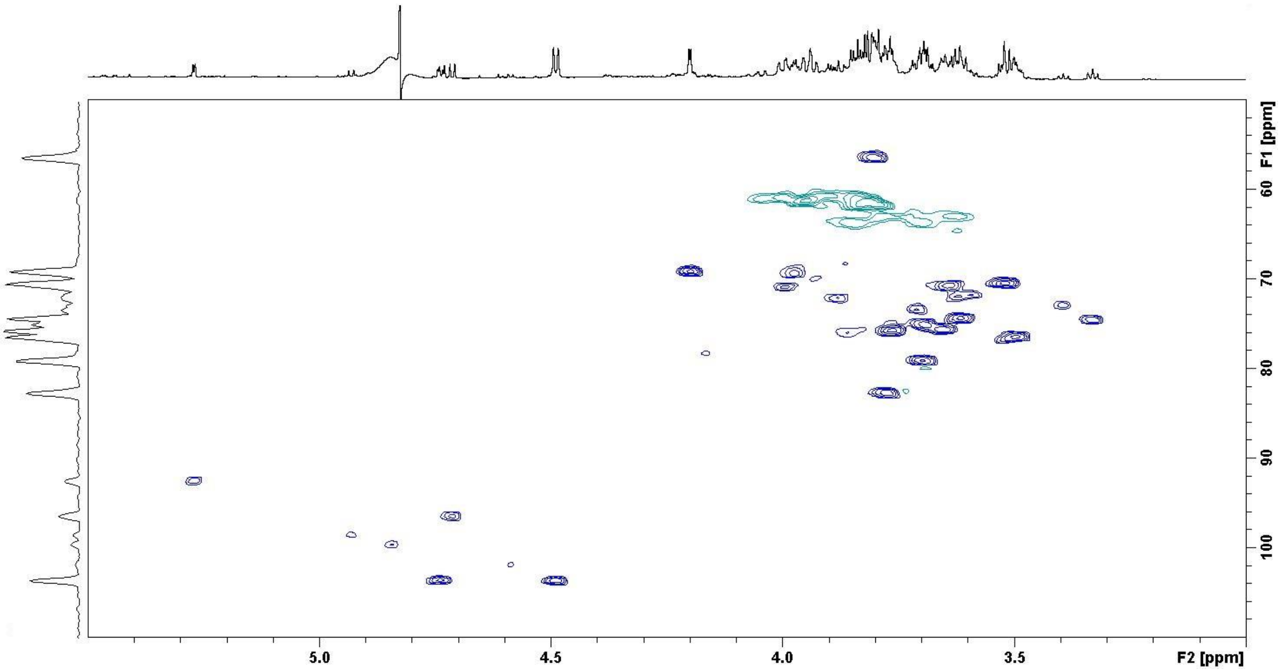

Fig S9. HSCQ spectrum of the chromatographic fraction 51 (see Figure S7) containing over 75% of 2 (LNT2). The main contaminant is **1**. The ^{1}H projection is from a separate experiment.

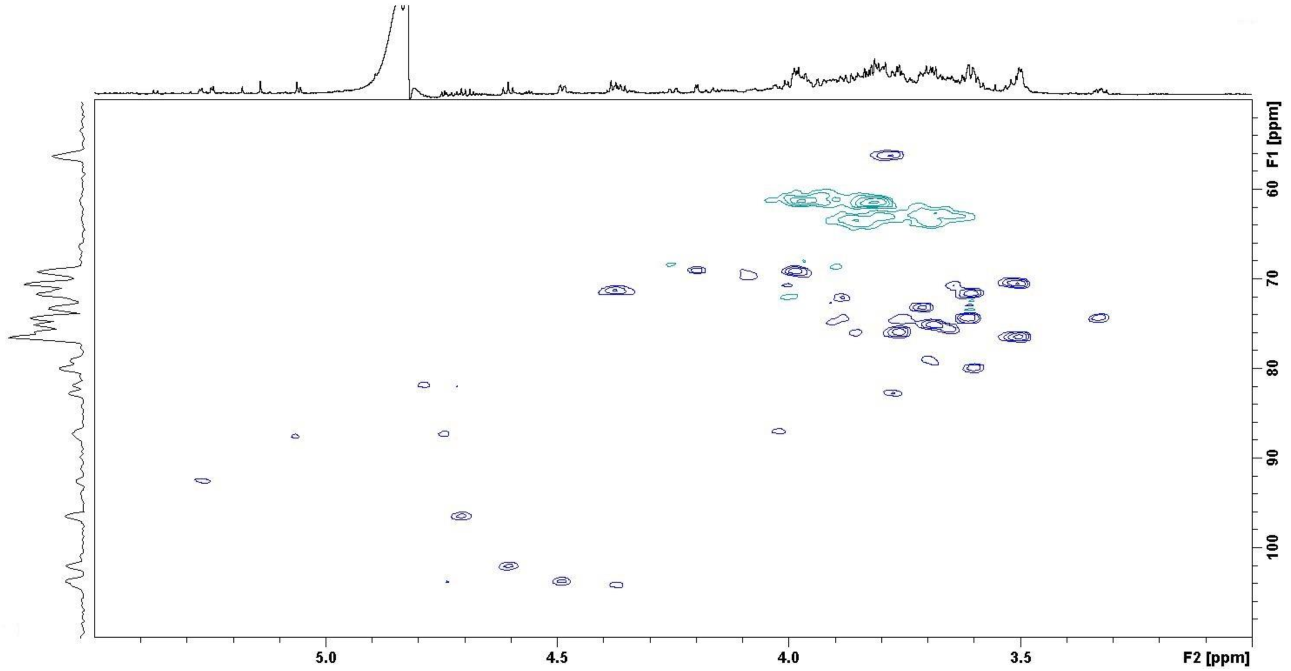

Fig S10. HSCQ spectrum of the chromatographic fraction 53 (see Figure S7) containing primarily 3. The main contaminant is **2** (LNT2). The ^{1}H projection is from a separate experiment.

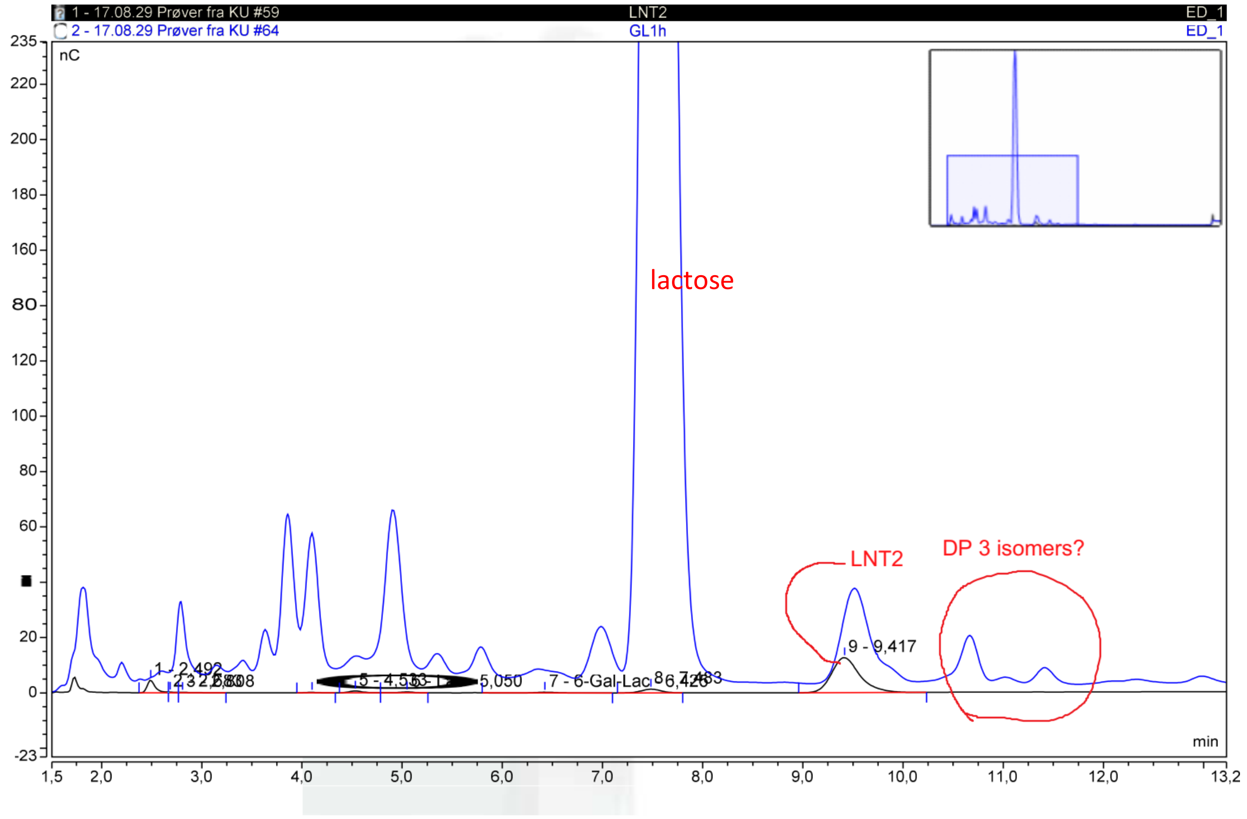

Fig S11. Extraction of HPAEC-PAD analysis of transglycosylation products by *Ph*Nah20A (10 U mL^{-1}) reacting 2 h with 100 mM NAG-oxazoline as donor and 200 mM lactose as acceptor (blue line). Lacto-*N*-triose II (LNT2) was used as reference (black line). The full chromatogram is shown in the top right corner.

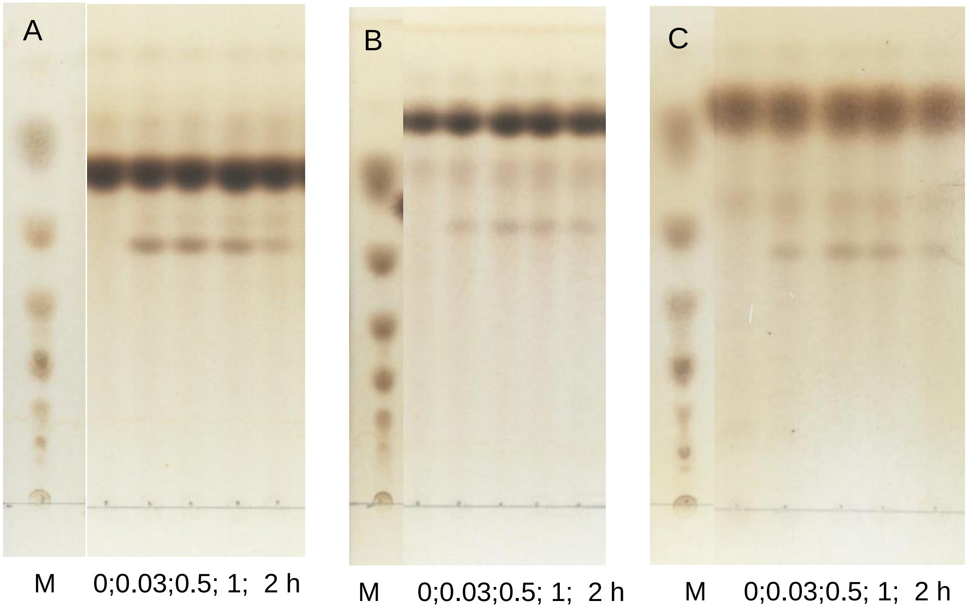

Fig S12. Time course of transglycosylation by *Ph*Nah20A (10 U mL^{-1}) with 100 mM NAG-oxazoline as donor and 200 mM D-glucose (A), 2-deoxy-D-glucose (B) or L-fucose (C) as acceptor. Chitooligosaccharides of DP 1–6 (M) are used as reference. The reference lane originates from the same TLC plate as samples.

Table S1. BLAST analysis of putative β-NAHAs (EC 3.2.1.52) from *P. hydrolytica*. Protein names, NCBI accession numbers, length, calculated molecular weight and information of protein sequences with the highest identity (including query coverage and E values) based on BLAST analysis are shown.

Proposed protein name	NCBI accession	GH family	Length (aa)	Calculated Mw (Da)	Proteins of highest identity				
					Name and organism	NCBI accession	Query coverage	E value	Identity (%)
*Ph*Nah20A	WP_068373836.1	GH20	807	91938	β-NAHA of *Lacimicrobium alkaliphilum*	WP_062475767.1	96%	0.0	54
					β-NAHA of *Bowmanella denitrificans*	WP_102795163.1	99%	0.0	53
					β-NAHA of *Lacimicrobium alkaliphilum*	WP_099034787.1	97%	0.0	52
					β-NAHA of *Lacimicrobium* sp. SS2-24	WP_088329680.1	97%	0.0	51
					β-NAHA of *Pseudoalteromonas* sp. R3	WP_082353221.1	87%	0.0	49
					β-NAHA of *Shewanella woodyi*	WP_012326528.1	99%	0.0	45
					β-NAHA of *Pseudoalteromonas rubra*	WP_125721644.1	99%	0.0	45
					hypothetical protein of marine sediment metagenome	KKL52452.1	100%	2e-125	47
					β-NAHA of compost metagenome	MNG99634.1	97%	0.0	45
					β-glycoside hydrolase of hydrothermal vent metagenome	VAX26736.1	92%	1e-97	44
*Ph*Nah20B	WP_082768773.1	GH20	879	98142	chitobiase of *Aliiglaciecola lipolytica*	WP_008846397.1	96%	0.0	49
					β-NAHA of *Bowmanella denitrificans*	WP_146027115.1	95%	0.0	48
					β-NAHA of *Saliniradius amylolyticus*	WP_109339583.1	97%	0.0	48
					β-NAHA of *Shewanella algae*	WP_145839512.1	93%	0.0	47
					β-NAHA of *Alteromonas pelagimonata*	WP_083638293.1	98%	0.0	47
					β-NAHA of *Shewanella halifaxensis*	WP_012276260.1	92%	0.0	47
					hypothetical protein of marine metagenome	ECV28588.1	97%	0.0	47
					hypothetical protein of marine sediment metagenome	EDA75810.1	100%	4e-135	43
					hypothetical protein of marine metagenome	KKL70545.1	99%	4e-168	45

Table S2. Information on proteins flanking identified β-NAHAs (presented in Figure 1B).

Region	Number (Fig 1B)	NCBI accession	Predicted function
*Ph*Nah20A (3)	1	WP_068373829.1	LemA family protein
	2	WP_068373832.1	hypothetical protein
	4	WP_068373840.1	sodium:solute symporter, putative SLC5sbd family protein
	5	WP_068373843.1	RidA (reactive intermediate/imine deaminase A) family protein
	6	WP_068373847.1	D-aminoacylase
	7	WP_068373849.1	MurR/RpiR family transcriptional regulator
	8	WP_068375252.1	amino acid deaminase
	9	WP_068373852.1	sodium/proton-translocating pyrophosphatase
*Ph*Nah20B (5)	1	WP_068374714.1	TonB-dependent receptor
	2	WP_068374717.1	DUF1624 domain-containing protein, putative acyltransferase
	3	WP_068374723.1	glucose/galactose MFS transporter
	4	WP_068374726.1	hypothetical protein, putative BadF-type ATPase
	6	WP_068374732.1	LacI family DNA-binding transcriptional regulator
	7	WP_068374734.1	dCTP deaminase
	8	WP_068374735.1	iron-sulfur cluster carrier protein ApbC
	9	WP_082768774.1	methionine-tRNA ligase
	10	WP_068374736.1	TetR/AcrR family transcriptional regulator

Table S3. NMR assignment of **1**. The methyl of the GlcNAc acetyl group was at 2.090 ppm for ^{1}H and 22.81 ppm for ^{13}C and the carbonyl of the acetyl was at 176.06 ppm ^{13}C.

	**nuclei**	1	2	3	4	5	6
β-Gal*p*-(1,4-	**^{1}H**	4.49	3.59	3.71	3.98	3.77	3.78/3.85
	**^{13}C**	103.77	71.70	73.36	69.29	76.16	61.81
4)-β-Glc*p*-(1,1-	**^{1}H**	4.84	3.40	3.70	3.69	3.65	3.85/4.05
	**^{13}C**	99.82	72.98	75.01	79.21	75.78	61.02
β-Glc*p*NAc-(1,1-	**^{1}H**	4.93	3.81	3.62	3.52	3.52	3.81/3.97
	**^{13}C**	98.80	56.06	74.50	70.43	76.79	61.40

Table S4. ^{3}H-H coupling constants for **1** measured through DQF-COSY.

	H1-H2	H2-H3	H3-H4	H4-H5	H5-H6a	H5-H6b	H6a-H6b
β-Gal*p*-(1,4	8.1	10.4	3.5	nd	nd	nd	nd
4)-β-Glc*p*-(1,1	8.4	9.4	nd	nd	7.3	2.2	12.4
β-Glc*p*NAc-(1,1-	8.5	9.9	9.8	nd	8.3	nd	12.5

nd – not determined due to a spectral overlap

Table S5. PCR primers to isolate full-length β-NAHA encoding genes and indicated truncated variants. Underlined sequences are priming with pURI3-TEV expression vector.

Protein name	Variant	Primer name	Primer sequence
*Ph*Nah20A	full-length	pURITEVFwGl1026	5'-GGTGAAAACCTGTATTTCCAGGGCTATAAAATCATTATCGCTGGTCTTGGATTGTTA-3'
		pURITEVRevGl1026	5'-AAGCTTAGTTAGCTATTATGCGTATTAAGCACGGCATTTAAATGCCAAGGCCT-3'
	catalytic domain	pURITEVGl1026catalFw	5'-GGTGAAAACCTGTATTTCCAGGGCTTTGCCTGGCGTGGTTTGTTA-3'
		pURITEVRevGl1026	5'-AAGCTTAGTTAGCTATTATGCGTATTAAGCACGGCATTTAAATGCCAAGGCCT-3'
*Ph*Nah20B	full-length, wo signal peptide	pURITEVFwGl1378	5'-GGTGAAAACCTGTATTTCCAGGGCGCAGTGGCCGATTCAACGAGTGCT-3'
		pURITEVRevGl1378	5'-AAGCTTAGTTAGCTATTATGCGTATTACTCCTCCACTTTTTGCTGCATTGAGACA-3'
	2 GH20 domains	pURITEVGl13782domFw	5'-GGTGAAAACCTGTATTTCCAGGGCGTGATGTTTGAGCATTACG-3'
		pURITEVGl1378catalRev	5'-AAGCTTAGTTAGCTATTATGCGTATTTAAACCAAGCGCGCTCA-3'
	catalytic domain	pURITEVGl1378catalFw	5'-GGTGAAAACCTGTATTTCCAGGGCTATGAATTCCGCGGTTTACAT-3'
		pURITEVGl1378catalRev	5'-AAGCTTAGTTAGCTATTATGCGTATTTAAACCAAGCGCGCTCA-3'
