# Supplementary material for: Identification and Characterization of a β-N-Acetylhexosaminidase with a Biosynthetic Activity from the Marine Bacterium Paraglaciecola hydrolytica S66T
Source: Int J Mol Sci. 2020 Jan 9;21(2):417. doi: 10.3390/ijms21020417 (PMC7014002; doi:10.3390/ijms21020417)
Supplement: Supplementary file 1 [file ijms-21-00417-s001.zip › ijms-670241-SI-to conversion/Supplementary Information 2 Visnapuu et al updated.pdf]

Supplementary Information 2

Identification and Characterization of a β-*N*-Acetylhexosaminidase with a Biosynthetic Activity from the Marine Bacterium *Paraglaciecola hydrolytica* S66[T]

Triinu Visnapuu [1,2,*], David Teze [1], Christian Kjeldsen [3], Aleksander Lie [4,a], Jens Øllgaard Duus [3], Corinne André-Miral [5], Lars Haastrup Pedersen [4], Peter Stougaard [6,b] and Birte Svensson [1,*]

1 Department of Biotechnology and Biomedicine, Technical University of Denmark, Søltofts Plads, building 224, DK-2800, Kgs. Lyngby, Denmark; triinu.visnapuu@ut.ee (T.V.); david.teze@gmail.com (D.T.); bis@bio.dtu.dk (B.S.)
2 Institute of Molecular and Cell Biology, University of Tartu, Riia 23, 51010 Tartu, Estonia
3 Department of Chemistry, Technical University of Denmark, Kemitorvet, building 207, DK-2800, Kgs. Lyngby, Denmark; chkje@kemi.dtu.dk (C.K.); jduus@kemi.dtu.dk (J.Ø.D.)
4 Department of Chemistry and Bioscience, Aalborg University, Fredrik Bajers Vej 7, H, DK-9220, Aalborg, Denmark; lhp@bio.aau.dk (L.H.P.); al@kebony.com (A.L.)
5 Université de Nantes, CNRS, Unité de Fonctionnalité et Ingénierie des Protéines (UFIP), UMR 6286, F-44000 Nantes, France; corinne.miral@univ-nantes.fr (C.A.-M.)
6 Department of Plant and Environmental Sciences, University of Copenhagen, Thorvaldsensvej 40, DK-1871, Frederiksberg C, Denmark; pst@envs.au.dk (P.S.)
* Correspondence: triinu.visnapuu@ut.ee; Tel.: +372-737-5013 (T.V.); bis@bio.dtu.dk; Tel.: +45-4525-2740 (B.S.)

[a] Present affiliation: Kebony Norge AS, Havneveien 35, NO-3739, Skien, Norway
[b] Present affiliation: Department of Environmental Science - Environmental Microbiology and Circular Resource Flow, Aarhus University, Frederiksborgvej 399, building 7411, B2.12, DK-4000, Roskilde, Denmark

```
                                             10        20        30        40        50        60        70
                                     ....|....|....|....|....|....|....|....|....|....|....|....|....|....|
WP_082768773.1_Nah20B_P. hydro       ---------------------------------------------------------------------- 1
WP_068373836.1_Nah20A_P. hydro       ---------------------------------------------------------------------- 1
BAD00143.1_Nah20A_A. hydrophil       ---------------------------------------------------------------------- 1
BAF76001.1_NahB_A. hydrophila        ---------------------------------------------------------------------- 1
BAI94822.1_BbhI_B. bifidum           MRLRRVKAAIGSVLAAVTLLSMSLTGVTAAQASDDNLALNQTVTASSYEVATTAPEKAVDGDLGTRWGTA 70
AAQ05800.1_Hex20A_C. fimi            ---------------------------------------------------------------------- 1
BAI63641.1_Hex1t_Paenibacillus       ---------------------------------------------------------------------- 1
CAA55582.1_NahA_P. gingivalis        ---------------------------------------------------------------------- 1
BAA84281.1_Hex99_P. piscicida        ---------------------------------------------------------------------- 1
BAB84321.1_Hex86_P. piscicida        ---------------------------------------------------------------------- 1
AAB03808.1_Chb_S. marcescens         ---------------------------------------------------------------------- 1
AAS02154.1_GcnA_S. gordonii          ---------------------------------------------------------------------- 1
AAK98861.1_StrH_S. pneumoniae        ---MKHEKQQRFSIRKYAVGAASVLIGFAFQAQTVAADGVTPTTTENQPTIHTVSDSPQSSENRTEETPK 67
CAB87223.1_HexA_S. coelicolor        ---------------------------------------------------------------------- 1
AAC38798.3_SpHex_S. plicatus         ---------------------------------------------------------------------- 1
AKC34128.1_Hex1_uncultured Act       ---------------------------------------------------------------------- 1
AKC34129.1_Hex2_uncultured Bac       ---------------------------------------------------------------------- 1
AAC44672.1_ExoI_V. furnissii         ---------------------------------------------------------------------- 1
AAA88682.1_Chb_V. harveyi            ---------------------------------------------------------------------- 1
AAD30612.1_AtHex1_A. thaliana        ---------------------------------------------------------------------- 1
AAM91092.1_AtHex3_A. thaliana        ---------------------------------------------------------------------- 1
BAE99290.1_AtHex3_A. thaliana        ---------------------------------------------------------------------- 1
AAM13977.1_HexA_A. oryzae            ---------------------------------------------------------------------- 1
AAT99455.1_BmGlcNAcase3_B. mor       ---------------------------------------------------------------------- 1
AAC60521.1_BmChiGlcNAcase_B. m       ---------------------------------------------------------------------- 1
CCD73477.2_CeHEX-4_C. elegans        ---------------------------------------------------------------------- 1
AAA34346.2_Hex1_C. albicans          ---------------------------------------------------------------------- 1
ADU20405.1_βHex_C. annuum            ---------------------------------------------------------------------- 1
ABB18373.1_CpHEX1_C. posadasii       ---------------------------------------------------------------------- 1
AAA33230.1_NagA_D. discoideum        ---------------------------------------------------------------------- 1
AAF46406.1_Hex2_D. melanogaste       ---------------------------------------------------------------------- 1
AAF47881.1_Hex1_D. melanogaste       ---------------------------------------------------------------------- 1
AAM29423.1_Fdl_D. melanogaster       ---------------------------------------------------------------------- 1
CAE46968.1_HexA_E. histolytica       ---------------------------------------------------------------------- 1
AAB00965.1_HexA_H. sapiens           ---------------------------------------------------------------------- 1
AAA52645.1_HexB_H. sapiens           ---------------------------------------------------------------------- 1
AAC53246.1_HexA_M. musculus          ---------------------------------------------------------------------- 1
AAA18776.1_HexB_M. musculus          ---------------------------------------------------------------------- 1
ABI81756.1_Hex1_O. furnacalis        ---------------------------------------------------------------------- 1
AEQ33603.1_HexTF_T. flavus           ---------------------------------------------------------------------- 1
ACI15900.1_NagA_T. maritima          ---------------------------------------------------------------------- 1
AAK16587.1_CbsA_T. neapolitana       ---------------------------------------------------------------------- 1
CAA63123.1_HexB_S. scrofa            ---------------------------------------------------------------------- 1
```

Multiple sequence alignment of 43 full-length *N*-acetylhexosaminidase protein sequences. The multiple sequence alignment was composed by Clustal Omega (https://www.ebi.ac.uk/Tools/msa/clustalo/) and visualized by BioEdit (https://softfamous.com/bioedit/). Putative active site residues based on the crystal structure of Hex from *Streptomyces plicatus* [1] are shown by asterisks under the alignment. *Ph*Nah20A sequence of *Paraglaciecola hydrolytica* is marked with red.

```
                                          80        90       100       110       120       130       140
                               ....|....|....|....|....|....|....|....|....|....|....|....|....|....|
WP_082768773.1_Nah20B_P. hydro ---------------------------------------------------------------------- 1
WP_068373836.1_Nah20A_P. hydro ---------------------------------------------------------------------- 1
BAD00143.1_Nah20A_A. hydrophil ---------------------------------------------------------------------- 1
BAF76001.1_NahB_A. hydrophila  ---------------------------------------------------------------------- 1
BAI94822.1_BbhI_B. bifidum     QNKAANEWIEVGLGGTKTVKQINIDFERKDADQNITSFKVELKQGDTYTKVYQKDTRAKQQEIILLDQAQ 140
AAQ05800.1_Hex20A_C. fimi      ---------------------------------------------------------------------- 1
BAI63641.1_Hex1t_Paenibacillus ---------------------------------------------------------------------- 1
CAA55582.1_NahA_P. gingivalis  ---------------------------------------------------------------------- 1
BAA84281.1_Hex99_P. piscicida  ---------------------------------------------------------------------- 1
BAB84321.1_Hex86_P. piscicida  ---------------------------------------------------------------------- 1
AAB03808.1_Chb_S. marcescens   ---------------------------------------------------------------------- 1
AAS02154.1_GcnA_S. gordonii    ---------------------------------------------------------------------- 1
AAK98861.1_StrH_S. pneumoniae  AELQPEAPKTVETETPATDKVASLPKTEEKPQEEVSSTPSDKAEVVTPTSAEKETANKKEEEASPKKEEA 137
CAB87223.1_HexA_S. coelicolor  ---------------------------------------------------------------------- 1
AAC38798.3_SpHex_S. plicatus   ---------------------------------------------------------------------- 1
AKC34128.1_Hex1_uncultured Act ---------------------------------------------------------------------- 1
AKC34129.1_Hex2_uncultured Bac ---------------------------------------------------------------------- 1
AAC44672.1_ExoI_V. furnissii   ---------------------------------------------------------------------- 1
AAA88682.1_Chb_V. harveyi      ---------------------------------------------------------------------- 1
AAD30612.1_AtHex1_A. thaliana  ---------------------------------------------------------------------- 1
AAM91092.1_AtHex3_A. thaliana  ---------------------------------------------------------------------- 1
BAE99290.1_AtHex3_A. thaliana  ---------------------------------------------------------------------- 1
AAM13977.1_HexA_A. oryzae      ---------------------------------------------------------------------- 1
AAT99455.1_BmGlcNAcase3_B. mor ---------------------------------------------------------------------- 1
AAC60521.1_BmChiGlcNAcase_B. m ---------------------------------------------------------------------- 1
CCD73477.2_CeHEX-4_C. elegans  ---------------------------------------------------------------------- 1
AAA34346.2_Hex1_C. albicans    ---------------------------------------------------------------------- 1
ADU20405.1_βHex_C. annuum      ---------------------------------------------------------------------- 1
ABB18373.1_CpHEX1_C. posadasii ---------------------------------------------------------------------- 1
AAA33230.1_NagA_D. discoideum  ---------------------------------------------------------------------- 1
AAF46406.1_Hex2_D. melanogaste ---------------------------------------------------------------------- 1
AAF47881.1_Hex1_D. melanogaste ---------------------------------------------------------------------- 1
AAM29423.1_Fdl_D. melanogaster ---------------------------------------------------------------------- 1
CAE46968.1_HexA_E. histolytica ---------------------------------------------------------------------- 1
AAB00965.1_HexA_H. sapiens     ---------------------------------------------------------------------- 1
AAA52645.1_HexB_H. sapiens     ---------------------------------------------------------------------- 1
AAC53246.1_HexA_M. musculus    ---------------------------------------------------------------------- 1
AAA18776.1_HexB_M. musculus    ---------------------------------------------------------------------- 1
ABI81756.1_Hex1_O. furnacalis  ---------------------------------------------------------------------- 1
AEQ33603.1_HexTF_T. flavus     ---------------------------------------------------------------------- 1
ACI15900.1_NagA_T. maritima    ---------------------------------------------------------------------- 1
AAK16587.1_CbsA_T. neapolitana ---------------------------------------------------------------------- 1
CAA63123.1_HexB_S. scrofa      ---------------------------------------------------------------------- 1
```

```
                                      150       160       170       180       190       200       210
                               ....|....|....|....|....|....|....|....|....|....|....|....|....|....|
WP_082768773.1_Nah20B_P. hydro --------------------------MKGNNMGNTRRKFTLGSFCGLASLLCSSGLSAAVADSTSAQQQL 44
WP_068373836.1_Nah20A_P. hydro ---------------------------------------------------------------------- 1
BAD00143.1_Nah20A_A. hydrophil ------------------------------------MNLKHSLLAIAMSTVFAGPALAADAARQAVVDAL 34
BAF76001.1_NahB_A. hydrophila  --------------------------------------------------------MSAPLIHTRVDHRD 14
BAI94822.1_BbhI_B. bifidum     QASAVKVTVLSADGGTMNWVNVGINEISVYSAPKETVLDTADTNHMLGATMTASSNETATLTPDKAIDQN 210
AAQ05800.1_Hex20A_C. fimi      ---------------------------------------------------------------------- 1
BAI63641.1_Hex1t_Paenibacillus ---------------------------------------------------------------------- 1
CAA55582.1_NahA_P. gingivalis  ---------------------------------------------------------------------- 1
BAA84281.1_Hex99_P. piscicida  ----------------------------------MFAKKQIITASLSGLALLTASHYSFAHPHPDNLALR 36
BAB84321.1_Hex86_P. piscicida  ---------------------------------------------------------------------- 1
AAB03808.1_Chb_S. marcescens   -----------------------------------MNAFKLSALARLTATMGFLGGMGSAMADQQLVDQL 35
AAS02154.1_GcnA_S. gordonii    ---------------------------------------------------------------------- 1
AAK98861.1_StrH_S. pneumoniae  KEVDSKESNTDKTDKDKPAKKDEAKAEADKPETETGKERAATVNEKLAKKKIVSIDAGRKYFSPEQLKEI 207
CAB87223.1_HexA_S. coelicolor  ---------------------------------------------------------------------- 1
AAC38798.3_SpHex_S. plicatus   ---------------------------------------------------------------------- 1
AKC34128.1_Hex1_uncultured Act ---------------------------------------------------------------------- 1
AKC34129.1_Hex2_uncultured Bac ---------------------------------------------------------------------- 1
AAC44672.1_ExoI_V. furnissii   ---------------------------------------------------------MNYRIDFAVLSEH 13
AAA88682.1_Chb_V. harveyi      -------------------------------------MLKHSLIAASVITT-LAGCSSLQSSEQQVVNSL 32
AAD30612.1_AtHex1_A. thaliana  ---------------------------------------------------------------------- 1
AAM91092.1_AtHex3_A. thaliana  ---------------------------------------------------------------------- 1
BAE99290.1_AtHex3_A. thaliana  ---------------------------------------------------------------------- 1
AAM13977.1_HexA_A. oryzae      ---------------------------------------------------------------------- 1
AAT99455.1_BmGlcNAcase3_B. mor ---------------------------------------------------------------------- 1
AAC60521.1_BmChiGlcNAcase_B. m ---------------------------------------------------------------------- 1
CCD73477.2_CeHEX-4_C. elegans  ---------------------------------------------------------------------- 1
AAA34346.2_Hex1_C. albicans    ---------------------------------------------------------------------- 1
ADU20405.1_βHex_C. annuum      ---------------------------------------------------------------------- 1
ABB18373.1_CpHEX1_C. posadasii ---------------------------------------------------------------------- 1
AAA33230.1_NagA_D. discoideum  ---------------------------------------------------------------------- 1
AAF46406.1_Hex2_D. melanogaste ---------------------------------------------------------------------- 1
AAF47881.1_Hex1_D. melanogaste ---------------------------------------------------------------------- 1
AAM29423.1_Fdl_D. melanogaster ---------------------------------------------------------------------- 1
CAE46968.1_HexA_E. histolytica ---------------------------------------------------------------------- 1
AAB00965.1_HexA_H. sapiens     ---------------------------------------------------------------------- 1
AAA52645.1_HexB_H. sapiens     ---------------------------------------------------------------------- 1
AAC53246.1_HexA_M. musculus    ---------------------------------------------------------------------- 1
AAA18776.1_HexB_M. musculus    ---------------------------------------------------------------------- 1
ABI81756.1_Hex1_O. furnacalis  ---------------------------------------------------------------------- 1
AEQ33603.1_HexTF_T. flavus     ---------------------------------------------------------------------- 1
ACI15900.1_NagA_T. maritima    ---------------------------------------------------------------------- 1
AAK16587.1_CbsA_T. neapolitana ---------------------------------------------------------------------- 1
CAA63123.1_HexB_S. scrofa      ---------------------------------------------------------------------- 1
```

```
                                          220       230       240       250       260       270       280
                                ....|....|....|....|....|....|....|....|....|....|....|....|....|....|
WP_082768773.1_Nah20B_P. hydro  LDNIAAHMGLTYVINNNISVENCQQDTGSAQCYQGQIQLTFK-QDVALKDWQIYFSNTSNIKWDGSELFD 113
WP_068373836.1_Nah20A_P. hydro  ---------------------------------------------------------------------- 1
BAD00143.1_Nah20A_A. hydrophil  ASNLVVKYEVVTNDGAGAGLDCQALGSEWASCGVAKLHLTNTGADVTSKDWSIYVSSIRRIQRVDNDQFT 104
BAF76001.1_NahB_A. hydrophila   GDDVWFRFTLSQLP---------------------------------AAPWKLHFCLARQIDPHSIEGGS 51
BAI94822.1_BbhI_B. bifidum     RTGRNNRWASGYETPSNIWLKAEFPRLTAVKDIRIYFFERDVNPKPTNVQSFDLSYTDSEGTEHTLKSGY 280
AAQ05800.1_Hex20A_C. fimi       ---------------------------------------------------------------------- 1
BAI63641.1_Hex1t_Paenibacillus  ---------------------------------------------------------------------- 1
CAA55582.1_NahA_P. gingivalis   ---------------------------------------------------------------------- 1
BAA84281.1_Hex99_P. piscicida   WQVVDHGIGENIFLGSLMITNNGLEPLGESGWALYFSSVRPPASVLPDSDPNGQYARQHIAAQNLSLENA 106
BAB84321.1_Hex86_P. piscicida   ---------------------------------------------------------------------- 1
AAB03808.1_Chb_S. marcescens    SQ-LKLNVKMLDNRAGENGVDCAALGADWASCNRVLFTLSNDGQAIDGKDWVIYFHSPRQTLRVDNDQFK 104
AAS02154.1_GcnA_S. gordonii     ---------------------------------------------------------------------M 1
AAK98861.1_StrH_S. pneumoniae   IDKAKHYGYTDLHLLVGNDGLRFMLDDMSITANGKTYASDDVKRAIEKGTNDYYNDPNGNHLTESQMTDL 277
CAB87223.1_HexA_S. coelicolor   ------------------------------------------------------------MRPHRRHHRT 10
AAC38798.3_SpHex_S. plicatus    ---------------------------------------------------------------------- 1
AKC34128.1_Hex1_uncultured Act  ---------------------------------------------------------------------- 1
AKC34129.1_Hex2_uncultured Bac  ---------------------------------------------------------------------- 1
AAC44672.1_ExoI_V. furnissii    PQFCRFGLTLHNLSDQ------------------------------DLKAWSLHFTIDRYIQPDSIS-HS 52
AAA88682.1_Chb_V. harveyi       ADNLDIQYEVLTNHGANEGLACQDMGAEWASCNKVNMTLVNQGEAVDSKDWAIYFHSIRLILDVDNEQFK 102
AAD30612.1_AtHex1_A. thaliana   ---------------------------------------------------------------------- 1
AAM91092.1_AtHex3_A. thaliana   ---------------------------------------------------------------------- 1
BAE99290.1_AtHex3_A. thaliana   ---------------------------------------------------------------------- 1
AAM13977.1_HexA_A. oryzae       ---------------------------------------------------------------------- 1
AAT99455.1_BmGlcNAcase3_B. mor  ---------------------------------------------------------------------- 1
AAC60521.1_BmChiGlcNAcase_B. m  ----------------MWLQAICIYTVFIIIGC-GIPTAAEE--------------------HSLWRWTC 33
CCD73477.2_CeHEX-4_C. elegans   ---------------------------------------------------------------------- 1
AAA34346.2_Hex1_C. albicans     ---------------------------------------------------------------------- 1
ADU20405.1_βHex_C. annuum       ---------------------------------------------------------------------- 1
ABB18373.1_CpHEX1_C. posadasii  ---------------------------------------------------------------------- 1
AAA33230.1_NagA_D. discoideum   ---------------------------------------------------------------------- 1
AAF46406.1_Hex2_D. melanogaste  -----MRFSGYNRYQCFCSAVGSLLLLSLLSFAVGAALTRADDVGSDADAG-----------SASRKWLC 54
AAF47881.1_Hex1_D. melanogaste  ----------------MKSTRTALGVALLLALVSQLAAHSSD--------------------DLVYGYEC 34
AAM29423.1_Fdl_D. melanogaster  -----MSLAVSLRRALLVLLTGAIFILTVLYWNQGVTKAQAYNEALERPHSHHDASGFPIPVEKSWTYKC 65
CAE46968.1_HexA_E. histolytica  ---------------------------------------------------------------------- 1
AAB00965.1_HexA_H. sapiens      ---------------------------------------------------------------------- 1
AAA52645.1_HexB_H. sapiens      ------------------------------------------------------MELCGLGLPRPPMLLA 16
AAC53246.1_HexA_M. musculus     ---------------------------------------------------------------------- 1
AAA18776.1_HexB_M. musculus     -------------------------------------------------------------MPQSP---- 5
ABI81756.1_Hex1_O. furnacalis   ----------------MWSRRIPLFIFGVLV---LILSVAAE--------------------DVVWRWSC 31
AEQ33603.1_HexTF_T. flavus      ---------------------------------------------------------------------- 1
ACI15900.1_NagA_T. maritima     ---------------------------------------------------------------------- 1
AAK16587.1_CbsA_T. neapolitana  ---------------------------------------------------------------------- 1
CAA63123.1_HexB_S. scrofa       ---------------------------------------------------------------------- 1
```

```
                                       290       300       310       320       330       340       350
                               ....|....|....|....|....|....|....|....|....|....|....|....|....|....|
WP_082768773.1_Nah20B_P. hydro IQHTNGDIHVITPVS--QNLIKKGSYAIDFKGIGSLVNESEAFPNYFISATDLQARVIQMTVPLVDSDTQ 181
WP_068373836.1_Nah20A_P. hydro ---------------MYKIIIAGLGLLWSITSHGMQPLNLMPYPSEVQQQAGHFTLPSQLKVFTQH---- 51
BAD00143.1_Nah20A_A. hydrophil ITHLTGDLYRLTPTEKFQGFAKDATVEVPLVVEYWVLFESDIMPNWYVASEGAEPKVLASMSNIDDASTY 174
BAF76001.1_NahB_A. hydrophila  LERQSGSYKVMVPHEGVS--ELRFCCRN------TPLKHDSDMPKGFFLSLESKGQLQPLVLKGEDLGLT 113
BAI94822.1_BbhI_B. bifidum     AMTASGAGYVADVVIQLDQAVNARSLKLSNFAIKSSEYNNVSVAEWEAYSNDQAEPGATLDSVVSDLESN 350
AAQ05800.1_Hex20A_C. fimi      ----------------------------------MPDVAVIPRPVLLETTDGPPFVLTAATILVVDS--- 33
BAI63641.1_Hex1t_Paenibacillus -------------------MMSFIP---ESASASTSQPSILPKPVSYTVG-SGQFVLTKNASIFVAGNNV 47
CAA55582.1_NahA_P. gingivalis  ---MKRLTFGACICCLLSLMACSQKAKQVQIPEYDKGINIIPLPMQLTES-DDSFEVDDKTTICVS---- 62
BAA84281.1_Hex99_P. piscicida  DDAKSGDYFVLKPVAGFAPIQPGESRTIEIVAQYWQMLKNDSPSGFHISYNNQAPQAVLVDVMMDPSDPK 176
BAB84321.1_Hex86_P. piscicida  ---MN-------QSVFYTFLQSSLLLFSLLFTPLCTALQITPKPLSASFG-NGHFNLTHDSKITFN---- 55
AAB03808.1_Chb_S. marcescens   IAHLTGDLYKLEPTAKFSGFPAGKAVEIPVVAEYWQLFRNDFLPRWYATSGDAKPKMLANTD-TENLDQF 173
AAS02154.1_GcnA_S. gordonii    ATFLGLSSKQEKALVRLDKYLNLGEIAVSLVTDSATSIKVEGRQGYYQVSYKQPHQLYRALALLSAALRS 71
AAK98861.1_StrH_S. pneumoniae  INYAKDKGIGLIPTVNSPGHMDAILNAMKELGIQNPNFSYFGKKSARTVDLDNEQAVAFTKALIDKYAAY 347
CAB87223.1_HexA_S. coelicolor  TPRITRLLGSLLLVAAVGAMTTGAAPVRKAAAEPTPLDRVIPAPASVEPG-GAPYRITRGTHIRVD---- 75
AAC38798.3_SpHex_S. plicatus   -------------------MTTGAAPDRKAPVRPTPLDRVIPAPASVDPG-GAPYRITRGTHIRVD---- 46
AKC34128.1_Hex1_uncultured Act -------------------MSP-------------VMNTVIPRPSSVLGT-GRSFELPADARILVQP--- 34
AKC34129.1_Hex2_uncultured Bac ---MK---------TIASLLIAGSLATSAAEPP-----ALIPQPVSVQPG-EGSFKFSAGTGIRH----- 47
AAC44672.1_ExoI_V. furnissii   QIHQVGSFCSLTPEQDVINSNSHFYCEFSIKTAPFPFHYYTDGIKAAFVQINDVEPRVRHDVIVTPIALA 122
AAA88682.1_Chb_V. harveyi      ISRVTGDLHKLEPTDKFDGFAAGEEVVLPLVGEYWQLFETDFMPGAFVSAPNAEPKMIASLN-TEDVASF 171
AAD30612.1_AtHex1_A. thaliana  -------MLTLSKFHVILIPILFFITLLSPLFSIALPINIWPKPR----FLSWPQHKAIALSPNFT---- 55
AAM91092.1_AtHex3_A. thaliana  -----MRGSGAKIAGVLPLFMLFIAGTISAFEDIER-LRIWPLPAQVSHGGRRMYLSGDFKLVTEGSK-- 62
BAE99290.1_AtHex3_A. thaliana  -------MSTNLLRLILLFITLSITSSLSTPSPADSPPYLWPLPAEFSFGNETLSVDPTVTLIVAGN--- 60
AAM13977.1_HexA_A. oryzae      ---------------MRISQICTVLSTVTS--AVAVGVNPLPAPR----EISWGSSGPKSIAGELQ---- 45
AAT99455.1_BmGlcNAcase3_B. mor -----MFRLFVYLNILGAFLVTGLHIVEPGPEYPASKGAIWPRPQMQSIEIPYYKFDSDILEIKVVDHDC 65
AAC60521.1_BmChiGlcNAcase_B. m E-----NNRCTKIRNEPENKEPVLSLEACKMFCD-DYGLLWPKPT-IETNLGNFLSKINMNTIDIQ---- 92
CCD73477.2_CeHEX-4_C. elegans  ------MHKMSKLCFLALLSVTFMLIFVLTTPYSNDRSSYAAYEGGIDPRKTRQFKNIIVHLDLKG---- 60
AAA34346.2_Hex1_C. albicans    -------------MVLDKMIIFHLLLWLCNVVVHAAKVEILPAPQ----SVTWENDTAIIINPRLL---- 49
ADU20405.1_βHex_C. annuum      -------MRGDTTFPFILS---LFVIFITQTIATNYPINVWPKPT----TFNWPNPKIHLPLPNFT---- 52
ABB18373.1_CpHEX1_C. posadasii ---------------MRFAYLATLAGSLLAGLAQAVKVNPLPAPQ----HIKWGESGPQYLDWNVK---- 47
AAA33230.1_NagA_D. discoideum  --------------MIKKIILFFAVLIA--IVIGQQPLNVVPYPQQVSIGTCVIPVAPGSILIESN---- 50
AAF46406.1_Hex2_D. melanogaste SRTDICTAEGEMVAGLQYAPEIFESQRDCRLSCG-KYGAIWPMPTGKECTISHRRVRFDPWKVRFH---- 119
AAF47881.1_Hex1_D. melanogaste R-----SGYCQKVELSEENYVKAISLPVCRLFCGSSIGTLWPKPT-GTVRLDTLMRQVDISFIDFN---- 94
AAM29423.1_Fdl_D. melanogaster E-----NDRCMRVGHHGKS-AKRVSFISCSMTCG--DVNIWPHPT-QKFLLSSQTHSFSVEDVQLH---- 122
CAE46968.1_HexA_E. histolytica --------------MLFIFIVFISSVFAGNGLNVQNQLLLMPYPSSVSFQWKSPLAIALTSSIQLN---- 52
AAB00965.1_HexA_H. sapiens     ---------MTSSRLWFSLLLAAAFAGRAT--------ALWPWPQNFQTSDQRYVLYPNNFQFQYD---- 49
AAA52645.1_HexB_H. sapiens     LLLATLLAAMLALLTQVALVVQVAEAARAPSVSAKPGPALWPLPLSVKMTPNLLHLAPENFYISHS---- 82
AAC53246.1_HexA_M. musculus    ---------MAGCRLWVSLLLAAALACLAT--------ALWPWPQYIQTYHRRYTLYPNNFQFRYH---- 49
AAA18776.1_HexB_M. musculus    -------RSAPGLLLLQALVSLVSLALVAP---ARLQPALWPFPRSVQMFPRLLYISAEDFSIDHS---- 61
ABI81756.1_Hex1_O. furnacalis  D-----NGKCVKLKNDPRSSEPALSLEACKMFCN-EYGLLWPRPT-GEADLGNFLSKINLNSIEVK---- 90
AEQ33603.1_HexTF_T. flavus     ---------------MRVS-LVTVSCLASA--VAGVAVNPLPAPR----SITWGTSGPISVPHNVD---- 44
ACI15900.1_NagA_T. maritima    --------MDVDLGKLFFCGFNDFNEEVKEIIRKYRPTGILIYPG----VLSKEYLLMDFMSFLSK---- 54
AAK16587.1_CbsA_T. neapolitana --------MDVDLGKLFFCGFDDFNEEAREVIQKYRPAGVLIYPG----VLSKEYLFLDFMNFLSR---- 54
CAA63123.1_HexB_S. scrofa     --------MEVATRPPAAAGGSGGAERWARDTSGAESLGLWPLPFAVDISPRSLHLSPNNFFFGHS---- 58
```

```
                                       360       370       380       390       400       410       420
                               ....|....|....|....|....|....|....|....|....|....|....|....|....|....|
WP_082768773.1_Nah20B_P. hydro LLVYPHVLPFVTPEQTKR------RVGDKVPLATPEVMFEHYASINALAAESATTAPRLIPHTVSSQFDA 245
WP_068373836.1_Nah20A_P. hydro ---------------------------------------------VSEARCELIKEQLEQLQQVEVLWVT 76
BAD00143.1_Nah20A_A. hydrophil EKPMP-------ADGWKR------TKDDNNILMNSETRFAANQTSTLLPAGKIDDRILPSPMKQVVKAGP 231
BAF76001.1_NahB_A. hydrophila NPPSP--------------------------------------GPDLPQVEAVN-GLVPQPAHLIYTEGN 144
BAI94822.1_BbhI_B. bifidum    HLTIETDTDTLALPTVPDGYTVKFNGADYEQLIAADGTVNHPLVDKTVQVAYVVTDTATGNTKTTSDIPY 420
AAQ05800.1_Hex20A_C. fimi     -------------------------------------------APELVAVGVLAADLLGRLSGRPVEVRY 60
BAI63641.1_Hex1t_Paenibacillus G-----------------------------------------ETDELFNIGQALAKKLNASTGYTISVVK 76
CAA55582.1_NahA_P. gingivalis -------------------------------------------AEELKPIAKLLADKLRASADLSLQIEI 89
BAA84281.1_Hex99_P. piscicida QTKQAVN-----------------------------------DNMPVQTSAIRFAENSATHMALPLKNRL 211
BAB84321.1_Hex86_P. piscicida -------------------------------------------QHQAQSVAQQLATFLRSPTGYQLPVSQ 82
AAB03808.1_Chb_S. marcescens  VAPFT-------GDQWKR------TKDDKNILMTPASRFVSNADLQTLPAGALRGKIVPTPMQVKVHAQD 230
AAS02154.1_GcnA_S. gordonii   ------------------------------------------GQDEVQIEEEAAYEDLAYMADCSRNAVL 99
AAK98861.1_StrH_S. pneumoniae FAKKTEIFNIGLDEYANDATDAKGWSVLQADKYYPNEGYPVKGYEKFIAYANDLARIVKSHGLKPMAFND 417
CAB87223.1_HexA_S. coelicolor ------------------------------------------DSREARRVGDYLADLLRPATGYRLPVTS 103
AAC38798.3_SpHex_S. plicatus  ------------------------------------------DSREARRVGDYLADLLRPATGYRLPVTA 74
AKC34128.1_Hex1_uncultured Act ------------------------------------------NAPEVAQIGEFLAAALRPATGLALPVTA 62
AKC34129.1_Hex2_uncultured Bac -------------------------------------------DRALESEAKLLASDLAKLTDSQPKTVV 74
AAC44672.1_ExoI_V. furnissii  SPYRE--------------------------------------RSEIPATDAATLSLLPKPNHIERLDGE 154
AAA88682.1_Chb_V. harveyi     VTGLE-------GNNLKR------TPDDNNVFANAVSRFEKNEDLATQDVSTT---LLPTPMHVEAGKGK 225
AAD30612.1_AtHex1_A. thaliana ------------------------------------------ILAPEH---QYLSASVTRYHNLIRSENY 80
AAM91092.1_AtHex3_A. thaliana ----------------------------------------------YGDASGILKEGFDRMLGVVRLSHV 86
BAE99290.1_AtHex3_A. thaliana -----------------------------------------------GGGSLIIRAAFDRYMGIIFKHAS 83
AAM13977.1_HexA_A. oryzae     ------------------------------------------LRTDSDSADGIVADAWNRAWETIVALRW 73
AAT99455.1_BmGlcNAcase3_B. mor P----------------------------------------ILSNAVQRSLAVLREMLRIASPYVNRNAP 95
AAC60521.1_BmChiGlcNAcase_B. m ------------------------------------------ITKQGKSD-DLLTAAADRFKTLVSSSVP 119
CCD73477.2_CeHEX-4_C. elegans ------------------------------------------APPRVEYLIEFFKLLSKHHVDGILIEYE 88
AAA34346.2_Hex1_C. albicans   ------------------------------------------QANTSC---PLLEDAFVRTVSAIEKSKW 74
ADU20405.1_βHex_C. annuum     ------------------------------------------ISHPTH---RYLTPTVYRYRRLILSEHY 77
ABB18373.1_CpHEX1_C. posadasii ------------------------------------------YSGPRD---RTIIAAWRRTWGSIVQLRW 72
AAA33230.1_NagA_D. discoideum -----------------------------------------IESATFSVSMDRYTN-----LFFPFSNES 74
AAF46406.1_Hex2_D. melanogaste ------------------------------------------VVAPGEAATQFLRETNRLFVSNLLKECI 147
AAF47881.1_Hex1_D. melanogaste ------------------------------------------FNGIARQQ-KLWRAVEDRFMNMLEAQIP 121
AAM29423.1_Fdl_D. melanogaster ------------------------------------------VDTAHREVRKQLQLAFDWFLKDLRLIQR 150
CAE46968.1_HexA_E. histolytica -----------------------------------------VKSTCNTDCMNFLKSNFNHTISFPLQQQT 81
AAB00965.1_HexA_H. sapiens    ------------------------------------------VSSAAQPGCSVLDEAFQRYRDLLFGSGS 77
AAA52645.1_HexB_H. sapiens    ------------------------------------------PNSTAGPSCTLLEEAFRRYHGYIFGFYK 110
AAC53246.1_HexA_M. musculus   ------------------------------------------VSSAAQGGCVVLDEAFRRYRNLLFGSGS 77
AAA18776.1_HexB_M. musculus   ------------------------------------------PNSTAGPSCSLLQEAFRRYYNYVFGFYK 89
ABI81756.1_Hex1_O. furnacalis ------------------------------------------ILKKGATD-DLMEAAAKRFKEQVSLAIP 117
AEQ33603.1_HexTF_T. flavus    ------------------------------------------VKLPKN---AIINDAWERAWTSIKQLRW 69
ACI15900.1_NagA_T. maritima   -------------------------------------------EGDFLISSDHEGGQLEVLKYVPSSPGN 81
AAK16587.1_CbsA_T. neapolitana -------------------------------------------NGRFIVSSDHEGGQLEVLKYVPSFPGN 81
CAA63123.1_HexB_S. scrofa     ------------------------------------------PTSKAGSSCEILQEAFRRYYDFIFGFYK 86
```

```
                                        430       440       450       460       470       480       490
                               ....|....|....|....|....|....|....|....|....|....|....|....|....|....|
WP_082768773.1_Nah20B_P. hydro TQRVSVSQG------------------------IAVDEASLSQFSAAFQRLAKKGLNTQSTG-------- 283
WP_068373836.1_Nah20A_P. hydro AKDQAN---------------------------LLVEVEGVTQVMEVGGK-------------------- 99
BAD00143.1_Nah20A_A. hydrophil QVDFST-IK------------------------LNALDLPSDRAEALKTQLTKLGVTLSDTG-------- 268
BAF76001.1_NahB_A. hydrophila  FRLAERAHV------------------------GGPVPLASLLAHFN--ELTGLALTQDHD--------- 179
BAI94822.1_BbhI_B. bifidum     VVKGTNQQQE--------------GNNAKPTIIPEIAEWHSTSAAKLAASAVTKVVYDDDSLKAVVDEFV 476
AAQ05800.1_Hex20A_C. fimi      TEGGAPSV-------------------------VRLRLSED----------------------------- 76
BAI63641.1_Hex1t_Paenibacillus SNQPTAGS-------------------------IYLTTVGGN---------------------------- 93
CAA55582.1_NahA_P. gingivalis  GEEPSGN--------------------------AIYIGVDTA---------------------------- 105
BAA84281.1_Hex99_P. piscicida  VPQPHSVVTP--------------NDEFLTLMSQLTSISGAASLAKEATFLQTALRDILSGTFAIN---- 263
BAB84321.1_Hex86_P. piscicida  ADSTTKN---------------------------SIAFKIVD---------------------------- 97
AAB03808.1_Chb_S. marcescens   ADLRKG-VA------------------------LDLSTLVKPAADVVSQRFALLGVPVQTNG-------- 267
AAS02154.1_GcnA_S. gordonii    NLSSAK---------------------------------------------------------------- 105
AAK98861.1_StrH_S. pneumoniae  GIYYNSDTSFGSFDKDIIVSMWTGGWGGYDVASSKLLAEKGHQILNTNDAWCYVLGRNADGQGWYNLDQG 487
CAB87223.1_HexA_S. coelicolor  ---HGHGG-------------------------IRLRLAEG----------------------------- 116
AAC38798.3_SpHex_S. plicatus   ---HGHGG-------------------------IRLRLAGG----------------------------- 87
AKC34128.1_Hex1_uncultured Act GAAQERGT-------------------------IHLTTAAAD---------------------------- 79
AKC34129.1_Hex2_uncultured Bac KELTNALPS------------------------EILLDFSEK---------------------------- 92
AAC44672.1_ExoI_V. furnissii   FALTAGSQI------------------------SLQSSCAETAATWLKQELTHLYQWQPHDI-------- 192
AAA88682.1_Chb_V. harveyi      VDIADG-IA------------------------LPKDAFDATQFAAIQDRAEVVGVDVRG-D-------- 261
AAD30612.1_AtHex1_A. thaliana  SPLIS----------------------------YPVKLMKRYTLRN------------------------ 98
AAM91092.1_AtHex3_A. thaliana  ISG------------------------------DRNSSGTGGSALLQGLH-------------------- 106
BAE99290.1_AtHex3_A. thaliana  GRGSLL---------------------------SRIRFLKMVEYDITSLK-------------------- 106
AAM13977.1_HexA_A. oryzae      VPAATE---------------------------APISSFEPFP--TPTAG----------------ASKK 98
AAT99455.1_BmGlcNAcase3_B. mor QQVLDD---------------------------DTYDGPLKS---LSIYL-------------------- 115
AAC60521.1_BmChiGlcNAcase_B. m KGFSAK---------------------------AAGKSVTVYLVN------------------------- 137
CCD73477.2_CeHEX-4_C. elegans  DMFPYSG--------------------------------------------------------------- 95
AAA34346.2_Hex1_C. albicans    HP-------------------------------FPIDDFNTANGKNIKTS-------------------- 93
ADU20405.1_βHex_C. annuum      RHIIT----------------------------PSINLTSSTPLQH------------------------ 95
ABB18373.1_CpHEX1_C. posadasii TPAALE---------------------------APIPTFAPFIVGNGKRD-----------------AHS 98
AAA33230.1_NagA_D. discoideum  -----------------------------------EPSSNESFLLSVT---------------------- 87
AAF46406.1_Hex2_D. melanogaste RNCTLE---------------------------TSKQILVRSTVAN------------------------ 166
AAF47881.1_Hex1_D. melanogaste DRKVLA---------------------------RGGYRMSVNINTP------------------------ 140
AAM29423.1_Fdl_D. melanogaster LDYGGS---------------------------SSEPTVSESSSKSRHHA----------------DLEP 177
CAE46968.1_HexA_E. histolytica GLQDFKVSLF--------------KEIDLPRITPSVSSVITDVVVELS---------------------- 115
AAB00965.1_HexA_H. sapiens     WPRPYL---------------------------TGKRHTLEKNVLVVSVV-------------------- 100
AAA52645.1_HexB_H. sapiens     WHHEPA---------------------------EFQAKTQVQQLLVSITL-------------------- 133
AAC53246.1_HexA_M. musculus    WPRPSF---------------------------SNKQQTLGKNILVVSVV-------------------- 100
AAA18776.1_HexB_M. musculus    RHHGPA---------------------------RFRAEPQLQKLLVSITL-------------------- 112
ABI81756.1_Hex1_O. furnacalis  RGSTPK---------------------------LTGKAVDVYLVN------------------------- 135
AEQ33603.1_HexTF_T. flavus     VPQATE---------------------------APISSYEPFPTVAPTSASTASSTPLPSSSAAAVAARS 112
ACI15900.1_NagA_T. maritima    LAFGKN---------------------------------------------------------------- 87
AAK16587.1_CbsA_T. neapolitana LAAGKV---------------------------------------------------------------- 87
CAA63123.1_HexB_S. scrofa      WHQGSY---------------------------QLCFGTELQQLQVHV---------------------- 107
```

```
                                         500       510       520       530       540       550       560
                                ....|....|....|....|....|....|....|....|....|....|....|....|....|....|
WP_082768773.1_Nah20B_P. hydro  --------------------LPLKLQLN----LQSGLSTQAYQLTISEKQIFIEAS-------------- 315
WP_068373836.1_Nah20A_P. hydro  ----------------------------SGELIPTLHDNEQYHLSVSPRLITLKAQ-------------- 127
BAD00143.1_Nah20A_A. hydrophil  --------------------YPVTIKLG-----SKLKQAEGYDMTIGKKGTVIQGH-------------- 299
BAF76001.1_NahB_A. hydrophila   ----------------------AEIHL-----VEATLPPEGYELTITPEEIVIRAA-------------- 208
BAI94822.1_BbhI_B. bifidum     ADYKDFTGIKLTAKKGAAEAGAFNFVKTDSTAAIAQLGDEGYTMDIRADRVVAKSS-------------- 532
AAQ05800.1_Hex20A_C. fimi       ----------------------------------LPAGDEAYRLVVSEHRVDIDAR-------------- 98
BAI63641.1_Hex1t_Paenibacillus  ----------------------------------AALGNEGYDLITTSNQVTLTAN-------------- 115
CAA55582.1_NahA_P. gingivalis   ----------------------------------LPLKEEGYMLRSDKRGVSIIGK-------------- 127
BAA84281.1_Hex99_P. piscicida   -----------STVQNGPNLIKLQLDPNLDTNQDNQPDASGYKLEVDAFSGITITG-------------- 308
BAB84321.1_Hex86_P. piscicida   ----------------------------------APLSQEGYALSVTTEGVEIQAN-------------- 119
AAB03808.1_Chb_S. marcescens    --------------------YPIKTDIQPGKFKGAMAVSGAYELKIGKKEAQVIGF-------------- 303
AAS02154.1_GcnA_S. gordonii     ----------------------------KMIEVLALMGYSTFELYMEDTYEIENQP-------------- 133
AAK98861.1_StrH_S. pneumoniae   LNGIKNTPITSVPKTEGADIPIIGGMVAAWADTPSARYSPSHLFKLMRHFANANAEYFAADYESAEQALN 557
CAB87223.1_HexA_S. coelicolor   -----------------------------------PYGDEGYRLDSGREGVTITAR-------------- 137
AAC38798.3_SpHex_S. plicatus    -----------------------------------PYGDEGYRLDSGPAGVTITAR-------------- 108
AKC34128.1_Hex1_uncultured Act  ----------------------------------PALGAEGYELLVAPHGVLLAAP-------------- 101
AKC34129.1_Hex2_uncultured Bac  ----------------------------------TDLPPSGYELKVQPNGVVIRGK-------------- 114
AAC44672.1_ExoI_V. furnissii    --------------------GSADIVLR----TNPTLDEGAYLLSVDRKPIRLEAS-------------- 224
AAA88682.1_Chb_V. harveyi       --------------------LPVSITVVPADFTGELAKSGAYEMSIKGDGIVIKAF-------------- 297
AAD30612.1_AtHex1_A. thaliana   ----------------------LVVTVTDFSLPLHHGVDESYKLSIPIGSFSAHLL-------------- 132
AAM91092.1_AtHex3_A. thaliana   ------------------------VIISSSTDELEYGADESYKLVVPSPEKP---SYAQL---------- 139
BAE99290.1_AtHex3_A. thaliana   ------------------------IVVHSDSEELQLGVDESYTLMVSKKNEQSIVGAATI---------- 142
AAM13977.1_HexA_A. oryzae       SKR------------ASNSLQYVNVQVKDIEADLQHGVDESYTLDVEEDSDTITIN-------------- 142
AAT99455.1_BmGlcNAcase3_B. mor  --------------------------TSPCEEYPHFGMIESYNLTIAAD-STLRSS-------------- 144
AAC60521.1_BmChiGlcNAcase_B. m  --------------------------ENPYIREFSLDMDESYELYISSTSSDKVNATIR----------- 170
CCD73477.2_CeHEX-4_C. elegans   -------------------------------DIEEIRRDLHYSENDIRRIIQAAEVHN------------ 122
AAA34346.2_Hex1_C. albicans     ---------------------LVHIQVDDATVDLQLGVNESYTLKIN--TDGINIH-------------- 126
ADU20405.1_βHex_C. annuum       ----------------------LIISVSDVTSPLSHGVNESYSLSTPNGSSAAYIT-------------- 129
ABB18373.1_CpHEX1_C. posadasii  NRR----------------ILRVSVKVENTNVDLQHGVDESYTLQIRDKSDSIRIT-------------- 138
AAA33230.1_NagA_D. discoideum   --------------------------IYSDDETLQLGIDESYSLSIEQGSYQLKAT-------------- 117
AAF46406.1_Hex2_D. melanogaste  -----------------------------ESLVLDWPTDESYALVVRTTETATFVD-IQ----------- 195
AAF47881.1_Hex1_D. melanogaste  --------------------------DEPTPARLTLDTDESYTLDIDTDASGHVLANIT----------- 173
AAM29423.1_Fdl_D. melanogaster  AATLFGATFGVKKAGDLTSVQVKISVLKSGDLNFSLDNDETYQLSTQTEG-HRLQVEII----------- 235
CAE46968.1_HexA_E. histolytica  --------------------------SSNPMPKLQIGFDESYILEVTTNSISIKAV-------------- 145
AAB00965.1_HexA_H. sapiens      --------------------------TPGCNQLPTLESVENYTLTINDDQCLLLSE-------------- 130
AAA52645.1_HexB_H. sapiens      --------------------------QSECDAFPNISSDESYTLLVKEPVAVLKAN-------------- 163
AAC53246.1_HexA_M. musculus     --------------------------TAECNEFPNLESVENYTLTINDDQCLLASE-------------- 130
AAA18776.1_HexB_M. musculus     --------------------------ESECESFPSLSSDETYSLLVQEPVAVLKAN-------------- 142
ABI81756.1_Hex1_O. furnacalis   --------------------------ENPNEKAFSLEMDESYGLRVSPSGADRVNATIT----------- 168
AEQ33603.1_HexTF_T. flavus      QKRGHQKGSHEKRGHSQSTLRTVTVTVSDLNADLQAEVDESYTLVLDSGSSTLAIT-------------- 168
ACI15900.1_NagA_T. maritima     ----------------------------------SPDVTYRYSRVAGKIMEIVGLN-------------- 109
AAK16587.1_CbsA_T. neapolitana  ----------------------------------DPVFTGRYCEMAGRIMNTLGFN-------------- 109
CAA63123.1_HexB_S. scrofa       --------------------------ESECDTFPSISSNESYVLHVKGPEALLRAN-------------- 137
```

```
                                       570       580       590       600       610       620       630
                                   ....|....|....|....|....|....|....|....|....|....|....|....|....|....|
WP_082768773.1_Nah20B_P. hydro     ------------DNAGLFYGLMSLYQLLDGNNS--------------LPVGNITDAPRYE---------- 349
WP_068373836.1_Nah20A_P. hydro     ------------TERGMLHGVQTLLQLVNRTQRS-------------IPAVDIDDKPRFA---------- 162
BAD00143.1_Nah20A_A. hydrophil     ------------DVDGAFWGAQSLISLLGVDDK-------------LVSQMSVEDAPRFE---------- 334
BAF76001.1_NahB_A. hydrophila      ------------GEAGWRHGLVSLAQWALQHGES-------------LPCLTVRDAPRFG---------- 243
BAI94822.1_BbhI_B. bifidum        ------------SVTGNMYAMQTILQMTKQDANG-------------FVIGSMRDYPRFT---------- 567
AAQ05800.1_Hex20A_C. fimi          ------------SAAGLVRAVVTLRQTVS------SL---GDGT-LTVPALRVEDHPRYA---------- 136
BAI63641.1_Hex1t_Paenibacillus     ------------KPEGVFRGNQTLLQLLPAGIEKNTV---VSGVQWVIPHSNISDKPEYE---------- 160
CAA55582.1_NahA_P. gingivalis      ------------SAHGAFYGMQTLLQLLPAEVESSNE---V-LLPMTVPGVEIKDEPAFG---------- 171
BAA84281.1_Hex99_P. piscicida      -----------SDAQGVFYGIQTLRQMIPTTVYKTAKNSATLSDNAVLPASTILDAPRFE---------- 357
BAB84321.1_Hex86_P. piscicida      ------------TATGLFWGMQSLRQLLPAEIESRMP---INQASWAIPAVEIKDQPRFS---------- 164
AAB03808.1_Chb_S. marcescens       ------------DQAGVFYGLQSILSLVPSDGSG------------KIATLDASDAPRFP---------- 339
AAS02154.1_GcnA_S. gordonii        -------------YFGYFRGRYTVAELQEIEDYAAD--------FDMSFVPCIQTLAHLS---------- 172
AAK98861.1_StrH_S. pneumoniae      EVPKDLNRYTAESVAAVKEAEKAIRSLDSNLSRAQQDTIDQAIAKLQETVNNLTLTPEAQKEEEAKREVE 627
CAB87223.1_HexA_S. coelicolor      ------------KAAGLFHGVQTLRQLLPAAVEKDSA---QPGP-WLVAGGTIEDTPRYA---------- 181
AAC38798.3_SpHex_S. plicatus       ------------KAAGLFHGVQTLRQLLPPAVEKDSA---QPGP-WLVAGGTIEDTPRYA---------- 152
AKC34128.1_Hex1_uncultured Act     ------------RPAGLFRGVQTIRQLLPAAVERRTV---APGP-WTLPTGVLRDAPRFA---------- 145
AKC34129.1_Hex2_uncultured Bac     ------------DAAGVFLGTRTLLQLLPAKLAEG---------SAAIPAVTITDYPRFA---------- 153
AAC44672.1_ExoI_V. furnissii       ------------SHIGFVHASATLLQLVRPDGDNL-----------LVPHIVIKDAPRFK---------- 261
AAA88682.1_Chb_V. harveyi          ------------DQAGAFYAVQSIFGLVDSQNAD------------SLPQLSIKDAPRFD---------- 333
AAD30612.1_AtHex1_A. thaliana      ----------AHSAWGAMRGLETFSQMIWGTSPD---------LCLPVG-IYIQDSPLFG---------- 172
AAM91092.1_AtHex3_A. thaliana      ---------EAKSVYGALHGLQTFSQLCHFNLKKKV-------IEILMTPWNIIDQPRFS---------- 183
BAE99290.1_AtHex3_A. thaliana      ---------EANTVYGALRGLETFSQLCAFDYITKS-------VQIYKAPWYIQGKPRFG---------- 186
AAM13977.1_HexA_A. oryzae          ----------AETVWGALHAFTTLQQLVISDGHGG--------LIIEEP-VNIKDSPLYP---------- 183
AAT99455.1_BmGlcNAcase3_B. mor     ------------SIWGILRGLESWTHLFHLSDNRD--------QLHINK-GEVHDFPRYA---------- 183
AAC60521.1_BmChiGlcNAcase_B. m     ----------GNSFFGVRNGLETLSQLIVYDDIRNN-------LLIVRD-VTIKDRPVYP---------- 212
CCD73477.2_CeHEX-4_C. elegans      -----------LEVIPLIQSFGHLEFVLKKSKFMG---------------LSEDLIDLN---------- 155
AAA34346.2_Hex1_C. albicans       ----------AATTWGALHGLVSLQQLIIHTSEDK--------YVVPSS-VTISDFPNFK---------- 167
ADU20405.1_βHex_C. annuum          ----------AGTVWGAMRGLETFSQLVYGNP-----------TRVAAG-VYISDLPIFT---------- 167
ABB18373.1_CpHEX1_C. posadasii     ----------AKTTWGVLRAFTTLQQIVIFKRGR---------FLVEQP-VDIKDYPLYP---------- 178
AAA33230.1_NagA_D. discoideum      ------------NIYGAMRGLETFKQLIVYNELE---------NSYSIVCVSISDSPRYP---------- 156
AAF46406.1_Hex2_D. melanogaste     ----------ATTVYGARHAFETLSNLVTG-SLSNG-------LLMVTT-ANITDRPAFS---------- 236
AAF47881.1_Hex1_D. melanogaste     ----------ASNFFGARHGLETLAQLIVYDDIRRE-------VQVTAN-ATINDAPVYK---------- 215
AAM29423.1_Fdl_D. melanogaster     ----------ANSYFGARHGLSTLQQLIWFDDEDHL-------LHTYAN-SKVKDAPKFR---------- 277
CAE46968.1_HexA_E. histolytica     ------------TVYGARHAFETLLQLIRISSNK---------FVISQLPIKISDAPRFK---------- 184
AAB00965.1_HexA_H. sapiens        ------------TVWGALRGLETFSQLVWKSAEG---------TFFINK-TEIEDFPRFP---------- 168
AAA52645.1_HexB_H. sapiens        ------------RVWGALRGLETFSQLVYQDSYG---------TFTINE-STIIDSPRFS---------- 201
AAC53246.1_HexA_M. musculus       ------------TVWGALRGLETFSQLVWKSAEG---------TFFINK-TKIKDFPRFP---------- 168
AAA18776.1_HexB_M. musculus       ------------SVWGALRGLETFSQLVYQDSFG---------TFTINE-SSIADSPRFP---------- 180
ABI81756.1_Hex1_O. furnacalis     ----------ANSFFGMRHGLETLSQLFVFDDIRDH-------LLMVRD-VNISDKPVYP---------- 210
AEQ33603.1_HexTF_T. flavus        ----------ANTTWGALHAFTTLQQLVIYQDNS---------LIIEQP-VHIEDSPLYP---------- 208
ACI15900.1_NagA_T. maritima       -----------------MVFAPVLDILSEESSS-----------------VIDIRSYG---------- 133
AAK16587.1_CbsA_T. neapolitana    -----------------MVFAPVLDILSEKGSA-----------------VVDLRSFG---------- 133
CAA63123.1_HexB_S. scrofa         ------------TVWGALRGLETFSQLIYQDSYG---------TFTVNE-SEIIDFPRFP---------- 175
```

```
                                        640       650       660       670       680       690       700
                                ....|....|....|....|....|....|....|....|....|....|....|....|....|....|
WP_082768773.1_Nah20B_P. hydro  ----FRGLHVDVARNFHSKAFIINLLEQMAALKINKLHFHLAEDEAWRLEI----------PGLPELTDI 405
WP_068373836.1_Nah20A_P. hydro  ----WRGLLIDSVRHFMPVDTIKRQLRGMASAKLNVLHWHLTDDQGWRIAS----------KAYPKLQLL 218
BAD00143.1_Nah20A_A. hydrophil  ----YRGMQTDVARHFRSPETMKKLVDQMSAMKLNVLHLGLTNDEGWRLEI----------PGLPELTDV 390
BAF76001.1_NahB_A. hydrophila  ----FRGIFLDCARHFHSIATLKRLLKQMSLYKFNRFHWHLTDDEGWRLEI----------KTFPQLTAV 299
BAI94822.1_BbhI_B. bifidum     ----TRGLLLDVARKPVSLEMMREITRTMRYYKMNDFQAHLSDNYIFLENYGKGDNEDEAFKAYDAFRLE 633
AAQ05800.1_Hex20A_C. fimi      ----WRGLSIDVARHFFTVDDLKAIIGLLAHYKLNVLHLHLTDDQGWRVHL----------PSRPHLTRA 192
BAI63641.1_Hex1t_Paenibacillus ----YRGLMLDVARHFFTVDEVKRQIDLASQYKINKFHMHLSDDQGWRIEI----------KSWPDLIEI 216
CAA55582.1_NahA_P. gingivalis  ----YRGFMLDVCRHFLSVEDIKKHIDIMAMFKINRFHWHLTEDQAWRIEI----------KKYPRLTEV 227
BAA84281.1_Hex99_P. piscicida  ----YRGMMLDVSRNFQSKETVFKLLDLMAFYKLNKFEINLANDEGWRLEI----------PGIPELTEF 413
BAB84321.1_Hex86_P. piscicida  ----YRGMHLDVSRHFFDVAFVKRYIDWLAMHKFNVFQWHLTDDQGWRIAI----------DAYPKLTEI 220
AAB03808.1_Chb_S. marcescens   ----YRGIFLDVARNFHKKDAVLRLLDQMAAYKLNKFHFHLSDDEGWRIEI----------PGLPELTEV 395
AAS02154.1_GcnA_S. gordonii    ---AFVKWGIKEVQELRDVEDILLIGEEKVYDLIEGMFQTMAHLHTRKINIG---------MDEAHLVGL 230
AAK98861.1_StrH_S. pneumoniae  KLAKNKVISIDAGRKYFTLDQLKRIVDKASELGYSDVHLLLGNDGLRFLLNDMTITANGKTYASDDVKKA 697
CAB87223.1_HexA_S. coelicolor  ----WRSAMLDVSRHFFSVDEVKRYIDRVALYKYNKLHLHISDDQGWRLAI----------DSWPRLATY 237
AAC38798.3_SpHex_S. plicatus   ----WRSAMLDVSRHFFGVDEVKRYIDRVARYKYNKLHLHLSDDQGWRIAI----------DSWPRLATY 208
AKC34128.1_Hex1_uncultured Act ----WRGAMLDVARHFFPVADVKRYLDLLAYYKFNVLHLHLTDDQGWRIEI----------KSWPRLAEY 201
AKC34129.1_Hex2_uncultured Bac ----WRGMMLDVGRHFHPVPDIKRFIDWMAFHKLNSFHWHLTEDQGWRIEI----------KKYPKLTEV 209
AAC44672.1_ExoI_V. furnissii   ----YRGMMLDCARHFHPLERVKRLINQLAHYKFNTFHWHLTDDEGWRIEI----------KSLPQLTDI 317
AAA88682.1_Chb_V. harveyi      ----YRGVMVDVARNFHSKDAILATLDQMAAYKMNKLHLHLTDDEGWRLEI----------PGLPELTEV 389
AAD30612.1_AtHex1_A. thaliana  ----HRGVLLDTSRNYYGVDDIMRTIKAMSANKLNVFHWHITDSQSFPLVL----------PSEPSLAAK 228
AAM91092.1_AtHex3_A. thaliana  ----YRGLLIDTSRHYLPLPVIKNVIDSMTYAKLNVLHWHIVDTQSFPLEI----------PSYPKLWNG 239
BAE99290.1_AtHex3_A. thaliana  ----YRGLLIDTSRHYLPIDVIKQIIESMSFAKLNVLHWHIVDEQSFPLET----------PTYPNLWKG 242
AAM13977.1_HexA_A. oryzae      ----YRGIMLDTGRNFVSLPKIFEQLEGMSLSKLNVLHWHIDDAQSWPIWV----------DVYPEMVKD 239
AAT99455.1_BmGlcNAcase3_B. mor ----HRGLLVDTSRHYISMSNILLILDAMAMNKMNVFHWHIVDDQSFPYQS----------ERFPDLSRL 239
AAC60521.1_BmChiGlcNAcase_B. m ----YRGILLDTARNFYSIDSIKRTIDAMAAVKLNTFHWHITDSQSFPLVL----------QKRPNLSKL 268
CCD73477.2_CeHEX-4_C. elegans  ------TICISDSK---SIDIVKQMIEQIRRLHPNSTRIHIGADEAYHVAED---------QRCIERMEK 207
AAA34346.2_Hex1_C. albicans    ----HRGLMIDSGRNFLTVDSILEQIDIMALSKMNSLHWHLADSQSWPVAL----------ESYPHMIKD 223
ADU20405.1_βHex_C. annuum      ----HRGVMLDTSRNFYGVDDLLRLIKAMSMNKLNVFHWHITDSHSFPLVV----------PSEPELAGK 223
ABB18373.1_CpHEX1_C. posadasii ----VRGIMIDTARNFISVKKIFEQLDGMALSKLNVLHWHITDTQSWPVEV----------RSYPQMTED 234
AAA33230.1_NagA_D. discoideum  ----WRGFMVDSARHYIPKNMILHMIDSLGFSKFNTLHWHMVDAVAFPVES----------TTYPDLTKG 212
AAF46406.1_Hex2_D. melanogaste ----HRGVLLDTARNFVPLKFIRSTLDAMAASKLNVLHWHVVDTHSFPLEI----------TRVPEMQRY 292
AAF47881.1_Hex1_D. melanogaste ----WRGLLLDTSRNYYSVKSIKRTLEGMALVKLNTFHWHITDSHSFPLEV----------KKRPELHKL 271
AAM29423.1_Fdl_D. melanogaster ----YRGLMLDTSRHFFSVESIKRTIVGMGLAKMNRFHWHLTDAQSFPYIS----------RYYPELAVH 333
CAE46968.1_HexA_E. histolytica ----WRGLMVDPSRNPLSPLMFKRIIDTLASVKANVLHIHLSDAQTFVFES----------KKYPLLHQK 240
AAB00965.1_HexA_H. sapiens     ----HRGLLLDTSRHYLPLSSILDTLDVMAYNKLNVFHWHLVDDPSFPYES----------FTFPELMRK 224
AAA52645.1_HexB_H. sapiens     ----HRGILIDTSRHYLPVKIILKTLDAMAFNKFNVLHWHIVDDQSFPYQS----------ITFPELSNK 257
AAC53246.1_HexA_M. musculus    ----HRGVLLDTSRHYLPLSSILDTLDVMAYNKFNVFHWHLVDDSSFPYES----------FTFPELTRK 224
AAA18776.1_HexB_M. musculus    ----HRGILIDTSRHFLPVKTILKTLDAMAFNKFNVLHWHIVDDQSFPYQS----------TTFPELSNK 236
ABI81756.1_Hex1_O. furnacalis  ----YRGILLDTARNYYSIESIKRTIEAMAAVKLNTFHWHITDSQSFPFVT----------TKRPNLYKF 266
AEQ33603.1_HexTF_T. flavus     ----WRGVMIDTGRNFITVPKIKEQIDGMALSKLNILHWHLDDSQSWPVHM----------DTYPQMTND 264
ACI15900.1_NagA_T. maritima    ----SDPKIVAEHGARACEGYLEGGVIPCIKHFPGHGKAREDSHLTLPVVD----------APFEKLWEE 189
AAK16587.1_CbsA_T. neapolitana ----SDPEVVASHGMEACMGYFKGGVIPCIKHFPGHGKTADDSHYLLPTVN----------ASFEELWRE 189
CAA63123.1_HexB_S. scrofa      ----HRGILIDTGRHFLSVKTIFKTLDAMAFNKFNVLHWHIVDDQSFPYQS----------INFGVLSSK 231
                                             *
```

```
                                       710       720       730       740       750       760       770
                                  ....|....|....|....|....|....|....|....|....|....|....|....|....|....|
WP_082768773.1_Nah20B_P. hydro    GGFRCFDPLEQTCLMPQLGSGPERK----------------------------------TKVNGYLTTQD 441
WP_068373836.1_Nah20A_P. hydro    ASDG-----------------------------------------------------------LFYTQQQ 229
BAD00143.1_Nah20A_A. hydrophil    GSQRCHDESETKCLMPQLGSGPTSD----------------------------------NQGSGFYSKAD 426
BAF76001.1_NahB_A. hydrophila     GAWRGHG----LAVGPQLSGGP-------------------------------------DPYGGYYTQSE 328
BAI94822.1_BbhI_B. bifidum        SSLTNDKGES------------------------------------------------PTAEDYSISKKT 655
AAQ05800.1_Hex20A_C. fimi         SAGT--------------SVGGG--------------------------------------PGGFYNPAQ 210
BAI63641.1_Hex1t_Paenibacillus    GSKG--------------QVGGG--------------------------------------PGGYYTQEQ 234
CAA55582.1_NahA_P. gingivalis     GSTR------------TEGDGTQ--------------------------------------YSGFYTQEQ 247
BAA84281.1_Hex99_P. piscicida     GAKRGYDLSESTMLHTFMGAANGFAPGDGIENKPTDETAANLGTAPSYQGFEIAQQNFLGKGWGYYTVND 483
BAB84321.1_Hex86_P. piscicida     GATRPHTVVGHTYDYQPLFDNKT--------------------------------------VSGFYTKAQ 252
AAB03808.1_Chb_S. marcescens      GGQRCHDLSETTCLLPQYGQGP--D----------------------------------VYG-GFFSRQD 428
AAS02154.1_GcnA_S. gordonii       GRYLIKHG---------------------------------------------------FQNRSLLMCQH 249
AAK98861.1_StrH_S. pneumoniae     IIEGTKAYYD-------------------------------------------------DPNGTALTQAE 718
CAB87223.1_HexA_S. coelicolor     GGST--------------EVGGG--------------------------------------PGGHYTKAD 255
AAC38798.3_SpHex_S. plicatus      GGST--------------EVGGG--------------------------------------PGGYYTKAE 226
AKC34128.1_Hex1_uncultured Act    GGST--------------AVDGA--------------------------------------PGGYYTQEQ 219
AKC34129.1_Hex2_uncultured Bac    GAFRESSPP---YGNRNSDDGVR--------------------------------------YGGFYTQEQ 238
AAC44672.1_ExoI_V. furnissii      GAWRGVD----EVLEPQYSLLT-------------------------------------EKHGGFYTQEE 346
AAA88682.1_Chb_V. harveyi         GANRCFDTQEKSCLLPQLGSGPTTD----------------------------------NFGSGYFSKAD 425
AAD30612.1_AtHex1_A. thaliana     GSLG---------------------------------------------------------PDMVYTPED 241
AAM91092.1_AtHex3_A. thaliana     AYSS----------------------------------------------------------SQRYTFED 251
BAE99290.1_AtHex3_A. thaliana     AYSR----------------------------------------------------------WERYTVED 254
AAM13977.1_HexA_A. oryzae         -AYS---------------------------------------------------------PHEIYSRND 251
AAT99455.1_BmGlcNAcase3_B. mor    GAYH---------------------------------------------------------ETLIYTKEN 252
AAC60521.1_BmChiGlcNAcase_B. m    GAYS---------------------------------------------------------PTKVYTKQD 281
CCD73477.2_CeHEX-4_C. elegans     ESIG----------------------------------------------------------KSDLKLEH 219
AAA34346.2_Hex1_C. albicans       -AYS---------------------------------------------------------NDEVYSKND 235
ADU20405.1_βHex_C. annuum         GAYG---------------------------------------------------------NEMMYSPAD 236
ABB18373.1_CpHEX1_C. posadasii    -AYS---------------------------------------------------------RRETYGPSD 246
AAA33230.1_NagA_D. discoideum     -AFS---------------------------------------------------------PSATFSHDD 224
AAF46406.1_Hex2_D. melanogaste    GAYS---------------------------------------------------------SSQTYSRQD 305
AAF47881.1_Hex1_D. melanogaste    GAYS---------------------------------------------------------QRQVYTRRD 284
AAM29423.1_Fdl_D. melanogaster    GAYS---------------------------------------------------------ESETYSEQD 346
CAE46968.1_HexA_E. histolytica    GMYD---------------------------------------------------------ESFVLTQSF 253
AAB00965.1_HexA_H. sapiens        GSYNP--------------------------------------------------------VTHIYTAQD 238
AAA52645.1_HexB_H. sapiens        GSYS---------------------------------------------------------LSHVYTPND 270
AAC53246.1_HexA_M. musculus       GSFNP--------------------------------------------------------VTHIYTAQD 238
AAA18776.1_HexB_M. musculus       GSYS---------------------------------------------------------LSHVYTPND 249
ABI81756.1_Hex1_O. furnacalis     GALS---------------------------------------------------------PQKVYTKAA 279
AEQ33603.1_HexTF_T. flavus        -AYS---------------------------------------------------------PWQTYSHED 276
ACI15900.1_NagA_T. maritima       DLLP--------------------------------------------------------FRKVLEREKK 203
AAK16587.1_CbsA_T. neapolitana    DLLP--------------------------------------------------------FRRIFQSRVK 203
CAA63123.1_HexB_S. scrofa         GSYS---------------------------------------------------------LSHVYTPND 244
```

```
                                           780       790       800       810       820       830       840
                                       ....|....|....|....|....|....|....|....|....|....|....|....|....|....|
WP_082768773.1_Nah20B_P. hydro         YIDILRFADAHF--IEVIPSLDMPGHSRAAIKSMEARYKRLVSEEK-------------------TPEAE 490
WP_068373836.1_Nah20A_P. hydro         IREVVQYAALLG--IRVVPEFDVPGHASAIAVAYPELITQP----------------------------- 268
BAD00143.1_Nah20A_A. hydrophil         YIDLVRYAKARG--VTVIPEINMPAHARAAVVSMEARYKRLMSEGK-------------------ETEAN 475
BAF76001.1_NahB_A. hydrophila          VRELVAYAAGLG--ITIIPEIDIPGHCHAAIKALPE---------------------------------- 362
BAI94822.1_BbhI_B. bifidum             FKQFIQDERALG--MNVVPEIDVPAHANSFTKIWPELMVKGRVSPINSNRPLIDHLDVSKPETIAKIKEI 723
AAQ05800.1_Hex20A_C. fimi              LAEIVVARAARG--IRVVPEIDVPGHVNAATHAYGDLT-------------------------------- 246
BAI63641.1_Hex1t_Paenibacillus         FKDIVSYAAERY--IEVIPEIDMPGHTNAALASYGELN-------------------------------- 270
CAA55582.1_NahA_P. gingivalis          VRDIVQYASDHF--ITVIPMIEMPGHAMAALAAYPQFR-------------------------------- 283
BAA84281.1_Hex99_P. piscicida          FKEILQYAADRH--IDVIIEYDFPAHARAAIKAMEHRYNKYKDS---------------------DPVEA 530
BAB84321.1_Hex86_P. piscicida          IKEVIEYAQARH--IEVIPEIDIPGHSSAMLAAYPELS-------------------------------- 288
AAB03808.1_Chb_S. marcescens           YIDIIKYAQARQ--IEVIPEIDMPAHARAAVVSMEARYKKLHAAGK-------------------EQEAN 477
AAS02154.1_GcnA_S. gordonii            LERVLDIADKYG--FNCQMWSDMFFKLMSADGQYDRDVEIP----------------------------- 288
AAK98861.1_StrH_S. pneumoniae         VTELIEYAKSKD--IGLIPAINSPGHMDAMLVAMEKLGIKN----------------------------- 757
CAB87223.1_HexA_S. coelicolor          YEEIVRYAASRH--LEVVPEIDMPGHTNAALASYAELN-------------------------------- 291
AAC38798.3_SpHex_S. plicatus           YKEIVRYAASRH--LEVVPEIDMPGHTNAALASYAELN-------------------------------- 262
AKC34128.1_Hex1_uncultured Act         YADLARYAAERY--ITIVPEIDMPSHTNAALASYALLN-------------------------------- 255
AKC34129.1_Hex2_uncultured Bac         LKDVVAYAAARH--ITVVPEIEMPGHAAAAIAAYPELG-------------------------------- 274
AAC44672.1_ExoI_V. furnissii           IREVIAYAAERG--ITVIPEIDIPGHSRAAIKALPE---------------------------------- 380
AAA88682.1_Chb_V. harveyi              YVEILKYAKARN--IEVIPEIDMPAHARAAVVSMEARYDRLMEEGK-------------------EAEAN 474
AAD30612.1_AtHex1_A. thaliana          VSKIVQYGFEHG--VRVLPEIDTPGHTGS-WGEAYPEIVTC----------------------------- 279
AAM91092.1_AtHex3_A. thaliana          AAEIVNYARRRG--IHVLAEIDVPGHALSWGKGYPALWPS------------------------------ 289
BAE99290.1_AtHex3_A. thaliana          ASEIVRFAKMRG--INVMAEVDVPGHAESWGTGYPDLWPS------------------------------ 292
AAM13977.1_HexA_A. oryzae              VRNIVNYARARG--IRVIPEIDMPSHSSSGWKQVDPEMVTC----------------------------- 290
AAT99455.1_BmGlcNAcase3_B. mor         IQTVIDHARNRG--IRVIPEFDVPGHTRSWGVAKPDLLTHC----------------------------- 291
AAC60521.1_BmChiGlcNAcase_B. m         IREVVEYGLERG--VRVLPEFDAPAHVGEGWQDTG----------------------------------- 314
CCD73477.2_CeHEX-4_C. elegans          IAKIGKFARENAGFETVFAWNDMFDKESEETIRKSKIN-------------------------------- 257
AAA34346.2_Hex1_C. albicans            LKYIVDYARARG--VRVIPEIDMPGHARAGWKQVDPTIVEC----------------------------- 274
ADU20405.1_βHex_C. annuum              VEKIVEFGMEHG--VRVLPEIDMPAHTGS-WAEAYPEIITC----------------------------- 274
ABB18373.1_CpHEX1_C. posadasii         IRKVIEYARARG--IRVVPEIDMPGHSASGWRKIDPDIVAC----------------------------- 285
AAA33230.1_NagA_D. discoideum          IQEVVAYAKTYG--IRVIPEFDIPGHAAAWGIGYPELVATC----------------------------- 263
AAF46406.1_Hex2_D. melanogaste         ALNLVKYARLRG--IRILIEIDGPSHAGNGWQWGPAAGLGN----------------------------- 344
AAF47881.1_Hex1_D. melanogaste         VAEVVEYGRVRG--IRVMPEFDAPAHVGEGWQHKN----------------------------------- 317
AAM29423.1_Fdl_D. melanogaster         VREVAEFAKIYG--VQVIPEIDAPAHAGNGWDWGPKRGMGE----------------------------- 385
CAE46968.1_HexA_E. histolytica         LRELAQYGANRG--VIVYGEIDTPAHTASWNLGYPGVVANC----------------------------- 292
AAB00965.1_HexA_H. sapiens             VKEVIEYARLRG--IRVLAEFDTPGHTLSWGPGIPGLLTPC----------------------------- 277
AAA52645.1_HexB_H. sapiens             VRMVIEYARLRG--IRVLPEFDTPGHTLSWGKGQKDLLTPC----------------------------- 309
AAC53246.1_HexA_M. musculus            VKEVIEYARLRG--IRVLAEFDTPGHTLSWGPGAPGLLTPC----------------------------- 277
AAA18776.1_HexB_M. musculus            VRMVLEYARLRG--IRVIPEFDTPGHTQSWGKGQKNLLTPC----------------------------- 288
ABI81756.1_Hex1_O. furnacalis          IREVVRFGLERG--VRVLPEFDAPAHVGEGWQDTD----------------------------------- 312
AEQ33603.1_HexTF_T. flavus             IKDIIEYARARA--VRVVPEVDMPGHSAAGWQQVDPSIVAC----------------------------- 315
ACI15900.1_NagA_T. maritima            VTVMTAHVRYSS--IDSLPATLSEKIITDVLREKIG---------------------------------- 237
AAK16587.1_CbsA_T. neapolitana         TAVMTAHVKYPA--VDDLPATLSKKLITEVLREKLN---------------------------------- 237
CAA63123.1_HexB_S. scrofa              VRMVIEYARIRG--IRVMPEFDTPGHSRSWGKGQKDLLTPC----------------------------- 283
                                                            *
```

```
                                   850       860       870       880       890       900       910
                               ....|....|....|....|....|....|....|....|....|....|....|....|....|
WP_082768773.1_Nah20B_P. hydro QYLLTEFADKSQYRSIQHYDDN-TLNPCLDSTYHFVDKVLSELIKMHNKAGVA-LKRYHIGADETAGAWQ 558
WP_068373836.1_Nah20A_P. hydro ---------GPYTMQRHWGVFEPLLDPSNPQVYQFIETIISELAELFP------DVYVHIGGDEIEPTQW 323
BAD00143.1_Nah20A_A. hydrophil QFRLTDPADTSNVTSVQFYDKMSFINPCQPGAATFVAKVMDEVAQMHQAAGQP-LTAWHYGGDEAKNIMQ 544
BAF76001.1_NahB_A. hydrophila  --LLVEEADRSRYLSVQYFDDN-VLNPALPGTYRFLDAVMDEVCDLFPGS------QVHMGGDEVP---- 419
BAI94822.1_BbhI_B. bifidum    FDDYTKGDDPTFDSDTTVHIGADEFLYNYTAYRKFINEIVPYIKDTNTVRMWGGLTWINDHKTEITKDAI 793
AAQ05800.1_Hex20A_C. fimi      ---PSGE---PTDVYTGIEVGFSRLHDDLPATRPFLRDVFTDLAAMTPGE------YVHIGGDEV----- 299
BAI63641.1_Hex1t_Paenibacillus ---PDGK---RKAMRTDTAVGYSTLMPRAEITYQFVEDVISELAAISPSP------YIHLGGDES----- 323
CAA55582.1_NahA_P. gingivalis  ---CFPR-EFKPRII--WGVEQDVYCAGKDSVFRFISDVIDEVAPLFPGT------YFHIGGDECPKDRW 341
BAA84281.1_Hex99_P. piscicida  NRFRLIDPLDQSQYYTPQFYTDNFVNPALESTFTFLNHVVRETRAIYDAVPDATLTRLHGGGDELPHLGP 600
BAB84321.1_Hex86_P. piscicida  ---CHQR---AVKVQPQFGIFEDVLCP-REDVFAFLGVVYKEVAELFPSQ------YIHIGGDEVIKKQW 345
AAB03808.1_Chb_S. marcescens   EFRLVDPTDTSNTTSVQFFNRQSYLNPCLDSSQRFVDKVIGEIAQMHKEAGQP-IKTWHFGGDEAKNIRL 546
AAS02154.1_GcnA_S. gordonii    ------------------EETRVYLDRLKERVTLVYWDYYQDSEEKYNRN---FQNHHKISQDIAFAGG- 336
AAK98861.1_StrH_S. pneumoniae  ------------PQAHFDKVSKTTMDLKNEEAMNFVKALIGKYMDFFAG----KTKIFNFGTDEYANDAT 811
CAB87223.1_HexA_S. coelicolor  ---CDGV---APPLYTGTKVGFSTLCVDKDVTYDFVDDVLGELAALTPGR------YLHIGGDEA----- 344
AAC38798.3_SpHex_S. plicatus   ---CDGV---APPLYTGTKVGFSSLCVDKDVTYDFVDDVIGELAALTPGR------YLHIGGDEA----- 315
AKC34128.1_Hex1_uncultured Act ---CEGE---APALYTGTAVGFSTLCMGKETTFRFMDDVLGELAALTPGP------YLHIGGDEA----- 308
AKC34129.1_Hex2_uncultured Bac ---NTDIPGYSPKVMTRWGVHPYIFSP-KEETFTFLEDVLSEVCDLFPSK------YIHIGGDEAPKDQW 334
AAC44672.1_ExoI_V. furnissii   --WLFDEDDQSQYRSIQYYNDN-VLSPALPGTYRFLDCVLEEVAALFPSH------FIHIGADEVP---- 437
AAA88682.1_Chb_V. harveyi      EYRLMDPQDTSNVTTVQFYNKQSFINPCMESSTRFVDKVISEVAAMHQEAGAP-LTTWHFGGDEAKNIKL 543
AAD30612.1_AtHex1_A. thaliana  ANMFWWPAGKSWEERLASEPGTGQLNPLSPKTYEVVKNVIQDIVNQFP------ESFFHGGGDEVIPGCW 343
AAM91092.1_AtHex3_A. thaliana  ------------------KNCQEPLDVSSDFTFKVIDGILSDFSKIFK------FKFVHLGGDEVNTTCW 335
BAE99290.1_AtHex3_A. thaliana  ------------------LSCREPLDVTKNFTFDVISGILADMRKIFP------FELFHLGGDEVNTDCW 338
AAM13977.1_HexA_A. oryzae      TDSWWSNDDWP--LHTAVEPNPGQLDIIYNKTYEVVGNVYKELSDIFP------DHWFHVGGDEIQPNCF 352
AAT99455.1_BmGlcNAcase3_B. mor YDQDGDY------------VGLGPMNPIKDSTYTFLQELFHEVQALFP------ERYIHIGGDEVDLDCW 343
AAC60521.1_BmChiGlcNAcase_B. m LTVCFKAEPWT---KFCVEPPCGQLNPTKEELYDYLEDIYVEMAEAFES-----TDMFHMGGDEVSERCW 376
CCD73477.2_CeHEX-4_C. elegans  ---------------KFIVPVVWGYRTDVTENGYFPDGLFERIFNVFDR-------FYVASAFKGADGAR 305
AAA34346.2_Hex1_C. albicans    ADAFWTD--------AAVEPPPGQLNIESEKTYEVISNVYNELSDIFI------DDVFHVGNDELQEKCY 330
ADU20405.1_βHex_C. annuum      ANMFWWPAGNS--PALAAEPGTGQLNPLIPKTYEVVKNVIHDTIAMFP------DSLFHGGADEINSACW 336
ABB18373.1_CpHEX1_C. posadasii ADSWWSNDDWE--KHTAVQPNPGQLDIANNKTYKVVEKVYNDISRIFT------DDWFHVGGDELQPNCF 347
AAA33230.1_NagA_D. discoideum  PDYAANVNN-------------IPLDISNPATFTFIQNLFTEIAPLFI------DNYFHTGGDELVTGCW 314
AAF46406.1_Hex2_D. melanogaste MSVCLNQSPWR---RFCVQPPCGQLNPLNDHMYAVLKEIFEDVAEVGAP-----EETLHMGGDEVFLPCW 406
AAF47881.1_Hex1_D. melanogaste MTACFNAQPWK---SFCVEPPCGQLDPTVNEMYDVLEDIYGTMFDQFN------PDIFHMGGDEVSTSCW 378
AAM29423.1_Fdl_D. melanogaster LAMCINQQPWS---FYCGEPPCGQLNPKNNYTYLILQRIYEELLQHTGP-----TDFFHLGGDEVNLDCW 447
CAE46968.1_HexA_E. histolytica WDYIVSTSMRY-------GENVLSLNPANPNTFPIIDALMKELSDTFG------TDYVHVGGDEVWTSGW 349
AAB00965.1_HexA_H. sapiens     YSGSEPS------------GTFGPVNPSLNNTYEFMSTFFLEVSSVFP------DFYLHLGGDEVDFTCW 329
AAA52645.1_HexB_H. sapiens     YSRQNKL------------DSFGPINPTLNTTYSFLTTFFKEISEVFP------DQFIHLGGDEVEFKCW 361
AAC53246.1_HexA_M. musculus    YSGSHLS------------GTFGPVNPSLNSTYDFMSTLFLEISSVFP------DFYLHLGGDEVDFTCW 329
AAA18776.1_HexB_M. musculus    YNQKTKT------------QVFGPVDPTVNTTYAFFNTFFKEISSVFP------DQFIHLGGDEVEFQCW 340
ABI81756.1_Hex1_O. furnacalis  LTVCFKAEPWK---SYCVEPPCGQLNPTKDELYQYLEDIYSDMAEVFDT-----TDIFHMGGDEVSEACW 374
AEQ33603.1_HexTF_T. flavus     AHSWWSNDNWP--YHTAVEPTPGQLDPLNNKTYGVVEKVYNELSGIFT------DDFFHVGGDELQTGCY 377
ACI15900.1_NagA_T. maritima    ---------------------FDGLVISDAMEMSAVSNNFSVEEIVSL--------FLNAGGNMILLGDY 278
AAK16587.1_CbsA_T. neapolitana ---------------------FKGLVLSDAMEMKAISENFSVEEAVRF--------FIEAGGNMILLDNF 278
CAA63123.1_HexB_S. scrofa      YRKQVLS------------GTFGPINPILNTTYNFLSKFFKEISTVFP------DEFIHIGGDEVDFDCW 335
                                                                                              *
```

```
                                        920       930       940       950       960       970       980
                                ....|....|....|....|....|....|....|....|....|....|....|....|....|....|
WP_082768773.1_Nah20B_P. hydro  G--------------------------------SPACQAMIAGNARLQSSADLGPYFVERVAQMVAAKGI 596
WP_068373836.1_Nah20A_P. hydro  QQSQ--------------------------------AVQHYMQQHQLADEHQLHAYFNQQVQQILSKYGK 361
BAD00143.1_Nah20A_A. hydrophil  GGGYQDPAVTKKEDLVAWKGNVDSSKQDKPFGKSPMCQKMID-DGKIKDVAELPVYFAKEVSEMVKGHGF 613
BAF76001.1_NahB_A. hydrophila   ---------------------------TGVWTDSPACQQLMA-EQGYQDCRELQGHLLRHCQHYLAGKGK 461
BAI94822.1_BbhI_B. bifidum      ENVEMNLWSKDWADGLQMYNMGYKLINTIDDYGYMVPNGSYGRANAYGDLLNISRVFDSFEPNKVRSSGG 863
AAQ05800.1_Hex20A_C. fimi       ----------------------------------------------LTMDHDKYARLVGYAASVVRDAGK 323
BAI63641.1_Hex1t_Paenibacillus  ----------------------------------------------NATSAADYDYFFGRVTAIANSYGK 347
CAA55582.1_NahA_P. gingivalis   KACSLC--------------------------------QKRMRDNGLKDEHELQSYFIKQAEKVLQKHGK 379
BAA84281.1_Hex99_P. piscicida   NEWWAQS-----------------------------PAVDQNPATAGKTDAELFDYFFLRWKQIVNQHGF 641
BAB84321.1_Hex86_P. piscicida   LESPEV--------------------------------KKLMQQHQLTTPEQVQSYFIKRVAKIVQNLGK 383
AAB03808.1_Chb_S. marcescens    GAGYTD---KAKPEPG--KGIIDQSNEDKPWAKSQVCQTMIK-EGKVADMEHLPSYFGQEVSKLVKAHGI 610
AAS02154.1_GcnA_S. gordonii     ----------------------------------------AWKWIGFTPHNHFSRLVAIEANKACRKNQV 366
AAK98861.1_StrH_S. pneumoniae   SAQG-------------------------------------WYYLKWYQLYGKFAEYANTLAAMAKERGL 844
CAB87223.1_HexA_S. coelicolor   ----------------------------------------------HSTPQADFVAFMKRVQPIVAKYGK 368
AAC38798.3_SpHex_S. plicatus    ----------------------------------------------HSTPKADFVAFMKRVQPIVAKYGK 339
AKC34128.1_Hex1_uncultured Act  ----------------------------------------------DSTSPEDYQTFMRQVQAIVQSHGK 332
AKC34129.1_Hex2_uncultured Bac  KQSKFA--------------------------------QEVIKREGLKNEEELQSWFIRRIGRFLESKNR 372
AAC44672.1_ExoI_V. furnissii    ---------------------------DGVWVNSPKCQALMA-EEGYTDAKELQGHLLRYAEKKLKSLGK 479
AAA88682.1_Chb_V. harveyi       GAGFQD---VNAEDKVSWKGTIDLSKQDKPFAQSPQCQTLIT-DGTVSDFAHLPSHFAEEVSKIVAEKGI 609
AAD30612.1_AtHex1_A. thaliana   KTDP--------------------------------AINSFLSSG--GTLSQLLEKYINSTLPYIVSQNR 379
AAM91092.1_AtHex3_A. thaliana   SATP--------------------------------RIAQWLKKHRMSEKEAYQYFVLRAQKIALSHGYE 373
BAE99290.1_AtHex3_A. thaliana   KNTT--------------------------------HVKERLQGRNFTTKDAYKYFVLRAQQIAISKNWT 376
AAM13977.1_HexA_A. oryzae       NFST--------------------------------HVTKWFAEDPSRTYHDLAQYWVDHAVPIFQNYSQ 390
AAT99455.1_BmGlcNAcase3_B. mor  ESNP--------------------------------EFQRYIQEHNL-TSVADFHALFMRNTIPLLSENS 380
AAC60521.1_BmChiGlcNAcase_B. m  NSSE--------------------------------EIQNFMIQNRWNLDKSSFLKLWNYFQKNAQDRAY 414
CCD73477.2_CeHEX-4_C. elegans   QQFS-----------------------------------------NISRYLENQKSYVNLMDLHKNAAAQ 334
AAA34346.2_Hex1_C. albicans     S----------------------------------------AQLSPNNTVTDLLKRYLKKALPIFNKVNH 360
ADU20405.1_βHex_C. annuum       NTDP--------------------------------SIQTFVASN--GTQSQLLEMFINNTLPEILSLNR 372
ABB18373.1_CpHEX1_C. posadasii  LTSK--------------------------------IVRDWLKQG-SRTFNDLLQHWVDKTVPMMKKVKK 384
AAA33230.1_NagA_D. discoideum   LEDP--------------------------------AIANWMTKMGFSTTDAFQ-YFENNLDVTMKSINR 351
AAF46406.1_Hex2_D. melanogaste  NNTD--------------------------------EIRDGMRARGYDLSEQSFLRLWSQFHQRNLNAWD 444
AAF47881.1_Hex1_D. melanogaste  NSSQ--------------------------------PIQQWMKKQGWGLETADFMRLWGHFQTEALGRVD 416
AAM29423.1_Fdl_D. melanogaster  AQY---------------------------------------------FNDTDLRGLWCDFMLQAMARLK 472
CAE46968.1_HexA_E. histolytica  SKSKEYS-----------------------------DIQKFMKSKGLNSLTELEGYFNKYAQEQVIHNGK 390
AAB00965.1_HexA_H. sapiens      KSNP--------------------------------EIQDFMRKKGFGEDFKQLESFYIQTLLDIVSSYG 367
AAA52645.1_HexB_H. sapiens      ESNP--------------------------------KIQDFMRQKGFGTDFKKLESFYIQKVLDIIATIN 399
AAC53246.1_HexA_M. musculus     KSNP--------------------------------NIQAFMKKKGF-TDFKQLESFYIQTLLDIVSDYD 366
AAA18776.1_HexB_M. musculus     ASNP--------------------------------NIQGFMKRKGFGSDFRRLESFYIKKILEIISSLK 378
ABI81756.1_Hex1_O. furnacalis   NSSD--------------------------------SIQNFMMQNRWDLDKESFLKLWNYFQQKAQDKAY 412
AEQ33603.1_HexTF_T. flavus      NFST--------------------------------YVSDYLAADPSRTYNDVTQYWVDHAFPIFKKIQN 415
ACI15900.1_NagA_T. maritima     RNLP--------------------------------------------VYYETLVKLLEDGKVQKDKVER 304
AAK16587.1_CbsA_T. neapolitana  RDLP--------------------------------------------VYYESLKKLIEDGSIERGKVER 304
CAA63123.1_HexB_S. scrofa       ASNS--------------------------------EILQFMQEKGFSQISLNSNLCTVFKISNMISAMK 373
```

```
                                          990       1000      1010      1020      1030      1040      1050
                                 ....|....|....|....|....|....|....|....|....|....|....|....|....|....|
WP_082768773.1_Nah20B_P. hydro   E--------------AAAWDDGLSHAKAD--Q---------LPAKVQANIWGLLPSNG------------ 629
WP_068373836.1_Nah20A_P. hydro   Q--------------MMGWDEVFATDLPKDIVV------QSWRGLDSLQHIASSDYQVLLSTGYYVDQPQ 411
BAD00143.1_Nah20A_A. hydrophil   ST-------------LQAWEDGLKYATDASVF---------ATDKTRVNFWETLYWGG------------ 649
BAF76001.1_NahB_A. hydrophila   Q--------------MLGWEEILHG-------------------DKVSREATVFAWTS------------ 486
BAI94822.1_BbhI_B. bifidum      YQAVPSGDDQMLGAAFAIWSDNIDKSASGLTESDLYWRFFDAMPFYAEKTWAATGKEKGTAAKLTALAAK 933
AAQ05800.1_Hex20A_C. fimi       K--------------VVGWQ-EISSTP--------------LEPGTVVQYWDINADPAP----------- 353
BAI63641.1_Hex1t_Paenibacillus  K--------------VVGWDPSDTSSG--------------ATSDSVLQNWTCSASTG------------ 377
CAA55582.1_NahA_P. gingivalis   R--------------LIGWD-EILEGG--------------LAPSATVMSWRGEDGG------------- 407
BAA84281.1_Hex99_P. piscicida   D--------------MATWGDVLSHNGTGNVSY------GEIYPVFWNNVWGWGNEH------------- 678
BAB84321.1_Hex86_P. piscicida   T--------------VIGWD-EILEGG--------------VADDAVIMSWRGTEGG------------- 411
AAB03808.1_Chb_S. marcescens    DR-------------MQAWQDGLKDAESSKAF---------ATSRVGVNFWDTLYWGG------------ 646
AAS02154.1_GcnA_S. gordonii     K-----------EVIVTGWGDNGGETSQFSVLP-------------ALQIWAELAYRN------------ 400
AAK98861.1_StrH_S. pneumoniae   QP--------------MAFNDGFYYEDKDDVQFD---------KDVLISYWSKGWWGYN----------- 880
CAB87223.1_HexA_S. coelicolor   T--------------VVGWH-QLAGAE--------------PVEGALVQYWGLDRTSDAE---------- 399
AAC38798.3_SpHex_S. plicatus    T--------------VVGWH-QLAGAE--------------PVEGALVQYWGLDRTGDAE---------- 370
AKC34128.1_Hex1_uncultured Act  T--------------VVGWG-EIAQAP--------------LTPGTMVQHWRQDLT-------------- 359
AKC34129.1_Hex2_uncultured Bac  N--------------LIGWD-EIQEGG--------------LPKTATMMVWRDAKWA------------- 400
AAC44672.1_ExoI_V. furnissii    R--------------MVGWEEAQHG-------------------DKVSKDTVIYSWLS------------ 504
AAA88682.1_Chb_V. harveyi       PN-------------FQAWQDGLKYSDGEKAF---------ATENTRVNFWDVLYWGG------------ 645
AAD30612.1_AtHex1_A. thaliana   --------------TVVYWEDVLLDAQIKADPS------VLPKEHTILQTWNNGPEN------------- 416
AAM91092.1_AtHex3_A. thaliana   I---------------INWEETFINFGSKLNR------------KTVVHNWLNTG--------------- 401
BAE99290.1_AtHex3_A. thaliana   P---------------VNWEETFSSFGKDLDP------------RTVIQNWLVSD--------------- 404
AAM13977.1_HexA_A. oryzae       E------------RRLVMWEDIALS-ADNAH--------DVP-KNIVMQSWNNGLEY------------- 425
AAT99455.1_BmGlcNAcase3_B. mor  R--------------PIVWQEVFDEGVPLPK-------------DTIVQVWKENE--------------- 408
AAC60521.1_BmChiGlcNAcase_B. m  K-AFGKR------LPLILWTSTLTDYTHVEKFL------DKD--EYIIQVWTTGADP------------- 456
CCD73477.2_CeHEX-4_C. elegans   KVDG---------IFVTGWSRFNHFN-------------------------------------------- 351
AAA34346.2_Hex1_C. albicans     -------------RKLTMWDDVLLS-DVSAD--------KIP-SNITLQVWH-EISG------------- 393
ADU20405.1_βHex_C. annuum       --------------TVVYWEDVILSANVKVDPS------LLSPQHVIMQTWNNGPSN------------- 409
ABB18373.1_CpHEX1_C. posadasii  N------------RRLLMWEDVLLSGNMHAH--------RVP-RDIIMQSWNGGLAN------------- 420
AAA33230.1_NagA_D. discoideum   TK--------------ITWNDPIDYGVQLNPE-------------TLVQVWSSGS--------------- 379
AAF46406.1_Hex2_D. melanogaste  EINERMYPGIKEPKSVIIWSSHLTNPRYIETYL------PKE--RFIIQTWVESQDA------------- 493
AAF47881.1_Hex1_D. melanogaste  KVANGTH------TPIILWTSGLTEEPFIDEYL------NPE--RYIIQIWTTGVDP------------- 459
AAM29423.1_Fdl_D. melanogaster  LANNGVAP-----KHVAVWSSALTN----TKRL------PNS--QFTVQVWGGSTWQ------------- 512
CAE46968.1_HexA_E. histolytica  HP--------------VVWEEVFKKG-NADKN-------------TIIQVWDDIR--------------- 417
AAB00965.1_HexA_H. sapiens      KG-------------YVVWQEVFDNKVKIQP-------------DTIIQVWREDIPVN-Y---------- 400
AAA52645.1_HexB_H. sapiens      KG-------------SIVWQEVFDDKAKLAP-------------GTIVEVWKDSA----Y---------- 429
AAC53246.1_HexA_M. musculus     KG-------------YVVWQEVFDNKVKVRP-------------DTIIQVWREEMPVE-Y---------- 399
AAA18776.1_HexB_M. musculus     KN-------------SIVWQEVFDDKVELQP-------------GTVVEVWKSEH----Y---------- 408
ABI81756.1_Hex1_O. furnacalis   K-AFGKK------LPLILWTSTLTNYKHIDDYL------NKD--DYIIQVWTTGVDP------------- 454
AEQ33603.1_HexTF_T. flavus      -------------RKLVIWEDLIIN-NPHAP--------NVSTENLLVQSWNNGLTN------------- 450
ACI15900.1_NagA_T. maritima     S--------------IRTVEKYLAFAKKNSGVG--------FLADVSMKAVEFLG--------------- 337
AAK16587.1_CbsA_T. neapolitana  S--------------IKIVDEYLSALENRFNSG--------LIAEVAERAIECTR--------------- 337
CAA63123.1_HexB_S. scrofa       KR-------------PIVWQEAFDGRDKFMP-------------GTVVQVWKIED----Y---------- 403
```

```
                                           1060      1070      1080      1090      1100      1110      1120
                                   ....|....|....|....|....|....|....|....|....|....|....|....|....|....|
WP_082768773.1_Nah20B_P. hydro     ---------------------------------------------------------------------- 629
WP_068373836.1_Nah20A_P. hydro     EAAYHYRNDPFTSFKQPSFKTQDIKHW------------------------------------------- 438
BAD00143.1_Nah20A_A. hydrophil     ---------------------------------------------------------------------- 649
BAF76001.1_NahB_A. hydrophila      ---------------------------------------------------------------------- 486
BAI94822.1_BbhI_B. bifidum         QGTGPRTNPYYQATSKNSVYESYDFNDGLADASGNGRDLTIGDGSKAAVKDQSLKLAGGSSYATSKLDKL 1003
AAQ05800.1_Hex20A_C. fimi          ---------------------------------------------------------------------- 353
BAI63641.1_Hex1t_Paenibacillus     ---------------------------------------------------------------------- 377
CAA55582.1_NahA_P. gingivalis      ---------------------------------------------------------------------- 407
BAA84281.1_Hex99_P. piscicida      ---------------------------------------------------------------------- 678
BAB84321.1_Hex86_P. piscicida      ---------------------------------------------------------------------- 411
AAB03808.1_Chb_S. marcescens       ---------------------------------------------------------------------- 646
AAS02154.1_GcnA_S. gordonii        ---------------------------------------------------------------------- 400
AAK98861.1_StrH_S. pneumoniae      ---------------------------------------------------------------------- 880
CAB87223.1_HexA_S. coelicolor      ---------------------------------------------------------------------- 399
AAC38798.3_SpHex_S. plicatus       ---------------------------------------------------------------------- 370
AKC34128.1_Hex1_uncultured Act     ---------------------------------------------------------------------- 359
AKC34129.1_Hex2_uncultured Bac     ---------------------------------------------------------------------- 400
AAC44672.1_ExoI_V. furnissii       ---------------------------------------------------------------------- 504
AAA88682.1_Chb_V. harveyi          ---------------------------------------------------------------------- 645
AAD30612.1_AtHex1_A. thaliana      ---------------------------------------------------------------------- 416
AAM91092.1_AtHex3_A. thaliana      ---------------------------------------------------------------------- 401
BAE99290.1_AtHex3_A. thaliana      ---------------------------------------------------------------------- 404
AAM13977.1_HexA_A. oryzae          ---------------------------------------------------------------------- 425
AAT99455.1_BmGlcNAcase3_B. mor     ---------------------------------------------------------------------- 408
AAC60521.1_BmChiGlcNAcase_B. m     ---------------------------------------------------------------------- 456
CCD73477.2_CeHEX-4_C. elegans      ---------------------------------------------------------------------- 351
AAA34346.2_Hex1_C. albicans        ---------------------------------------------------------------------- 393
ADU20405.1_βHex_C. annuum          ---------------------------------------------------------------------- 409
ABB18373.1_CpHEX1_C. posadasii     ---------------------------------------------------------------------- 420
AAA33230.1_NagA_D. discoideum      ---------------------------------------------------------------------- 379
AAF46406.1_Hex2_D. melanogaste     ---------------------------------------------------------------------- 493
AAF47881.1_Hex1_D. melanogaste     ---------------------------------------------------------------------- 459
AAM29423.1_Fdl_D. melanogaster     ---------------------------------------------------------------------- 512
CAE46968.1_HexA_E. histolytica     ---------------------------------------------------------------------- 417
AAB00965.1_HexA_H. sapiens         ---------------------------------------------------------------------- 400
AAA52645.1_HexB_H. sapiens         ---------------------------------------------------------------------- 429
AAC53246.1_HexA_M. musculus        ---------------------------------------------------------------------- 399
AAA18776.1_HexB_M. musculus        ---------------------------------------------------------------------- 408
ABI81756.1_Hex1_O. furnacalis      ---------------------------------------------------------------------- 454
AEQ33603.1_HexTF_T. flavus         ---------------------------------------------------------------------- 450
ACI15900.1_NagA_T. maritima        ---------------------------------------------------------------------- 337
AAK16587.1_CbsA_T. neapolitana     ---------------------------------------------------------------------- 337
CAA63123.1_HexB_S. scrofa          ---------------------------------------------------------------------- 403
```

```
                                      1130      1140      1150      1160      1170      1180      1190
                               ....|....|....|....|....|....|....|....|....|....|....|....|....|....|
WP_082768773.1_Nah20B_P. hydro -----------------------------FDRAHDFVNRGWDTVLSFPDVLYFDFPYQAD---------- 660
WP_068373836.1_Nah20A_P. hydro -----------------------QHWQFTMPRLKGSAVSGEFVLLSSEAGKQVLLRFNNQQARLINELEP 485
BAD00143.1_Nah20A_A. hydrophil -----------------------------FNEAMKWAHKGYDVVLSNPDYLYFDFPNEVH---------- 680
BAF76001.1_NahB_A. hydrophila -----------------------------FQAGLDAAASGYPVVMAPAQHLYLDLAWSQD---------- 517
BAI94822.1_BbhI_B. bifidum    GNGNELTFDVTLQQAAKPGDILFEADAPYGTHDIRVMENGKLGFTRELYNYYFDYELPVGKTVTVTIKVD 1073
AAQ05800.1_Hex20A_C. fimi     --------------------------------FVAAAQAGAHVLMSPGSRAYLDMKYDA----------- 380
BAI63641.1_Hex1t_Paenibacillus ---------------------------------TAAKAKGMKVIVSPAN-AYLDMKYYS----------- 402
CAA55582.1_NahA_P. gingivalis ---------------------------------IAAANMNHDVIMTPGSGGLYLDHYQG----------- 433
BAA84281.1_Hex99_P. piscicida -------------------------------QAYKFANQGHKVVLAHATNLYLDLAYNKHP--------- 708
BAB84321.1_Hex86_P. piscicida ---------------------------------IQAAKMGHQVIMSPYQ-YIYFDAYQS----------- 436
AAB03808.1_Chb_S. marcescens  -----------------------------FDSVNDWANKGYEVVVSNPDYVYMDFPYEVN---------- 677
AAS02154.1_GcnA_S. gordonii   --------------------------------DLKKVSEHFLVSTGLDFDDFMKIDLANLLP-------- 430
AAK98861.1_StrH_S. pneumoniae -----------------------------LASPQYLASKGYKFLNTNGDWYYVIGNHKQDEAYPLSKAVE 921
CAB87223.1_HexA_S. coelicolor -----------------------------KAQVAAAARNGTGLILSPADRTYLDMKYTK----------- 429
AAC38798.3_SpHex_S. plicatus  -----------------------------KAEVAEAARNGTGLILSPADRTYLDMKYTK----------- 400
AKC34128.1_Hex1_uncultured Act ---------------------------------PQAAASGHKVILSPAGKTYLDMKYDE----------- 385
AKC34129.1_Hex2_uncultured Bac ---------------------------------KHALSLGNNVVMATTS-HTYLDYYQNPAATEL----- 431
AAC44672.1_ExoI_V. furnissii  -----------------------------EQAALNCARQGFDVILQPGQFTYLDIAQDYA---------- 535
AAA88682.1_Chb_V. harveyi     -----------------------------TSSVYEWSKKGYDVIVSNPDYVYMDMPYEVD---------- 676
AAD30612.1_AtHex1_A. thaliana -------------------------------TKR-IVAAGYRVIVSSSEFYYLDCGHGGFLGNDSIYDQ- 453
AAM91092.1_AtHex3_A. thaliana -------------------------------LVENVTASGLRCIVSNQEFWYLDHID------------- 427
BAE99290.1_AtHex3_A. thaliana -------------------------------ICQKAVAKGFRCIFSNQGYWYLDHLD------------- 430
AAM13977.1_HexA_A. oryzae     -------------------------------ISN-LTARGYDVIVSSSDFLYLDCGHGGFVTNDPRYNVM 463
AAT99455.1_BmGlcNAcase3_B. mor -----------------------------APEMLNILRASHQLIYSTG--WYLDHLN------------- 434
AAC60521.1_BmChiGlcNAcase_B. m -------------------------------QIQGLLQKGYRLIMSNYDALYFDCGFGAWVGS------- 488
CCD73477.2_CeHEX-4_C. elegans  -------------------------------ALCELLPVAIPSLIVDLFYLNYQLTEK------------ 378
AAA34346.2_Hex1_C. albicans   -------------------------------VKN-LTSRGYDVVVSSSDFLYLDCGNAGWVTNDPRY--V 429
ADU20405.1_βHex_C. annuum     -------------------------------TKQ-LVTSGYRVIVSSADYYYLDCGHGSFVGNDSRYDQP 447
ABB18373.1_CpHEX1_C. posadasii -------------------------------IKK-LTARGYEVIVSSADFLYLDCGYGGWVGNDPRYNVM 458
AAA33230.1_NagA_D. discoideum -------------------------------DLQGIVNSGYKALVSFA--WYLDKQNPDN---------- 406
AAF46406.1_Hex2_D. melanogaste -------------------------------LNRELLQRGYRLIVSTKNAWYLDHGFWGSTS-------- 524
AAF47881.1_Hex1_D. melanogaste -------------------------------KVKKILERGYKIIVSNYDALYLDCGGAGWVTD------- 491
AAM29423.1_Fdl_D. melanogaster -------------------------------ENYDLLDNGYNVIFSHVDAWYLDCGFGSWRAT------- 544
CAE46968.1_HexA_E. histolytica -------------------------------LLQQVVNSGYKAIFSAG--FYLDKQMPLCNSYDSST--- 451
AAB00965.1_HexA_H. sapiens    -----------------------------MKELELVTKAGFRALLSAP--WYLNRIS------------- 426
AAA52645.1_HexB_H. sapiens    -----------------------------PEELSRVTASGFPVILSAP--WYLDLIS------------- 455
AAC53246.1_HexA_M. musculus   -----------------------------MLEMQDITRAGFRALLSAP--WYLNRVK------------- 425
AAA18776.1_HexB_M. musculus   -----------------------------SYELKQVTGSGFPAILSAP--WYLDLIS------------- 434
ABI81756.1_Hex1_O. furnacalis -------------------------------QIKGLLEKGYRLIMSNYDALYFDCGYGAWVGA------- 486
AEQ33603.1_HexTF_T. flavus    -------------------------------INN-LTNLGYDVLVSSSDFMYLDCGYGGFVTNDPRYDVM 488
ACI15900.1_NagA_T. maritima   ---------------------------------FEKIDHTSEVTLLVPSSENLSQADTT----------- 363
AAK16587.1_CbsA_T. neapolitana ---------------------------------MRKELLGREVVLLVPSNKNLSPADTT----------- 363
CAA63123.1_HexB_S. scrofa     -----------------------------KWEQSLITKAGFPVILSAP--WYLDLIS------------- 429
```

```
                                     1200      1210      1220      1230      1240      1250      1260
                                ....|....|....|....|....|....|....|....|....|....|....|....|....|
WP_082768773.1_Nah20B_P. hydro  -------------------------PKEPGYYWGMRSIDSFKVFQFMPDNLPAHAEFWTDAIGNDYQSTE 705
WP_068373836.1_Nah20A_P. hydro  RANLQRFTV-----DSWMGPSQFELDLSKDVIQQGRILIGNTPYSVSGNRLAQSTGSKALPAMLQDFVAA 550
BAD00143.1_Nah20A_A. hydrophil  -------------------------PAERGYYWATRFNDTRKVFAFAPENLPQNAETSVDRDGNAFVAKG 725
BAF76001.1_NahB_A. hydrophila   -------------------------IHEPGLYWAG-TLNLAQVHACDP---APADFHANDN--------- 549
BAI94822.1_BbhI_B. bifidum     QQTTKLYVDGEFVSDATGKYIDKGIEKKTGITAATFALPLQRIGSKTSAINGVIDNVIVKKSEAETDQYN 1143
AAQ05800.1_Hex20A_C. fimi       -------------------------TTELGLEWAG-HIELRDAYDWEP-STLIPGVP------------- 410
BAI63641.1_Hex1t_Paenibacillus  -------------------------DSPIGLQWRG-FVNTNRAYNWDP-TDCIKGA-------------- 431
CAA55582.1_NahA_P. gingivalis   ------------------------DPTVEPVAIGG-YAPLEQVYAYNPLPKELPADK------------- 465
BAA84281.1_Hex99_P. piscicida   --------------------------DEIGYHWAG-YTDTKKAFEYRPFNIYANAERDKLGNPVPWDPKW 751
BAB84321.1_Hex86_P. piscicida   ------------------------RNLDEPKAIHG-LSSLKNVYQYEPQPSHLTAEQ------------- 468
AAB03808.1_Chb_S. marcescens    -------------------------PDERGYYWGTRFSDERKVFSFAPDNMPQNAETSVDRDGNHFNAKS 722
AAS02154.1_GcnA_S. gordonii     ---------------------------DLPDNLSGINPNRYVLYQDVLCPLLEQHIRPEKD--------- 464
AAK98861.1_StrH_S. pneumoniae   --------------------------NSGKVPFNQLASTKYPEVDLPTVGSMLSIWADRPSAEYKEEEIF 965
CAB87223.1_HexA_S. coelicolor   -------------------------DTPLGLSWAG-YVEVRRSYDWDP-AAYLPGAP------------- 459
AAC38798.3_SpHex_S. plicatus    -------------------------DTPLGLSWAG-YVEVQRSYDWDP-AGYLPGAP------------- 430
AKC34128.1_Hex1_uncultured Act  -------------------------ATPFGLSWAG-YVTVQDAFAWNP-GAYLAGVG------------- 415
AKC34129.1_Hex2_uncultured Bac  ------------------------AKGVEYEAIGG-HLPLEKVYSYNPTFVAENPQQ------------- 463
AAC44672.1_ExoI_V. furnissii    -------------------------PEEPGVDWAG-VTPLERAYRYEPLVEVPEHDPLRKR--------- 570
AAA88682.1_Chb_V. harveyi       -------------------------PKERGYYWATRATDTRKMFGFAPENMPQNAETSVDRDGNGFTGKG 721
AAD30612.1_AtHex1_A. thaliana   ---------------KESG----------GGSWCAPFKTWQSIYNYD-IADGLLNEEERKL--------- 488
AAM91092.1_AtHex3_A. thaliana   ------------------------------APWQGFYANEPFQNITD--------KKQQSL--------- 450
BAE99290.1_AtHex3_A. thaliana   ------------------------------VPWEEVYNTEPLNGIED--------PSLQKL--------- 453
AAM13977.1_HexA_A. oryzae       AN-----------PDANT--PNFNYYGGNCGSWCAPYKTWQRIYDYD-FTLN-LTETQAKH--------- 508
AAT99455.1_BmGlcNAcase3_B. mor  ----------------------------TGGDWTEFFNKDPRDLVN-----GLSKDINVDN--------- 462
AAC60521.1_BmChiGlcNAcase_B. m  -----------------------------GNNWCSPYIGGQKVYGN---SPAVMALSYRDQ--------- 517
CCD73477.2_CeHEX-4_C. elegans   ------------------------------DAWRAMKSSLECENRRHLRG-------------------- 398
AAA34346.2_Hex1_C. albicans     ET------------PENVD----FNTG-QCGSWCGPYKSYQRIYNFD-FTAN-LTETEKNH--------- 471
ADU20405.1_βHex_C. annuum       PG------------TDQGN----------GGSWCGPFKTWETIYNYD-ITYG-LTDKEAQL--------- 484
ABB18373.1_CpHEX1_C. posadasii  EN-----------PDPET--PNFNYYGGNCGSWCGPYKTWQRIYNYD-FTDG-LNYAEKKR--------- 503
AAA33230.1_NagA_D. discoideum   ------------------------------NIHYEWQDTWQDFYAAD--PTNNISTN-AEN--------- 434
AAF46406.1_Hex2_D. melanogaste  ------------------------------------YYNWRTVYSS-----GMPVGRSKDQ--------- 544
AAF47881.1_Hex1_D. melanogaste  -----------------------------GNNWCSPYIGWQKVYDN---SLKSIAGDYEHH--------- 520
AAM29423.1_Fdl_D. melanogaster  -----------------------------GDAACAQYRTWQNVYKHRPWERMRLDKKRKKQ--------- 576
CAE46968.1_HexA_E. histolytica  ----------------------------CVNTHSMWVWTNRDMYDND--PVKSLSSSEKEN--------- 482
AAB00965.1_HexA_H. sapiens      ----------------------------YGPDWKDFYVVEPLAFE------GTPEQKAL----------- 451
AAA52645.1_HexB_H. sapiens      ----------------------------YGQDWRKYYKVEPLDFG------GTQKQKQL----------- 480
AAC53246.1_HexA_M. musculus     ----------------------------YGPDWKDMYKVEPLAFH------GTPEQKAL----------- 450
AAA18776.1_HexB_M. musculus     ----------------------------YGQDWKNYYKVEPLNFE------GSEKQKQL----------- 459
ABI81756.1_Hex1_O. furnacalis   -----------------------------GNNWCSPYIGWQKVYDN---SPAVIALEHRDQ--------- 515
AEQ33603.1_HexTF_T. flavus      VN------------PDAVDGLANFNWGGNCGSWCAPYKTWQRIYDYD-FTTN-LTDAQAAH--------- 535
ACI15900.1_NagA_T. maritima     -----------------------------GGDYDQIPEIVSRFFEVENVVRYTVEDGPE----------- 393
AAK16587.1_CbsA_T. neapolitana  -----------------------------GDDYDLIPEVAKRFFKVRDVIRYDIEAGPD----------- 393
CAA63123.1_HexB_S. scrofa       ----------------------------YGQDWKNYYEVEPQDFP------GSDKERKR----------- 454
```

```
                                                  1270      1280      1290      1300      1310      1320      1330
                                          ....|....|....|....|....|....|....|....|....|....|....|....|....|....|
WP_082768773.1_Nah20B_P. hydro            KVS----LQNQAKVSGIQAQLWSETVRSDDSANYMLFP---RLIAFAERAWFKPEWAVAYQAGQT----- 763
WP_068373836.1_Nah20A_P. hydro            SKVPTDLPKLHKNILGGEAAIWSEIV-RADNLDLRLWP---RLYAIAERLWSPATLTDEDAMYRR----- 611
BAD00143.1_Nah20A_A. hydrophil            DQ-------DPVKFKGISGQQWSETVRTDAQYEYMVYP---RIFSVAERAWHKGGFELDYVKGRE----- 780
BAF76001.1_NahB_A. hydrophila             -------------ILGVLSPLWSELITSRDRLDYMLFP---RMLATAEVAWSTPARKVWEH--------- 594
BAI94822.1_BbhI_B. bifidum               KSCWTGTTNSETQYNDTEGLLRYAFDNNPSTIWHSNWKGATDKLTGSNSFYAEIDMCQKYTINQFSF--- 1210
AAQ05800.1_Hex20A_C. fimi                ----------PESVIGVEAAVWTETLTDLGELTSMLLP---RLAAVAEVAWTAPQDRDWDDFSG------ 461
BAI63641.1_Hex1t_Paenibacillus           ------------NIYGVESTLWTETFVTQDHLDYMLYP---KLLSNAEVGWTARGDRNWDDFKE------ 480
CAA55582.1_NahA_P. gingivalis            ----------HRYVLGAQANLWAEYLYTSERYDYQAYP---RLLAVAELTWTPLAKKDFADFCR------ 516
BAA84281.1_Hex99_P. piscicida            VLLSEAG---KQNVQGIQAQLFGENQKSPEILDYMTFP---KLLGAAERAWVQDMPAQGAATTEA----- 810
BAB84321.1_Hex86_P. piscicida            ----------QAFIVGAQGALWTEYIKTPRHAEYMLFP---RLSALAETLWSDKTQKSWSDYSQY----- 520
AAB03808.1_Chb_S. marcescens             DK-------PWPGAYGLSAQLWSETQRTDPQMEYMIFP---RALSVAERSWHRAGWEQDYRAGRE----- 777
AAS02154.1_GcnA_S. gordonii              -----------KQHFASSAQQLGEISKRAGEYAYIFET-QAQLNALLALKISITSGIQKAYRNGD----- 517
AAK98861.1_StrH_S. pneumoniae            ELMTAFADHNKDYFRANYNALREELAKIPTNLEGYSKESLEALDAAKTALNYNLNRNKQAELDTLVANLK 1035
CAB87223.1_HexA_S. coelicolor            ----------AEAVRGVEAPLWTETLSDPDQLDFMAFP---RLPGVAELGWSPASTHDWDTYKV------ 510
AAC38798.3_SpHex_S. plicatus             ----------ADAVRGVEAPLWTETLSDPDQLDYMAFP---RLPGVAELGWSPASTHDWDTYKV------ 481
AKC34128.1_Hex1_uncultured Act           ----------EDAVAGVEAPLWTETIPTFAEAEFMLFP---RLLAVAELGWSPAAGRTWDEFRS------ 466
AKC34129.1_Hex2_uncultured Bac           ----------EKQILGTQAQLWSEYFKDMKKVEYHAFP---RIAALAEVAWTPLALKNFDGFSK------ 514
AAC44672.1_ExoI_V. furnissii             -------------ILGIQCALWCELVNNQDRMDYMIYP---RLTALAGSGLDT----------------- 607
AAA88682.1_Chb_V. harveyi                EI-------EAKPFYGLSAQLWSETVRNDEQYEYMVFP---RVLAAAQRAWHRADWENDYKVGVE----- 776
AAD30612.1_AtHex1_A. thaliana            -------------VLGGEVALWSEQA-DSTVLDSRLWP---RASALAESLWSGNRD-ERGVKRCGEA--- 537
AAM91092.1_AtHex3_A. thaliana            -------------VLGGEVCMWGEHI-DASDIEQTIWP---RAAAAAERLWTPYAKLAKNPNNVTT---- 499
BAE99290.1_AtHex3_A. thaliana            -------------VIGGEVCMWGETA-DTSVVLQTIWP---RAAAAAERMWSTREAVSKGNITLTAL--- 503
AAM13977.1_HexA_A. oryzae                -------------IIGATAPLWGEQV-DDINVSSMFWP---RAAALAELVWSGNRD-ANGNKRTTEM--- 557
AAT99455.1_BmGlcNAcase3_B. mor           -------------IVGGEACMWAEVV-NDMNIMSRVWP---RASAVAERLWG-HESQATYQVHC------ 508
AAC60521.1_BmChiGlcNAcase_B. m           -------------ILGGEVALWSEQS-DPATLDGRLWP---RAAAFAERMWAEPSTAWQDAEH------- 563
CCD73477.2_CeHEX-4_C. elegans            --------------ILAESTVHGCKFPGADVFEIIMHD---WKRAVDRRIFGKPDQQPSPDSEAIEILKN 451
AAA34346.2_Hex1_C. albicans              -------------VLGREAALWSEQV-DSTVLTTKIWP---RTAALAELTWSGNKD-SNGHHRGYEF--- 520
ADU20405.1_βHex_C. annuum                -------------VIGGEVALWSEQA-DSTVMDSRIWP---RASAMAETLWSGNCD-ETGMKRYAEA--- 533
ABB18373.1_CpHEX1_C. posadasii           -------------VIGAIAPLWSEQA-DDVVISNKMWP---RAAALAELVWSGNVG-KDGKKRTTLM--- 552
AAA33230.1_NagA_D. discoideum            -------------IIGGEATMWAEQI-NQVNWDVRVWP---RAIGIAERLWSAQSVNSVSLALP------ 481
AAF46406.1_Hex2_D. melanogaste           -------------VLGGEVCMWSEYV-DQNSLESRIWP---RAGAAAERMWSNPKSSALLAQR------- 590
AAF47881.1_Hex1_D. melanogaste           -------------VLGAEGAIWSEQI-DEHTLDNRFWP---RASALAERLWSNPAEGWRQAES------- 566
AAM29423.1_Fdl_D. melanogaster           -------------VLGGEVCMWTEQV-DENQLDNRLWP---RTAALAERLWTDPSDDHDMDIVPPDV--- 626
CAE46968.1_HexA_E. histolytica           -------------VLGGEGCSWGEST-DEQNFFDRVFQ---RYSAIAERLWSKESVVDKESHEV------ 529
AAB00965.1_HexA_H. sapiens               -------------VIGGEACMWGEYV-DNTNLVPRLWP---RAGAVAERLWSNKLTSDLTFAYE------ 498
AAA52645.1_HexB_H. sapiens               -------------FIGGEACLWGEYV-DATNLTPRLWP---RASAVGERLWSSKDVRDMDDAYD------ 527
AAC53246.1_HexA_M. musculus              -------------VIGGEACMWGEYV-DSTNLVPRLWP---RAGAVAERLWSSNLTTNIDFAFK------ 497
AAA18776.1_HexB_M. musculus              -------------VIGGEACLWGEFV-DATNLTPRLWP---RASAVGERLWSPKTVTDLENAYK------ 506
ABI81756.1_Hex1_O. furnacalis            -------------VLGGEAALWSEQS-DTSTLDGRLWP---RAAALAERLWAEPATSWQDAEY------- 561
AEQ33603.1_HexTF_T. flavus               -------------VKGAVAPLWSEQV-DDTVISGKMWP---RAAALAELVWSGNKDPKTGDKRTTYM--- 585
ACI15900.1_NagA_T. maritima              ---------------FVEGDLIFDFVADIP-NEKALKA---HLSLPAEKTVYFVLRNPFDVRYFE----- 439
AAK16587.1_CbsA_T. neapolitana           ---------------DVDGELIFDFVVNASKNEQVLQA---HLSLPSDRTIYFIIRNPFDAKFFP----- 440
CAA63123.1_HexB_S. scrofa                -------------VLGGEACLWGEYV-DATNLTPRLWP---RASAVGERLWSHKDVRDIHDAYS------ 501
```

```
                                   1340      1350      1360      1370      1380      1390      1400
                               ....|....|....|....|....|....|....|....|....|....|....|....|....|....|
WP_082768773.1_Nah20B_P. hydro --------YSRDSKLFTELKQQQMYQDWAQFSQTMGSQILTDLARDEVLFRLPTPGAKIKQGWLHANSMFS 826
WP_068373836.1_Nah20A_P. hydro -----LTVMQTYASEVIGLEFTQQQRAGFKQLTSQDADIEPLIILAEAVEQAQYYTRHHSKFQHDNYHQL 676
BAD00143.1_Nah20A_A. hydrophil -------FSG-TTKHVNK--ATLNKEWNQFANVLGQRVLPKLDQAGVEYRLSVPGAKVVNGVLEANVDLP 840
BAF76001.1_NahB_A. hydrophila  ------------------------------------------------FVARLPGQLHILDQLQVRYRVP 616
BAI94822.1_BbhI_B. bifidum     -----TPRTSQDSGQVTKADLYVKANANDEWKQVATDQVFEASRAKKTFMFDEQEVRYVKFVAKSSNDGW 1275
AAQ05800.1_Hex20A_C. fimi      -------RVAQHAPFWDRVGFRWHASPQVSWPGPGSAPGAAF---------------------------- 496
BAI63641.1_Hex1t_Paenibacillus -------RLIEHTPRLQNKGIKFFADP-IVWELPIVQINSEWKMDEGTGTVVKDTSGYLNGTLVGGAKWT 542
CAA55582.1_NahA_P. gingivalis  -------RLDNACVRLDMHGINYHIPLPEQPGGSSDFIAFTDKAKLTFTTSRPMKMVYTLDETEPSLTST 579
BAA84281.1_Hex99_P. piscicida  -------------WHTFTNTLGQYALPLLDYYQVVDINAQIPQQVGVNYRIPLPGGAIEGGKLIVNTRFV 867
BAB84321.1_Hex86_P. piscicida  -------RLPALLKRYQKMHLNTAYSSHKP--IISSEINGQQLIATITSEIADTVIYYTLDGSEPTLQSQ 581
AAB03808.1_Chb_S. marcescens   -------YKGGETHFVDT--QALEKDWLRFANILGQRELAKLDKGGVAYRLPVPGARVAAGKLEANIALP 838
AAS02154.1_GcnA_S. gordonii    ---------KEHLSALAEKDFPQLYQMVEDFSDQFSRQWQQENKIFGLDTIDIRFGGLLKRIKRAQERLE 578
AAK98861.1_StrH_S. pneumoniae  AALQGLKPAATHSGSLDENEVAANVETRPELITRTEEIPFEVIKKENPNLPAGQENIITAGVKGERTHYI 1105
CAB87223.1_HexA_S. coelicolor  -------RLAGQAPHWEAMGIDYYRSPQVPWT-------------------------------------- 535
AAC38798.3_SpHex_S. plicatus   -------RLAAQAPYWEAAGIDFYRSPQVPWT-------------------------------------- 506
AKC34128.1_Hex1_uncultured Act -------RLAHHGPRLEGLGVNFFRAPEIDWPETL----------------------------------- 494
AKC34129.1_Hex2_uncultured Bac -------RLEGIMQHYEAGNLHYCKPSTEK---------------------------------------- 537
AAC44672.1_ExoI_V. furnissii   ---------------------------------------------------KIPA--------------- 611
AAA88682.1_Chb_V. harveyi      -------YSQ-NSNLVDK--ASLNQDYNRFANVLGQRELAKLEKSGIDYRLPVPGAKVEDGKLAMNVQFP 836
AAD30612.1_AtHex1_A. thaliana  -----VDRLNLWRYRMVKRGIGAE--PIQPLWCLKNPGMCNTVHGALQDQ-------------------- 580
AAM91092.1_AtHex3_A. thaliana  -------RLAHFRCLLNQRGVAAA--PLVGG-GRVVPFEPGSCLAQ------------------------ 535
BAE99290.1_AtHex3_A. thaliana  ------PRLHYFRCLLNNRGVPAA--PVDNFYARRPPLGPGSCYAQ------------------------ 541
AAM13977.1_HexA_A. oryzae      -----TQRILNFREYLVANGVQAQ--ALVPKYCLQHPHACDLYRNQAAIQ-------------------- 600
AAT99455.1_BmGlcNAcase3_B. mor -------RLEEHTCRMNARGIHAQ--PPSGPGFCLGV--------------------------------- 536
AAC60521.1_BmChiGlcNAcase_B. m -------RMLHVRERLVRMGIQAE--SLEPEWCYQNQGLCYG---------------------------- 596
CCD73477.2_CeHEX-4_C. elegans  ------LKKSLNQSILYKTDADEVFNQYLHDYHSLIAQNTRTEITN------------------------ 491
AAA34346.2_Hex1_C. albicans    -----TQRILNFREYLVKLGYGVS--PLVPKYCLLNPHACDLYKNPPVY--------------------- 562
ADU20405.1_βHex_C. annuum      -----TDRLTEWRYRMVARGIGAE--PIQPLWCVKNSGMCNTVHSFTS---------------------- 574
ABB18373.1_CpHEX1_C. posadasii -----TQRILNFREYLVANGIMAA--PLQPKYCLKHPHSCDLYYDQTVIM-------------------- 595
AAA33230.1_NagA_D. discoideum  -------RIGHFTCDLSRRGIQS--GPLFPDYCPMQDDLVFTMKPNTKLSKSEIKLILNK---------- 532
AAF46406.1_Hex2_D. melanogaste -------RFYRYRERLLARGIHAD--AVIPHWCVLHEGQCL----------------------------- 622
AAF47881.1_Hex1_D. melanogaste -------RLLLHRQRLVDNGLGAE--AMQPQWCLQNEHECPIDACSRGSGRLGLIVLLLLTTLSA----- 622
AAM29423.1_Fdl_D. melanogaster -----FRRISLFRNRLVELGIRAE--ALFPKYCAQNPGECI----------------------------- 660
CAE46968.1_HexA_E. histolytica -------RANYLRCLDVRRDIMKGTGPLYHSFCQLPKKEKSN---------------------------- 564
AAB00965.1_HexA_H. sapiens     -------RLSHFRCELLRRGVQAQ--PLN-VGFCEQEFEQT----------------------------- 529
AAA52645.1_HexB_H. sapiens     -------RLTRHRCRMVERGIAAQ--PLY-AGYCNHENM------------------------------- 556
AAC53246.1_HexA_M. musculus    -------RLSHFRCELVRRGIQAQ--PIS-VGYCEQEFEQT----------------------------- 528
AAA18776.1_HexB_M. musculus    -------RLAVHRCRMVSRGIAAQ--PLY-TGYCNYENKI------------------------------ 536
ABI81756.1_Hex1_O. furnacalis  -------RMLHIRERLVRMGIQAE--SLQPEWCYQNEGYCYS---------------------------- 594
AEQ33603.1_HexTF_T. flavus     -----TQRILNFREYLVANGVQAA--PLVPKYCLQHPHSCDLYYDQTAVV-------------------- 628
ACI15900.1_NagA_T. maritima    -----------GRKIVVTRSTKPISIYKSLEHFLGRCDS------------------------------- 467
AAK16587.1_CbsA_T. neapolitana -----------GRSVVITHSTKPISVYKSFQHLLGRCS-------------------------------- 467
CAA63123.1_HexB_S. scrofa      -------RLTIHRCRMVRRGIAAE--PLF-TGYCNHEHRM------------------------------ 531
```

```
                                           1410      1420      1430      1440      1450      1460      1470
                                  ....|....|....|....|....|....|....|....|....|....|....|....|....|....|
WP_082768773.1_Nah20B_P. hydro    GLTIEYQQEGEKQWQVYSQPVK--VTKPVLLRSSMPGLLRYSRSVSVSMQQKVEE--------------- 879
WP_068373836.1_Nah20A_P. hydro    VELNQYVDYLSAQSLGLVELKRHIELFQQGDQQAKKFIEQLLQKWQKNLPKLKLLIANYPKLHSLAPLVT 746
BAD00143.1_Nah20A_A. hydrophil    GLPIQYSLDG-KSWSAYDAAAKPTVDGKVYLRTTSFDGKRTSRVTELN---------------------- 887
BAF76001.1_NahB_A. hydrophila     QR-------------------------------------------------------------------- 618
BAI94822.1_BbhI_B. bifidum        VAVSEFGVANKPSSTVRVFVAADPAEGGTVSVAAEGETGTDTAVDVASGASVTAKAVAADGYRFSGWFTT 1345
AAQ05800.1_Hex20A_C. fimi         ---------------------------------------------------------------------- 496
BAI63641.1_Hex1t_Paenibacillus    AGKQGNGVSFDGSSGYINLGGQDITGNWTAAVWVYGQPNTTNNETLLSGTTSAIKINQYNKTGKVGITIY 612
CAA55582.1_NahA_P. gingivalis     PYTVPLEFAQTGLLKIR------------TVTAGG--KMSPVRRIRVEKQPFNMSMEVPAPKPGLTIRTA 635
BAA84281.1_Hex99_P. piscicida     GMATEYSVDQGVTWHRYEGPVTLTASSKVQLRTVSDSGKVSRVATVN----------------------- 914
BAB84321.1_Hex86_P. piscicida     QYQKPLVITDETAXRAR------------SYVSDLGQLVGDARLTLSPHLALGKEITLASLAAEGSATKL 639
AAB03808.1_Chb_S. marcescens      GLGIEYSTDGGKQWQRYDAKAKPAVSGEVQVRSVSPDGKRYSRAEKV----------------------- 885
AAS02154.1_GcnA_S. gordonii       QFISGQIDCVEELEQEILPFNDFYKDQGLTATTANQWHLIATASTIYTT--------------------- 627
AAK98861.1_StrH_S. pneumoniae     SVLTENGKTTETVLDSQVTKEVINQVVEVGSPVTHKGDESGLAPTTEVKPRLDIQEEEIPFTTVTRENPL 1175
CAB87223.1_HexA_S. coelicolor     ---------------------------------------------------------------------- 535
AAC38798.3_SpHex_S. plicatus      ---------------------------------------------------------------------- 506
AKC34128.1_Hex1_uncultured Act    ---------------------------------------------------------------------- 494
AKC34129.1_Hex2_uncultured Bac    ---------------------------------------------------------------------- 537
AAC44672.1_ExoI_V. furnissii      ---------------------------------------------------------------------- 611
AAA88682.1_Chb_V. harveyi         GVTLQYSLDG-ENWLTYADNARPNVTGEVFIRSVSATGEKVSRITSVK---------------------- 883
AAD30612.1_AtHex1_A. thaliana     ---------------------------------------------------------------------- 580
AAM91092.1_AtHex3_A. thaliana     ---------------------------------------------------------------------- 535
BAE99290.1_AtHex3_A. thaliana     ---------------------------------------------------------------------- 541
AAM13977.1_HexA_A. oryzae         ---------------------------------------------------------------------- 600
AAT99455.1_BmGlcNAcase3_B. mor    ---------------------------------------------------------------------- 536
AAC60521.1_BmChiGlcNAcase_B. m    ---------------------------------------------------------------------- 596
CCD73477.2_CeHEX-4_C. elegans     ---------------------------------------------------------------------- 491
AAA34346.2_Hex1_C. albicans       ---------------------------------------------------------------------- 562
ADU20405.1_β Hex_C. annuum        ---------------------------------------------------------------------- 574
ABB18373.1_CpHEX1_C. posadasii    ---------------------------------------------------------------------- 595
AAA33230.1_NagA_D. discoideum     ---------------------------------------------------------------------- 532
AAF46406.1_Hex2_D. melanogaste    ---------------------------------------------------------------------- 622
AAF47881.1_Hex1_D. melanogaste    ---------------------------------------------------------------------- 622
AAM29423.1_Fdl_D. melanogaster    ---------------------------------------------------------------------- 660
CAE46968.1_HexA_E. histolytica    ---------------------------------------------------------------------- 564
AAB00965.1_HexA_H. sapiens        ---------------------------------------------------------------------- 529
AAA52645.1_HexB_H. sapiens        ---------------------------------------------------------------------- 556
AAC53246.1_HexA_M. musculus       ---------------------------------------------------------------------- 528
AAA18776.1_HexB_M. musculus       ---------------------------------------------------------------------- 536
ABI81756.1_Hex1_O. furnacalis     ---------------------------------------------------------------------- 594
AEQ33603.1_HexTF_T. flavus        ---------------------------------------------------------------------- 628
ACI15900.1_NagA_T. maritima       ---------------------------------------------------------------------- 467
AAK16587.1_CbsA_T. neapolitana    ---------------------------------------------------------------------- 467
CAA63123.1_HexB_S. scrofa         ---------------------------------------------------------------------- 531
```

```
                                       1480      1490      1500      1510      1520      1530      1540
                               ....|....|....|....|....|....|....|....|....|....|....|....|....|....|
WP_082768773.1_Nah20B_P. hydro ---------------------------------------------------------------------- 879
WP_068373836.1_Nah20A_P. hydro TLSQVVDIGQKLVAQCAKGEPLKPEHQRDVQQQLWQAAQLQQEMVVSSALAIEALAFKCRA--------- 807
BAD00143.1_Nah20A_A. hydrophil ---------------------------------------------------------------------- 887
BAF76001.1_NahB_A. hydrophila  ---------------------------------------------------------------------- 618
BAI94822.1_BbhI_B. bifidum     ASETAVSTDATYTFAADGNTALTAKFTKDSTPDPGPKPTISSIAVTKPTVTDYKVGDTFDATGLAVTATM 1415
AAQ05800.1_Hex20A_C. fimi      ---------------------------------------------------------------------- 496
BAI63641.1_Hex1t_Paenibacillus GTKDYTYNYSIPSNKWTHLTFVGTSTGTALYENGVLKETIAAKMNGPMALVGAEKTGGSGDLTSYFRGSL 682
CAA55582.1_NahA_P. gingivalis  YGDLYDVPDLQQVASWEVGTVSSLEEIMHGKEKITSPEVLERRVVEATGYVLIPEDGVYEFSTENNEFWI 705
BAA84281.1_Hex99_P. piscicida  ---------------------------------------------------------------------- 914
BAB84321.1_Hex86_P. piscicida  QDGQFAYDQFYSVDDYAIFYDTDLEAVIDLDASTQVQQVKLG-FDSGRHRQLHPPTHIQVLGSSDKQQWQ 708
AAB03808.1_Chb_S. marcescens   ---------------------------------------------------------------------- 885
AAS02154.1_GcnA_S. gordonii    ---------------------------------------------------------------------- 627
AAK98861.1_StrH_S. pneumoniae  LLKGKTQVITKGVNGHRSNFYSVSTSADGKEVKTLVNSVVAQEAVTQIVEVGTMVTHVGDENGQAAIAEE 1245
CAB87223.1_HexA_S. coelicolor  ---------------------------------------------------------------------- 535
AAC38798.3_SpHex_S. plicatus   ---------------------------------------------------------------------- 506
AKC34128.1_Hex1_uncultured Act ---------------------------------------------------------------------- 494
AKC34129.1_Hex2_uncultured Bac ---------------------------------------------------------------------- 537
AAC44672.1_ExoI_V. furnissii   ---------------------------------------------------------------------- 611
AAA88682.1_Chb_V. harveyi      ---------------------------------------------------------------------- 883
AAD30612.1_AtHex1_A. thaliana  ---------------------------------------------------------------------- 580
AAM91092.1_AtHex3_A. thaliana  ---------------------------------------------------------------------- 535
BAE99290.1_AtHex3_A. thaliana  ---------------------------------------------------------------------- 541
AAM13977.1_HexA_A. oryzae      ---------------------------------------------------------------------- 600
AAT99455.1_BmGlcNAcase3_B. mor ---------------------------------------------------------------------- 536
AAC60521.1_BmChiGlcNAcase_B. m ---------------------------------------------------------------------- 596
CCD73477.2_CeHEX-4_C. elegans  ---------------------------------------------------------------------- 491
AAA34346.2_Hex1_C. albicans    ---------------------------------------------------------------------- 562
ADU20405.1_βHex_C. annuum      ---------------------------------------------------------------------- 574
ABB18373.1_CpHEX1_C. posadasii ---------------------------------------------------------------------- 595
AAA33230.1_NagA_D. discoideum  ---------------------------------------------------------------------- 532
AAF46406.1_Hex2_D. melanogaste ---------------------------------------------------------------------- 622
AAF47881.1_Hex1_D. melanogaste ---------------------------------------------------------------------- 622
AAM29423.1_Fdl_D. melanogaster ---------------------------------------------------------------------- 660
CAE46968.1_HexA_E. histolytica ---------------------------------------------------------------------- 564
AAB00965.1_HexA_H. sapiens     ---------------------------------------------------------------------- 529
AAA52645.1_HexB_H. sapiens     ---------------------------------------------------------------------- 556
AAC53246.1_HexA_M. musculus    ---------------------------------------------------------------------- 528
AAA18776.1_HexB_M. musculus    ---------------------------------------------------------------------- 536
ABI81756.1_Hex1_O. furnacalis  ---------------------------------------------------------------------- 594
AEQ33603.1_HexTF_T. flavus     ---------------------------------------------------------------------- 628
ACI15900.1_NagA_T. maritima    ---------------------------------------------------------------------- 467
AAK16587.1_CbsA_T. neapolitana ---------------------------------------------------------------------- 467
CAA63123.1_HexB_S. scrofa      ---------------------------------------------------------------------- 531
```

```
                                          1550      1560      1570      1580      1590      1600      1610
                                 ....|....|....|....|....|....|....|....|....|....|....|....|....|....|
WP_082768773.1_Nah20B_P. hydro   ---------------------------------------------------------------------- 879
WP_068373836.1_Nah20A_P. hydro   ---------------------------------------------------------------------- 807
BAD00143.1_Nah20A_A. hydrophil   ---------------------------------------------------------------------- 887
BAF76001.1_NahB_A. hydrophila    ---------------------------------------------------------------------- 618
BAI94822.1_BbhI_B. bifidum       SDGSTKTLTAGEYTLSATQDGAAVALDKAFAKAGKVTVTVTANGKTATFDVTVTAKDPDPEPATLKSIKV 1485
AAQ05800.1_Hex20A_C. fimi        ---------------------------------------------------------------------- 496
BAI63641.1_Hex1t_Paenibacillus   DELKIFNRALSASEVVELAKSPAPKASLTGPQSANPGQSFDVKMGLSDVSPSEFGQMYAQDWTINYDSAK 752
CAA55582.1_NahA_P. gingivalis    DNVKLIDNVGEVKKFSRRNSSRALQKGYHPIKTIWVGAIQGAWPTYWNYSRVMIRLKGEEKFKPISSDML 775
BAA84281.1_Hex99_P. piscicida    ---------------------------------------------------------------------- 914
BAB84321.1_Hex86_P. piscicida    TLAEVNNPRGPMSVLSFAPVSVRFLKVVAINSKQSDDIQIPKLPLYIDEIAVY----------------- 761
AAB03808.1_Chb_S. marcescens     ---------------------------------------------------------------------- 885
AAS02154.1_GcnA_S. gordonii      ---------------------------------------------------------------------- 627
AAK98861.1_StrH_S. pneumoniae    KPKLEIPSQPAPSTAPAEESKALPQDPAPVVTEKKLPETGTHDSAELVVAGLMSTLAAYGLTKRKED--- 1312
CAB87223.1_HexA_S. coelicolor    ---------------------------------------------------------------------- 535
AAC38798.3_SpHex_S. plicatus     ---------------------------------------------------------------------- 506
AKC34128.1_Hex1_uncultured Act   ---------------------------------------------------------------------- 494
AKC34129.1_Hex2_uncultured Bac   ---------------------------------------------------------------------- 537
AAC44672.1_ExoI_V. furnissii     ---------------------------------------------------------------------- 611
AAA88682.1_Chb_V. harveyi        ---------------------------------------------------------------------- 883
AAD30612.1_AtHex1_A. thaliana    ---------------------------------------------------------------------- 580
AAM91092.1_AtHex3_A. thaliana    ---------------------------------------------------------------------- 535
BAE99290.1_AtHex3_A. thaliana    ---------------------------------------------------------------------- 541
AAM13977.1_HexA_A. oryzae        ---------------------------------------------------------------------- 600
AAT99455.1_BmGlcNAcase3_B. mor   ---------------------------------------------------------------------- 536
AAC60521.1_BmChiGlcNAcase_B. m   ---------------------------------------------------------------------- 596
CCD73477.2_CeHEX-4_C. elegans    ---------------------------------------------------------------------- 491
AAA34346.2_Hex1_C. albicans      ---------------------------------------------------------------------- 562
ADU20405.1_βHex_C. annuum        ---------------------------------------------------------------------- 574
ABB18373.1_CpHEX1_C. posadasii   ---------------------------------------------------------------------- 595
AAA33230.1_NagA_D. discoideum    ---------------------------------------------------------------------- 532
AAF46406.1_Hex2_D. melanogaste   ---------------------------------------------------------------------- 622
AAF47881.1_Hex1_D. melanogaste   ---------------------------------------------------------------------- 622
AAM29423.1_Fdl_D. melanogaster   ---------------------------------------------------------------------- 660
CAE46968.1_HexA_E. histolytica   ---------------------------------------------------------------------- 564
AAB00965.1_HexA_H. sapiens       ---------------------------------------------------------------------- 529
AAA52645.1_HexB_H. sapiens       ---------------------------------------------------------------------- 556
AAC53246.1_HexA_M. musculus      ---------------------------------------------------------------------- 528
AAA18776.1_HexB_M. musculus      ---------------------------------------------------------------------- 536
ABI81756.1_Hex1_O. furnacalis    ---------------------------------------------------------------------- 594
AEQ33603.1_HexTF_T. flavus       ---------------------------------------------------------------------- 628
ACI15900.1_NagA_T. maritima      ---------------------------------------------------------------------- 467
AAK16587.1_CbsA_T. neapolitana   ---------------------------------------------------------------------- 467
CAA63123.1_HexB_S. scrofa        ---------------------------------------------------------------------- 531
```

```
                                      1620      1630      1640      1650      1660      1670      1680
                                ....|....|....|....|....|....|....|....|....|....|....|....|....|....|
WP_082768773.1_Nah20B_P. hydro  ---------------------------------------------------------------------- 879
WP_068373836.1_Nah20A_P. hydro  ---------------------------------------------------------------------- 807
BAD00143.1_Nah20A_A. hydrophil  ---------------------------------------------------------------------- 887
BAF76001.1_NahB_A. hydrophila   ---------------------------------------------------------------------- 618
BAI94822.1_BbhI_B. bifidum      TSKPDKTTYTVDETFAKTGLAVTGTWSDGKTALLKDGEYKLSAVDADGKTVDLTKPFTAAGDVTVTVTSG 1555
AAQ05800.1_Hex20A_C. fimi       ---------------------------------------------------------------------- 496
BAI63641.1_Hex1t_Paenibacillus  LQLDSITSLQDKFQVIDQKELAPGQIRIVAANAAANQGVTPQGDLFAFKFTVKAGTDVKTTISADHIVIG 822
CAA55582.1_NahA_P. gingivalis   FQ-------------------------------------------------------------------- 777
BAA84281.1_Hex99_P. piscicida   ---------------------------------------------------------------------- 914
BAB84321.1_Hex86_P. piscicida   ---------------------------------------------------------------------- 761
AAB03808.1_Chb_S. marcescens    ---------------------------------------------------------------------- 885
AAS02154.1_GcnA_S. gordonii     ---------------------------------------------------------------------- 627
AAK98861.1_StrH_S. pneumoniae   ---------------------------------------------------------------------- 1312
CAB87223.1_HexA_S. coelicolor   ---------------------------------------------------------------------- 535
AAC38798.3_SpHex_S. plicatus    ---------------------------------------------------------------------- 506
AKC34128.1_Hex1_uncultured Act  ---------------------------------------------------------------------- 494
AKC34129.1_Hex2_uncultured Bac  ---------------------------------------------------------------------- 537
AAC44672.1_ExoI_V. furnissii    ---------------------------------------------------------------------- 611
AAA88682.1_Chb_V. harveyi       ---------------------------------------------------------------------- 883
AAD30612.1_AtHex1_A. thaliana   ---------------------------------------------------------------------- 580
AAM91092.1_AtHex3_A. thaliana   ---------------------------------------------------------------------- 535
BAE99290.1_AtHex3_A. thaliana   ---------------------------------------------------------------------- 541
AAM13977.1_HexA_A. oryzae       ---------------------------------------------------------------------- 600
AAT99455.1_BmGlcNAcase3_B. mor  ---------------------------------------------------------------------- 536
AAC60521.1_BmChiGlcNAcase_B. m  ---------------------------------------------------------------------- 596
CCD73477.2_CeHEX-4_C. elegans   ---------------------------------------------------------------------- 491
AAA34346.2_Hex1_C. albicans     ---------------------------------------------------------------------- 562
ADU20405.1_β Hex_C. annuum      ---------------------------------------------------------------------- 574
ABB18373.1_CpHEX1_C. posadasii  ---------------------------------------------------------------------- 595
AAA33230.1_NagA_D. discoideum   ---------------------------------------------------------------------- 532
AAF46406.1_Hex2_D. melanogaste  ---------------------------------------------------------------------- 622
AAF47881.1_Hex1_D. melanogaste  ---------------------------------------------------------------------- 622
AAM29423.1_Fdl_D. melanogaster  ---------------------------------------------------------------------- 660
CAE46968.1_HexA_E. histolytica  ---------------------------------------------------------------------- 564
AAB00965.1_HexA_H. sapiens      ---------------------------------------------------------------------- 529
AAA52645.1_HexB_H. sapiens      ---------------------------------------------------------------------- 556
AAC53246.1_HexA_M. musculus     ---------------------------------------------------------------------- 528
AAA18776.1_HexB_M. musculus     ---------------------------------------------------------------------- 536
ABI81756.1_Hex1_O. furnacalis   ---------------------------------------------------------------------- 594
AEQ33603.1_HexTF_T. flavus      ---------------------------------------------------------------------- 628
ACI15900.1_NagA_T. maritima     ---------------------------------------------------------------------- 467
AAK16587.1_CbsA_T. neapolitana  ---------------------------------------------------------------------- 467
CAA63123.1_HexB_S. scrofa       ---------------------------------------------------------------------- 531
```

```
                                         1690      1700      1710      1720      1730      1740      1750
                                 ....|....|....|....|....|....|....|....|....|....|....|....|....|....|
WP_082768773.1_Nah20B_P. hydro   ---------------------------------------------------------------------- 879
WP_068373836.1_Nah20A_P. hydro   ---------------------------------------------------------------------- 807
BAD00143.1_Nah20A_A. hydrophil   ---------------------------------------------------------------------- 887
BAF76001.1_NahB_A. hydrophila    ---------------------------------------------------------------------- 618
BAI94822.1_BbhI_B. bifidum       KLTDSFTITVKAKTVTPAPGDNKPGENKPGADKPKPNTPDEVAKTGASVTAVVFSALLLLSAGYLLVRKR 1625
AAQ05800.1_Hex20A_C. fimi        ---------------------------------------------------------------------- 496
BAI63641.1_Hex1t_Paenibacillus   NAQGKELEIAGATHEIQVSIPVDKSQLNVLIANAQAKHDAAVEGNEDGLYAAGSKAQLQTAIHTAKAVAD 892
CAA55582.1_NahA_P. gingivalis    ---------------------------------------------------------------------- 777
BAA84281.1_Hex99_P. piscicida    ---------------------------------------------------------------------- 914
BAB84321.1_Hex86_P. piscicida    ---------------------------------------------------------------------- 761
AAB03808.1_Chb_S. marcescens     ---------------------------------------------------------------------- 885
AAS02154.1_GcnA_S. gordonii      ---------------------------------------------------------------------- 627
AAK98861.1_StrH_S. pneumoniae    ---------------------------------------------------------------------- 1312
CAB87223.1_HexA_S. coelicolor    ---------------------------------------------------------------------- 535
AAC38798.3_SpHex_S. plicatus     ---------------------------------------------------------------------- 506
AKC34128.1_Hex1_uncultured Act   ---------------------------------------------------------------------- 494
AKC34129.1_Hex2_uncultured Bac   ---------------------------------------------------------------------- 537
AAC44672.1_ExoI_V. furnissii     ---------------------------------------------------------------------- 611
AAA88682.1_Chb_V. harveyi        ---------------------------------------------------------------------- 883
AAD30612.1_AtHex1_A. thaliana    ---------------------------------------------------------------------- 580
AAM91092.1_AtHex3_A. thaliana    ---------------------------------------------------------------------- 535
BAE99290.1_AtHex3_A. thaliana    ---------------------------------------------------------------------- 541
AAM13977.1_HexA_A. oryzae        ---------------------------------------------------------------------- 600
AAT99455.1_BmGlcNAcase3_B. mor   ---------------------------------------------------------------------- 536
AAC60521.1_BmChiGlcNAcase_B. m   ---------------------------------------------------------------------- 596
CCD73477.2_CeHEX-4_C. elegans    ---------------------------------------------------------------------- 491
AAA34346.2_Hex1_C. albicans      ---------------------------------------------------------------------- 562
ADU20405.1_βHex_C. annuum        ---------------------------------------------------------------------- 574
ABB18373.1_CpHEX1_C. posadasii   ---------------------------------------------------------------------- 595
AAA33230.1_NagA_D. discoideum    ---------------------------------------------------------------------- 532
AAF46406.1_Hex2_D. melanogaste   ---------------------------------------------------------------------- 622
AAF47881.1_Hex1_D. melanogaste   ---------------------------------------------------------------------- 622
AAM29423.1_Fdl_D. melanogaster   ---------------------------------------------------------------------- 660
CAE46968.1_HexA_E. histolytica   ---------------------------------------------------------------------- 564
AAB00965.1_HexA_H. sapiens       ---------------------------------------------------------------------- 529
AAA52645.1_HexB_H. sapiens       ---------------------------------------------------------------------- 556
AAC53246.1_HexA_M. musculus      ---------------------------------------------------------------------- 528
AAA18776.1_HexB_M. musculus      ---------------------------------------------------------------------- 536
ABI81756.1_Hex1_O. furnacalis    ---------------------------------------------------------------------- 594
AEQ33603.1_HexTF_T. flavus       ---------------------------------------------------------------------- 628
ACI15900.1_NagA_T. maritima      ---------------------------------------------------------------------- 467
AAK16587.1_CbsA_T. neapolitana   ---------------------------------------------------------------------- 467
CAA63123.1_HexB_S. scrofa        ---------------------------------------------------------------------- 531
```

```
                                                1760      1770      1780      1790      1800      1810      1820
                                       ....|....|....|....|....|....|....|....|....|....|....|....|....|....|
WP_082768773.1_Nah20B_P. hydro         ---------------------------------------------------------------------- 879
WP_068373836.1_Nah20A_P. hydro         ---------------------------------------------------------------------- 807
BAD00143.1_Nah20A_A. hydrophil         ---------------------------------------------------------------------- 887
BAF76001.1_NahB_A. hydrophila          ---------------------------------------------------------------------- 618
BAI94822.1_BbhI_B. bifidum             RI-------------------------------------------------------------------- 1627
AAQ05800.1_Hex20A_C. fimi              ---------------------------------------------------------------------- 496
BAI63641.1_Hex1t_Paenibacillus         NSNASQQQVDSAKSALEEAVQVFESKKISADVNGDGQVSIGDLAIIAGAYGKEEGQAGWNKKADVNHDGK 962
CAA55582.1_NahA_P. gingivalis          ---------------------------------------------------------------------- 777
BAA84281.1_Hex99_P. piscicida          ---------------------------------------------------------------------- 914
BAB84321.1_Hex86_P. piscicida          ---------------------------------------------------------------------- 761
AAB03808.1_Chb_S. marcescens           ---------------------------------------------------------------------- 885
AAS02154.1_GcnA_S. gordonii            ---------------------------------------------------------------------- 627
AAK98861.1_StrH_S. pneumoniae          ---------------------------------------------------------------------- 1312
CAB87223.1_HexA_S. coelicolor          ---------------------------------------------------------------------- 535
AAC38798.3_SpHex_S. plicatus           ---------------------------------------------------------------------- 506
AKC34128.1_Hex1_uncultured Act         ---------------------------------------------------------------------- 494
AKC34129.1_Hex2_uncultured Bac         ---------------------------------------------------------------------- 537
AAC44672.1_ExoI_V. furnissii           ---------------------------------------------------------------------- 611
AAA88682.1_Chb_V. harveyi              ---------------------------------------------------------------------- 883
AAD30612.1_AtHex1_A. thaliana          ---------------------------------------------------------------------- 580
AAM91092.1_AtHex3_A. thaliana          ---------------------------------------------------------------------- 535
BAE99290.1_AtHex3_A. thaliana          ---------------------------------------------------------------------- 541
AAM13977.1_HexA_A. oryzae              ---------------------------------------------------------------------- 600
AAT99455.1_BmGlcNAcase3_B. mor         ---------------------------------------------------------------------- 536
AAC60521.1_BmChiGlcNAcase_B. m         ---------------------------------------------------------------------- 596
CCD73477.2_CeHEX-4_C. elegans          ---------------------------------------------------------------------- 491
AAA34346.2_Hex1_C. albicans            ---------------------------------------------------------------------- 562
ADU20405.1_βHex_C. annuum              ---------------------------------------------------------------------- 574
ABB18373.1_CpHEX1_C. posadasii         ---------------------------------------------------------------------- 595
AAA33230.1_NagA_D. discoideum          ---------------------------------------------------------------------- 532
AAF46406.1_Hex2_D. melanogaste         ---------------------------------------------------------------------- 622
AAF47881.1_Hex1_D. melanogaste         ---------------------------------------------------------------------- 622
AAM29423.1_Fdl_D. melanogaster         ---------------------------------------------------------------------- 660
CAE46968.1_HexA_E. histolytica         ---------------------------------------------------------------------- 564
AAB00965.1_HexA_H. sapiens             ---------------------------------------------------------------------- 529
AAA52645.1_HexB_H. sapiens             ---------------------------------------------------------------------- 556
AAC53246.1_HexA_M. musculus            ---------------------------------------------------------------------- 528
AAA18776.1_HexB_M. musculus            ---------------------------------------------------------------------- 536
ABI81756.1_Hex1_O. furnacalis          ---------------------------------------------------------------------- 594
AEQ33603.1_HexTF_T. flavus             ---------------------------------------------------------------------- 628
ACI15900.1_NagA_T. maritima            ---------------------------------------------------------------------- 467
AAK16587.1_CbsA_T. neapolitana         ---------------------------------------------------------------------- 467
CAA63123.1_HexB_S. scrofa              ---------------------------------------------------------------------- 531
```

```
                                               1830
                                         ....|....|....|.
WP_082768773.1_Nah20B_P. hydro           ---------------- 879
WP_068373836.1_Nah20A_P. hydro           ---------------- 807
BAD00143.1_Nah20A_A. hydrophil           ---------------- 887
BAF76001.1_NahB_A. hydrophila            ---------------- 618
BAI94822.1_BbhI_B. bifidum               ---------------- 1627
AAQ05800.1_Hex20A_C. fimi                ---------------- 496
BAI63641.1_Hex1t_Paenibacillus           VDIIDLTIVAKAILQI 978
CAA55582.1_NahA_P. gingivalis            ---------------- 777
BAA84281.1_Hex99_P. piscicida            ---------------- 914
BAB84321.1_Hex86_P. piscicida            ---------------- 761
AAB03808.1_Chb_S. marcescens             ---------------- 885
AAS02154.1_GcnA_S. gordonii              ---------------- 627
AAK98861.1_StrH_S. pneumoniae            ---------------- 1312
CAB87223.1_HexA_S. coelicolor            ---------------- 535
AAC38798.3_SpHex_S. plicatus             ---------------- 506
AKC34128.1_Hex1_uncultured Act           ---------------- 494
AKC34129.1_Hex2_uncultured Bac           ---------------- 537
AAC44672.1_ExoI_V. furnissii             ---------------- 611
AAA88682.1_Chb_V. harveyi                ---------------- 883
AAD30612.1_AtHex1_A. thaliana            ---------------- 580
AAM91092.1_AtHex3_A. thaliana            ---------------- 535
BAE99290.1_AtHex3_A. thaliana            ---------------- 541
AAM13977.1_HexA_A. oryzae                ---------------- 600
AAT99455.1_BmGlcNAcase3_B. mor           ---------------- 536
AAC60521.1_BmChiGlcNAcase_B. m           ---------------- 596
CCD73477.2_CeHEX-4_C. elegans            ---------------- 491
AAA34346.2_Hex1_C. albicans              ---------------- 562
ADU20405.1_βHex_C. annuum                ---------------- 574
ABB18373.1_CpHEX1_C. posadasii           ---------------- 595
AAA33230.1_NagA_D. discoideum            ---------------- 532
AAF46406.1_Hex2_D. melanogaste           ---------------- 622
AAF47881.1_Hex1_D. melanogaste           ---------------- 622
AAM29423.1_Fdl_D. melanogaster           ---------------- 660
CAE46968.1_HexA_E. histolytica           ---------------- 564
AAB00965.1_HexA_H. sapiens               ---------------- 529
AAA52645.1_HexB_H. sapiens               ---------------- 556
AAC53246.1_HexA_M. musculus              ---------------- 528
AAA18776.1_HexB_M. musculus              ---------------- 536
ABI81756.1_Hex1_O. furnacalis            ---------------- 594
AEQ33603.1_HexTF_T. flavus               ---------------- 628
ACI15900.1_NagA_T. maritima              ---------------- 467
AAK16587.1_CbsA_T. neapolitana           ---------------- 467
CAA63123.1_HexB_S. scrofa                ---------------- 531
```

1. Mark, B.L.; Wasney, G.A; Salo, T.J.; Khan, A.R.; Cao, Z.; Robbins, P.W.; James, M.N.; Triggs-Raine, B.L. Structural and functional characterization of *Streptomyces plicatus* beta-*N*-acetylhexosaminidase by comparative molecular modeling and site-directed mutagenesis. *J. Biol. Chem.* **1998**, *273*, 19618–19624.
